# Supplementary material for: 4,5-Bis(arylethynyl)-1,2,3-triazoles—A New Class of Fluorescent Labels: Synthesis and Applications
Source: Molecules. 2022 May 17;27(10):3191. doi: 10.3390/molecules27103191 (PMC9147796; doi:10.3390/molecules27103191)

Supplementary Information

4,5-Bis(arylethynyl)-1,2,3-triazoles—A New Class of Fluorescent Labels: Synthesis and Applications

Anastasia I. Govdi [1,*], Polina V. Tokareva [1], Andrey M. Rumyantsev [2], Maxim S. Panov [1], Johannes Stellmacher [3], Ulrike Alexiev [3], Natalia A. Danilkina [1] and Irina A. Balova [1,*]

[1] Institute of Chemistry, Saint Petersburg State University (SPbU), Universitetskaya nab. 7/9, 199034 Saint Petersburg, Russia; tokareva.spbu@gmail.com (P.V.T.); m.s.panov@spbu.ru (M.S.P.); danilkina.natalia@gmail.com (N.A.D.)

[2] Department of Genetics and Biotechnology, Saint Petersburg State University (SPbU), Universitetskaya nab. 7/9, 199034 Saint Petersburg, Russia; rumyantsev-am@mail.ru

[3] Department of Physics, Institute of Experimental Physics, Freie Universität Berlin, Arnimalllee 14, 14195 Berlin, Germany; jcart@zedat.fu-berlin.de (J.S.); ulrike.alexiev@fu-berlin.de (U.A.)

* Correspondence: a.govdi@spbu.ru (A.I.G.); i.balova@spbu.ru (I.A.B.); Tel.: +7-812-428-4054 (A.I.G.); +7-812-428-6733 (I.A.B.)

^{1}H NMR ($CDCl_3$, 400.13 MHz)

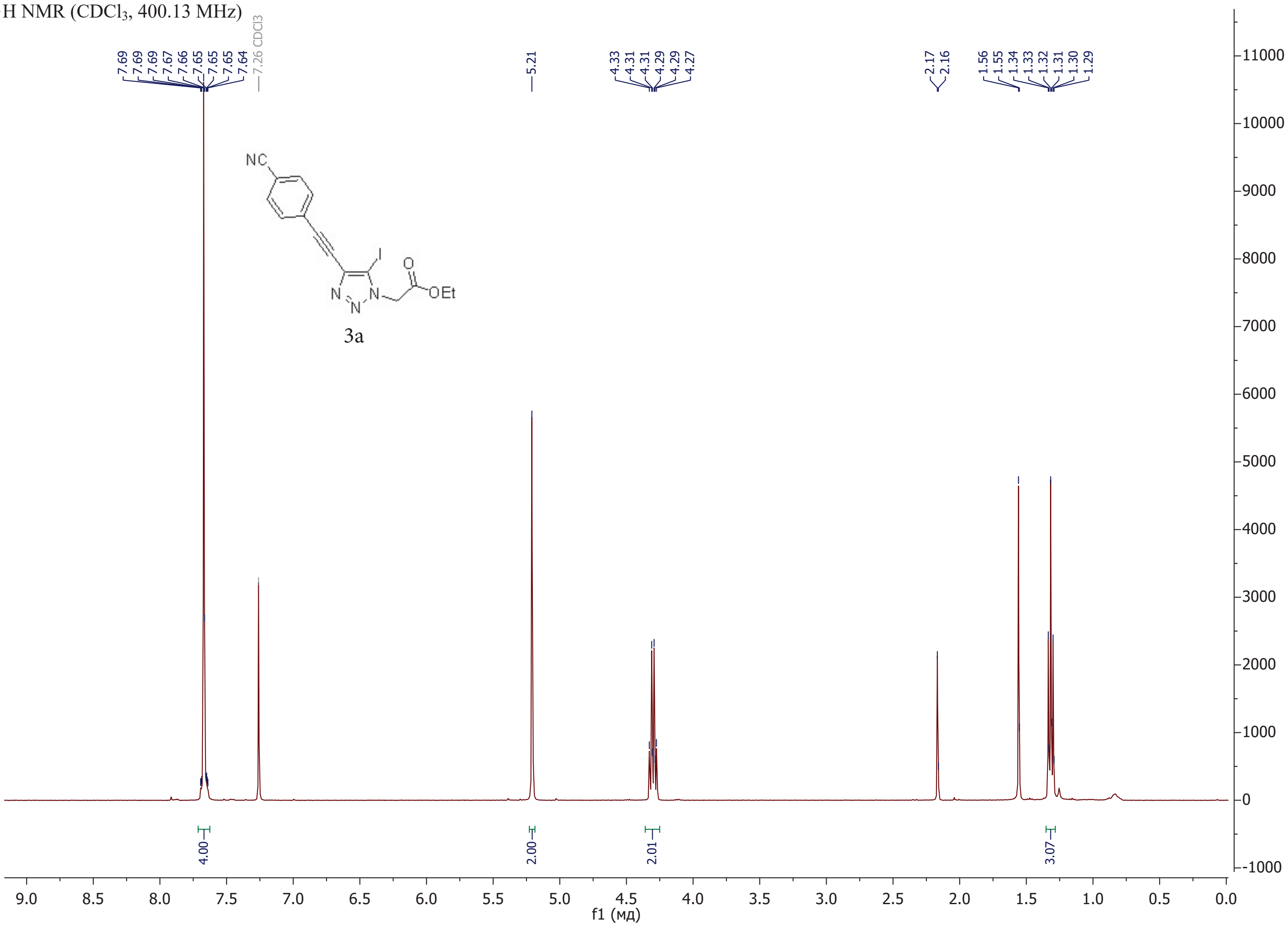

^{13}C NMR ($CDCl_3$, 101 MHz)

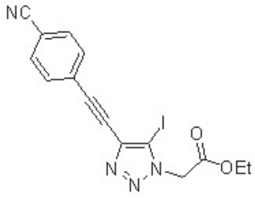

3a

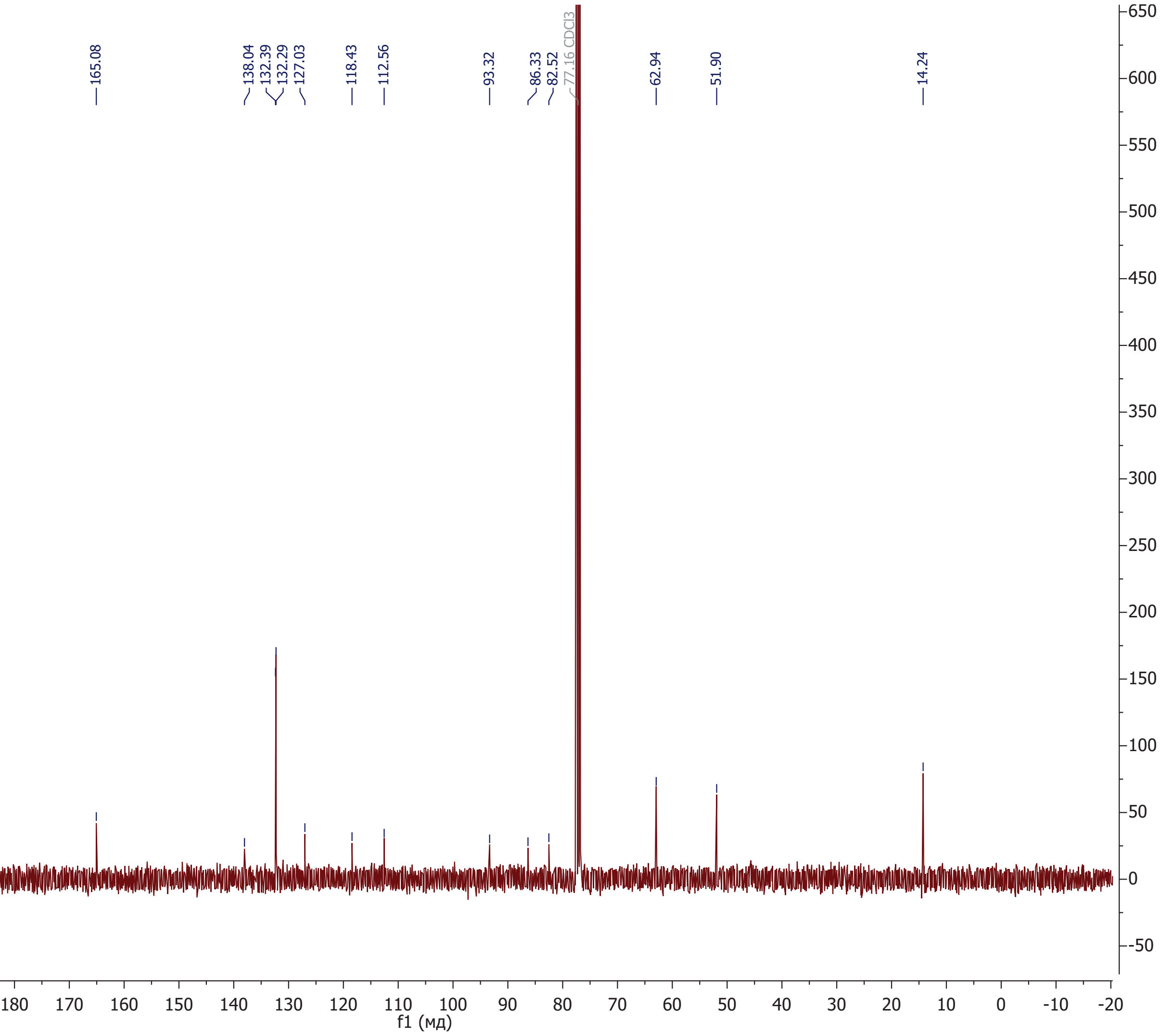

^{13}C NMR ($CDCl_3$, 101 MHz)

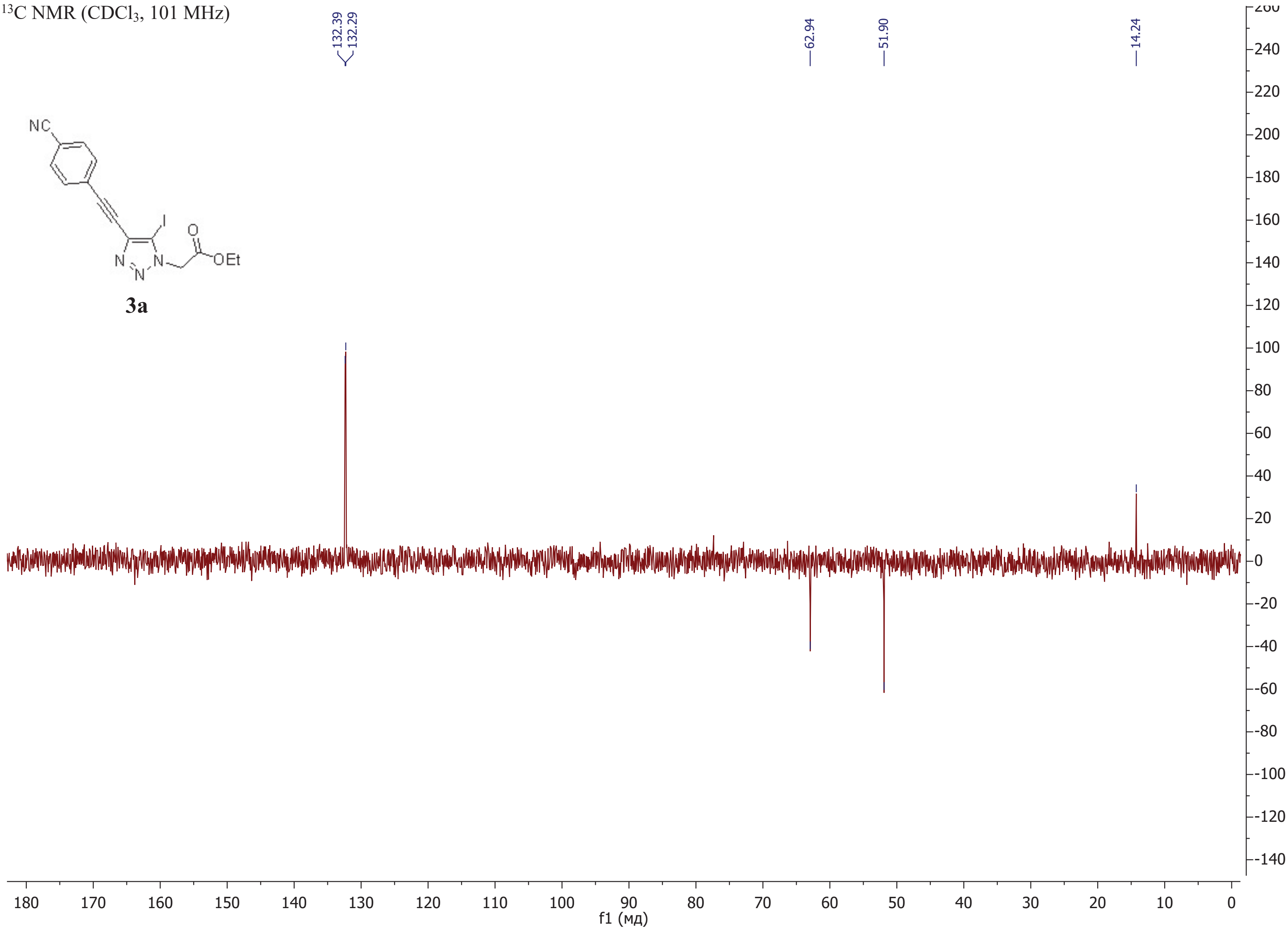

3a

^{1}H NMR ($CDCl_3$, 400 MHz)

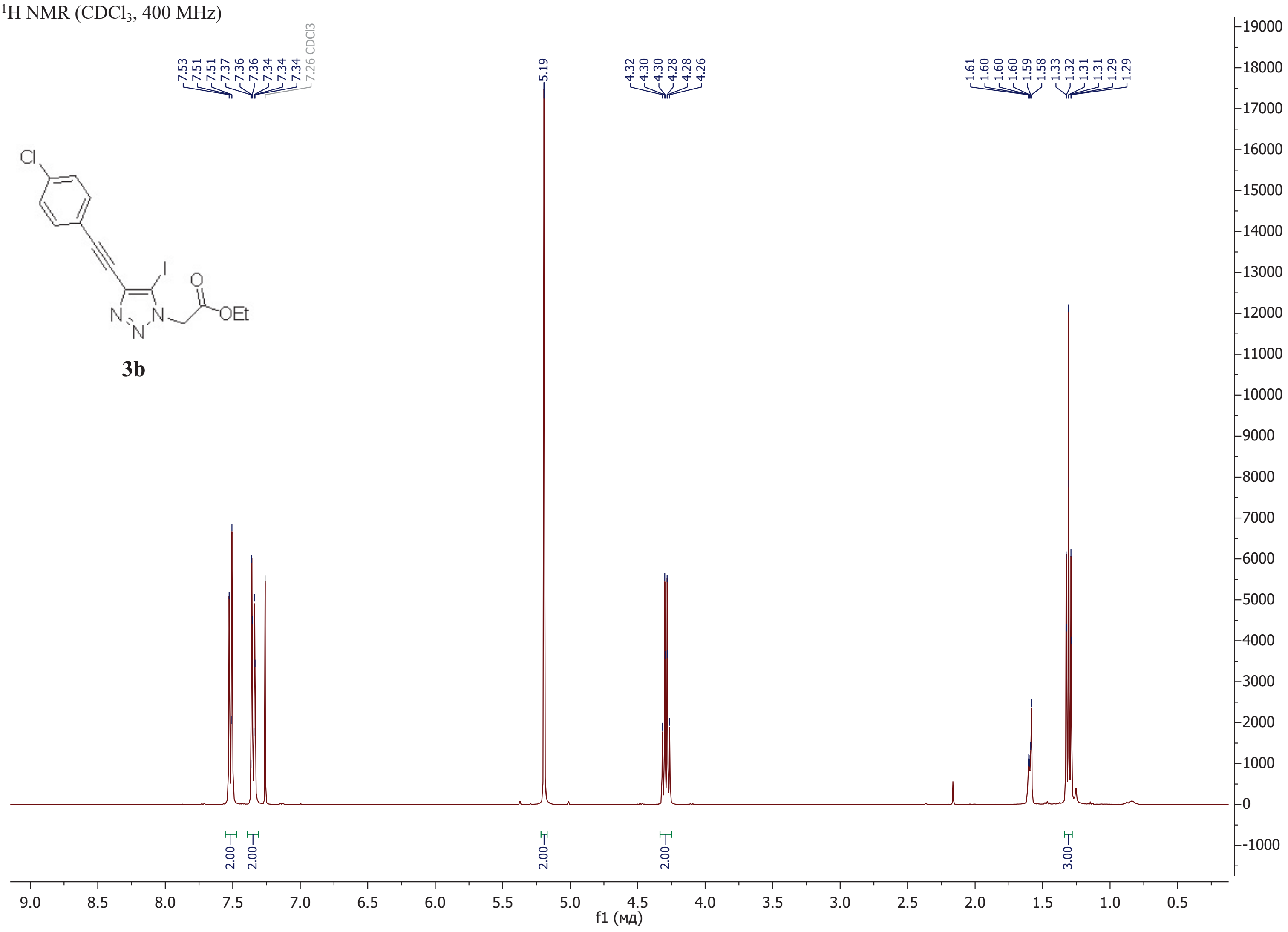

3b

^{13}C NMR ($CDCl_3$, 101 MHz)

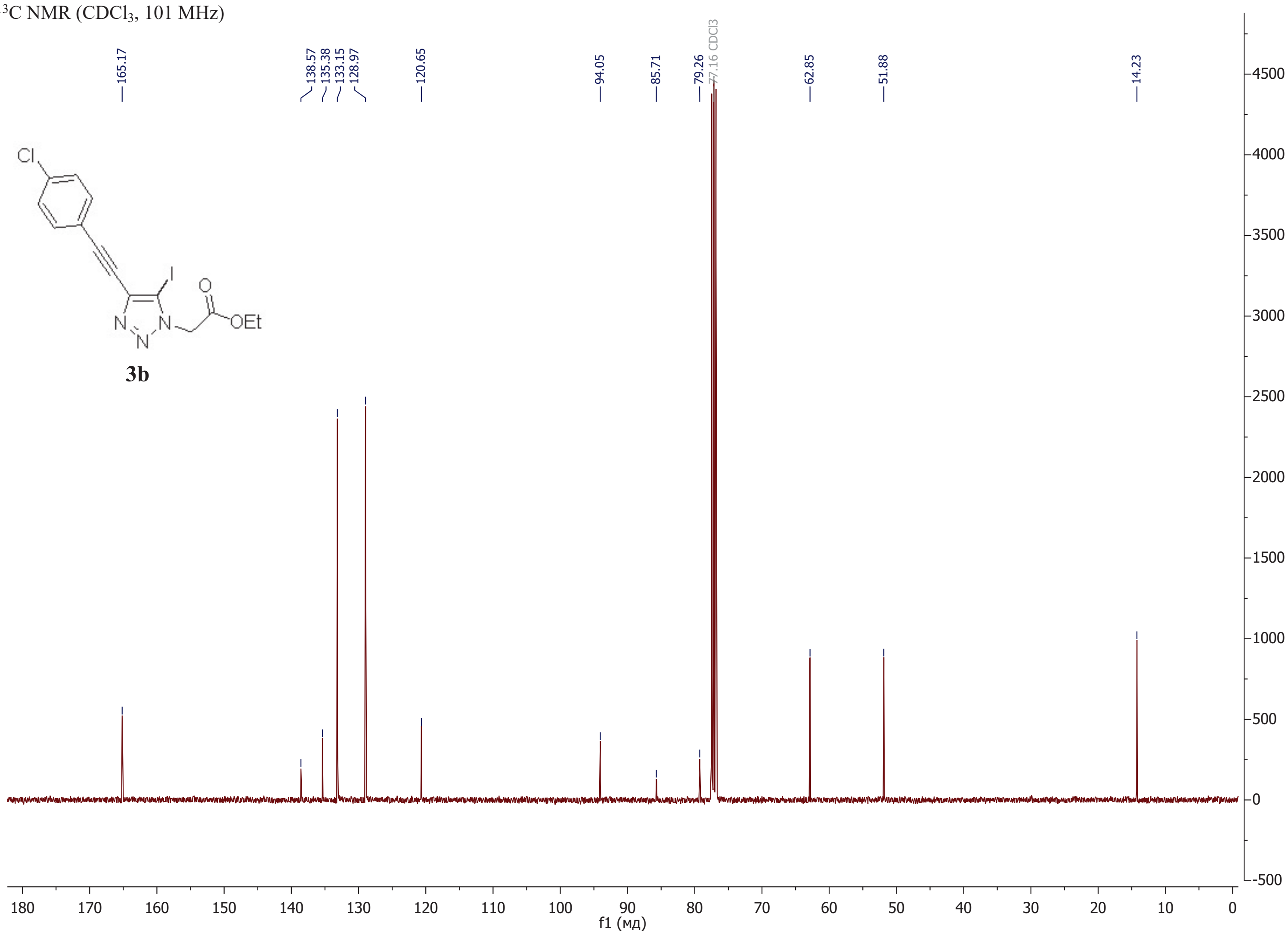

^{13}C NMR ($CDCl_3$, 101 MHz)

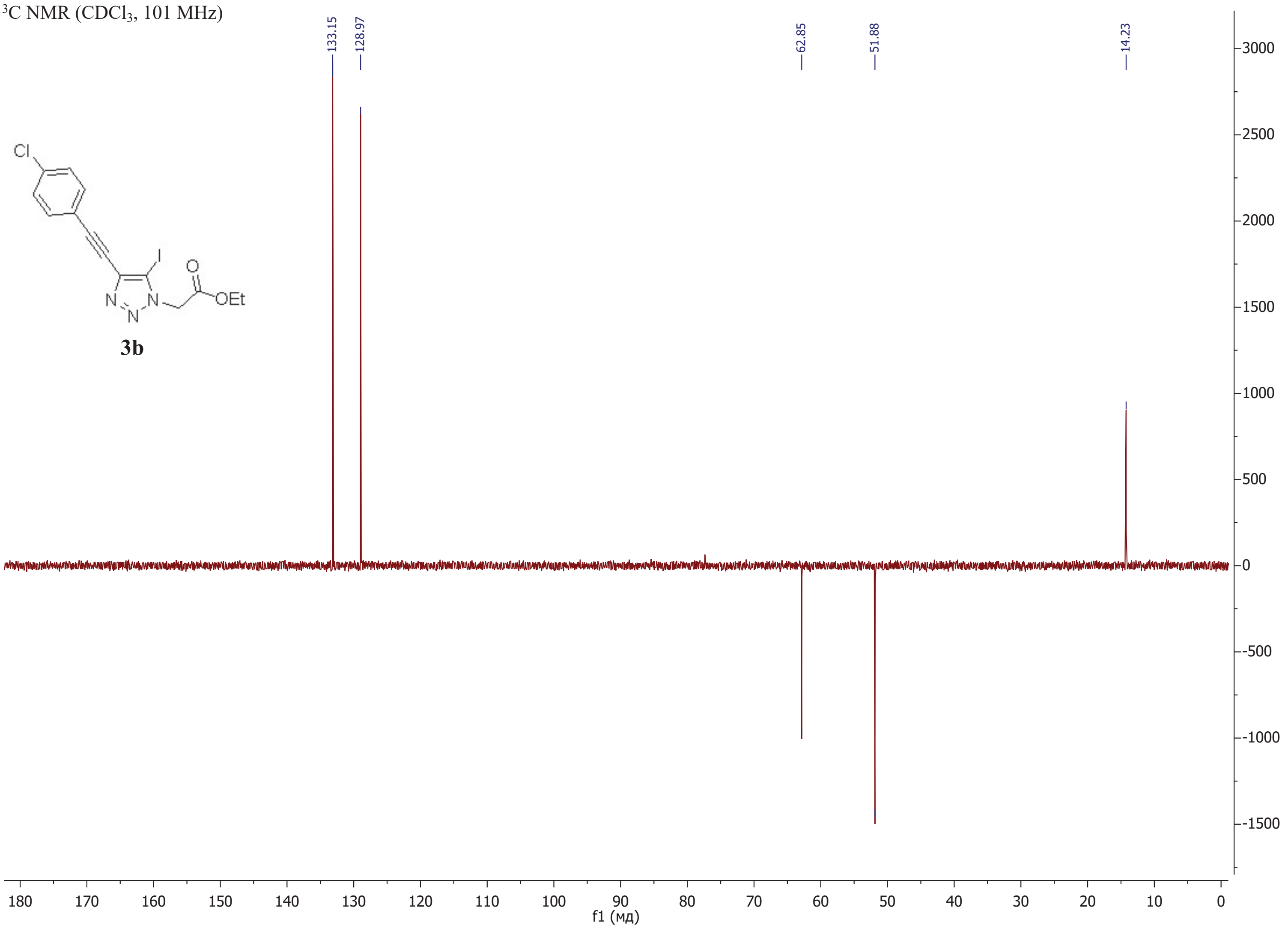

3b

^{1}H NMR ($CDCl_3$, 400 MHz)

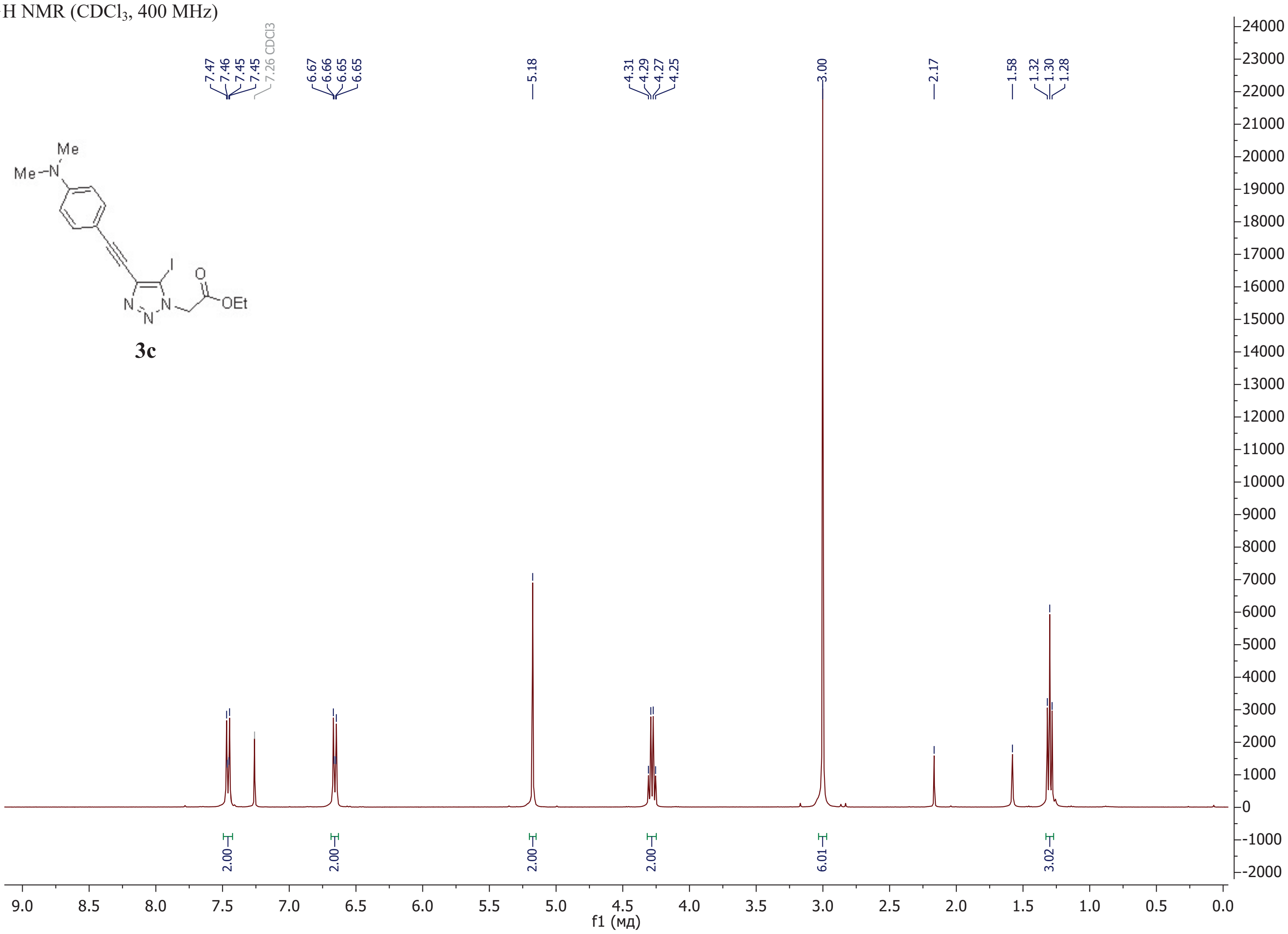

^{13}C NMR (CDCl$_3$, 101 MHz)

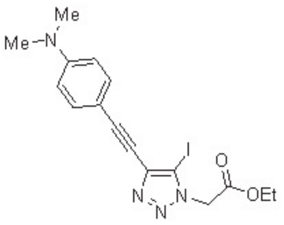

3c

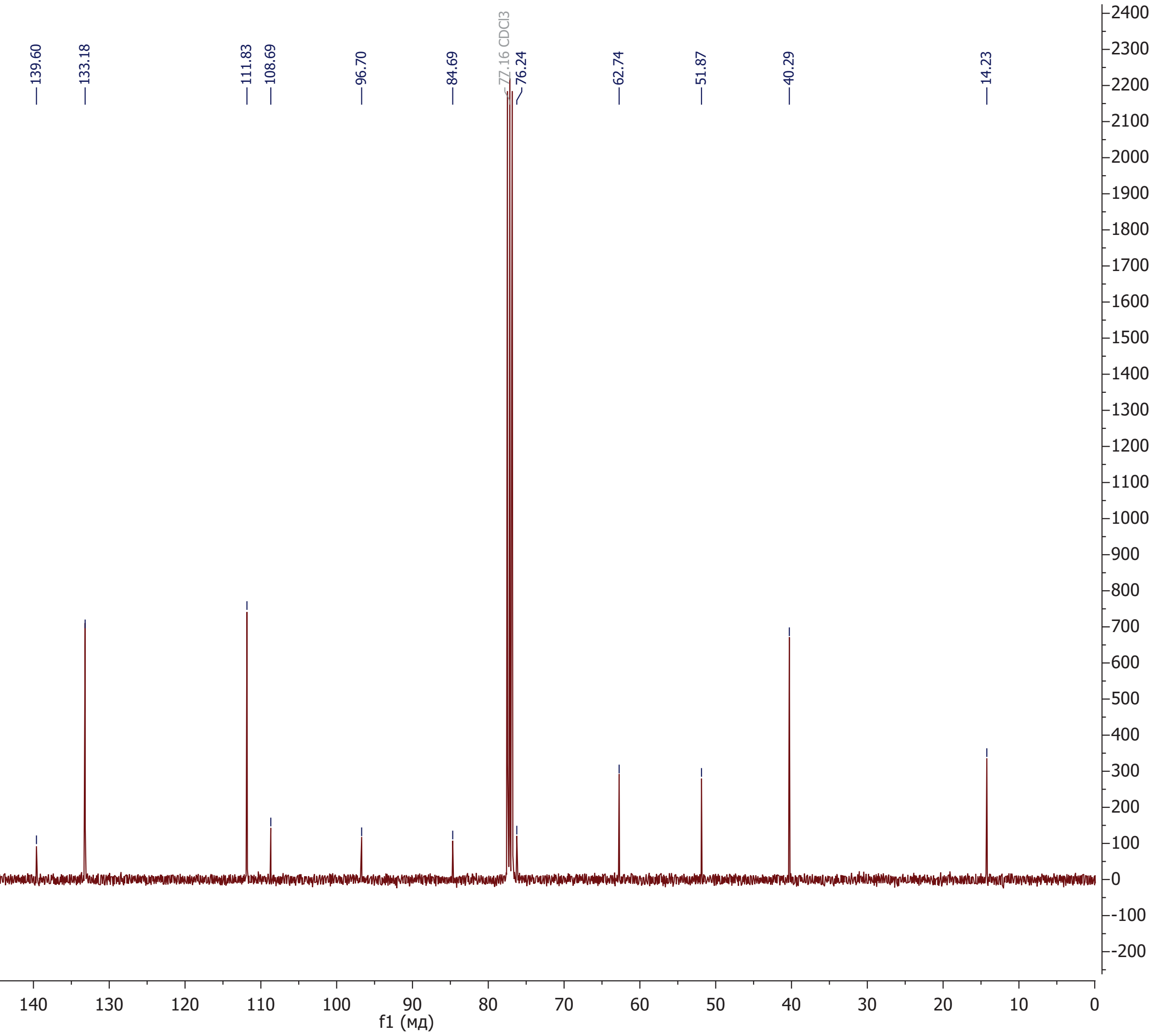

^{13}C NMR ($CDCl_3$, 101 MHz)

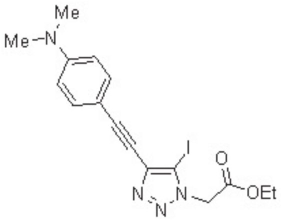

3c

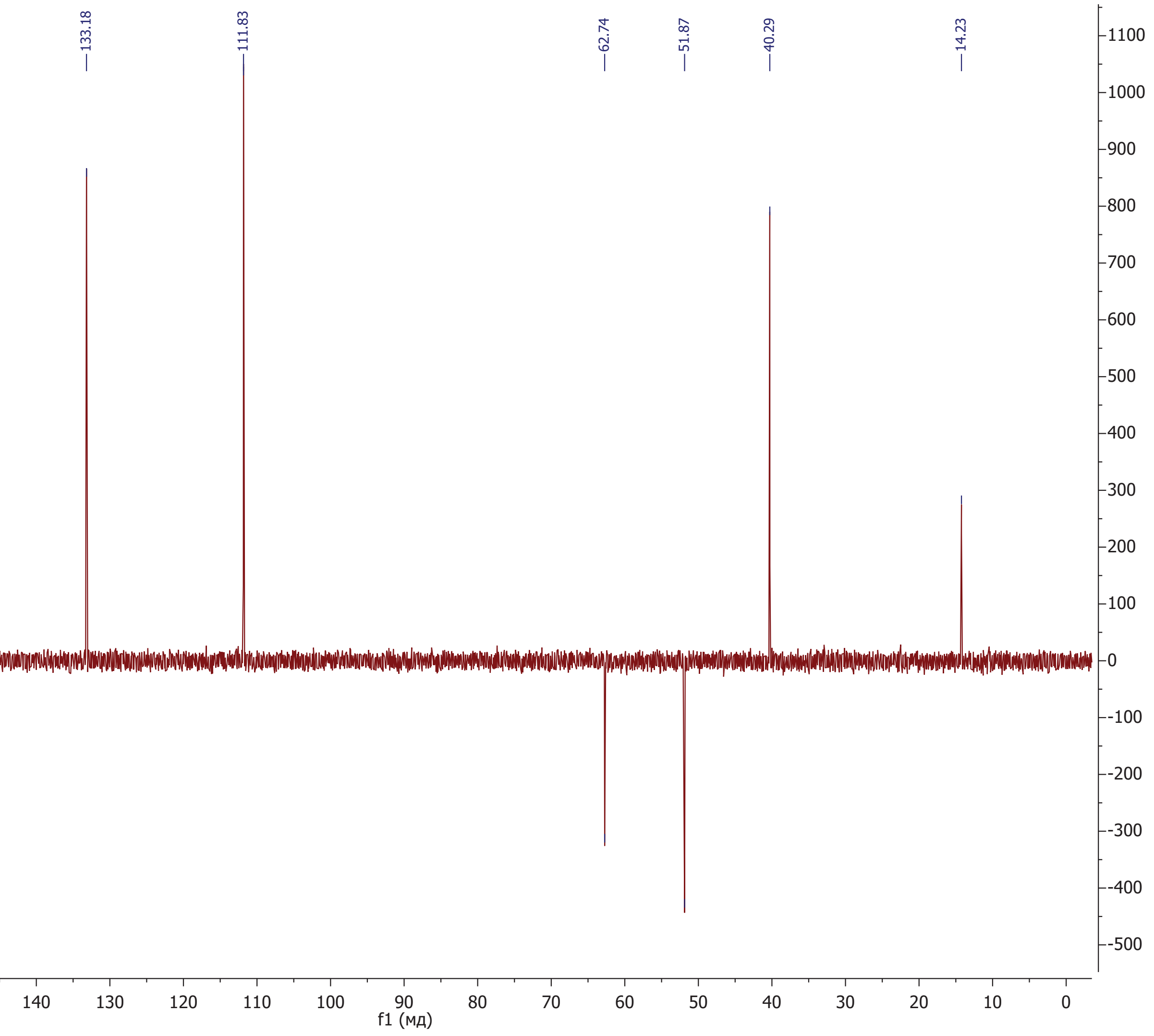

^{1}H NMR ($CDCl_3$, 400 MHz)

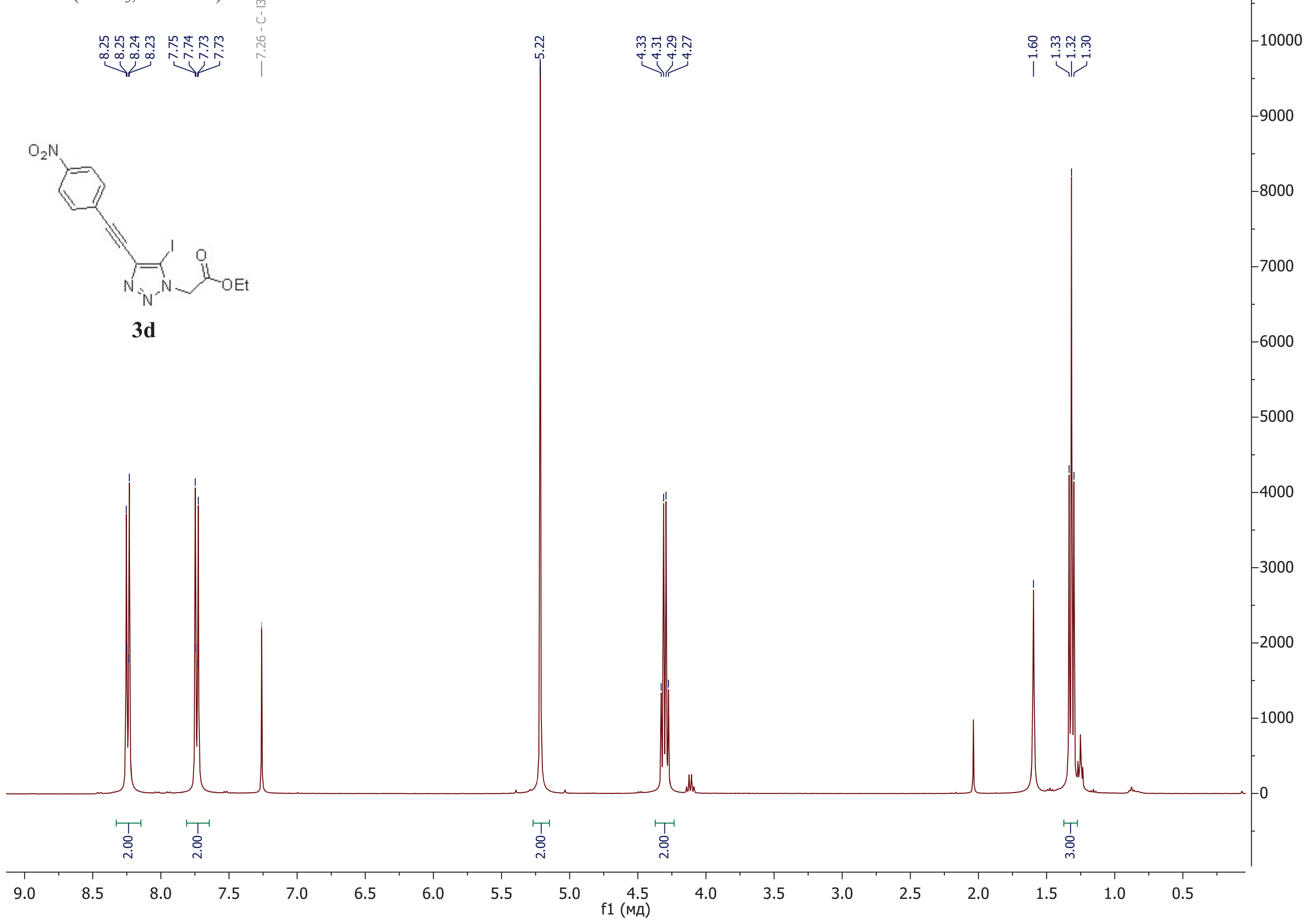

^{13}C NMR ($CDCl_3$, 101 MHz)

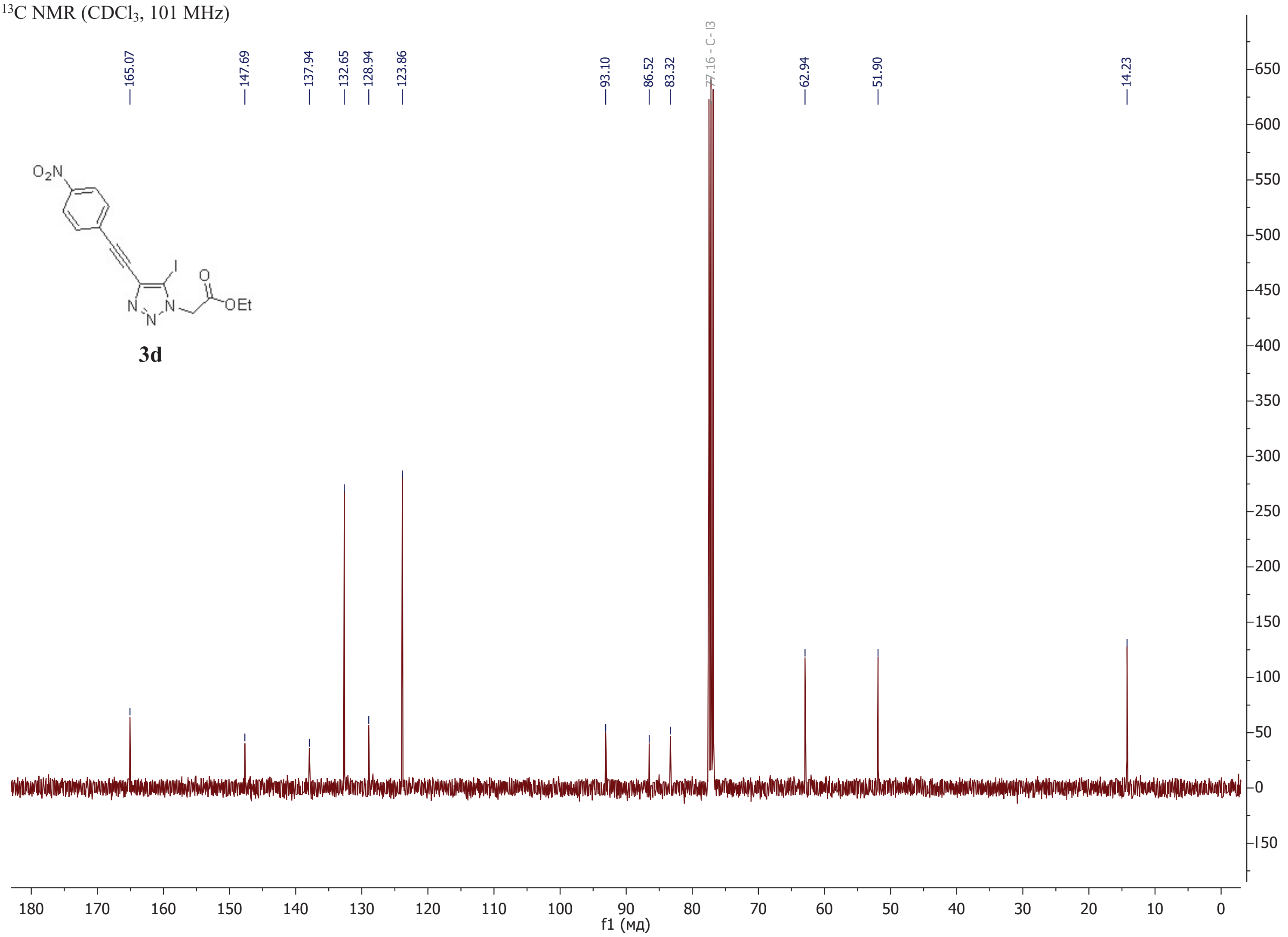

^{1}H NMR ($CDCl_3$, 400 MHz)

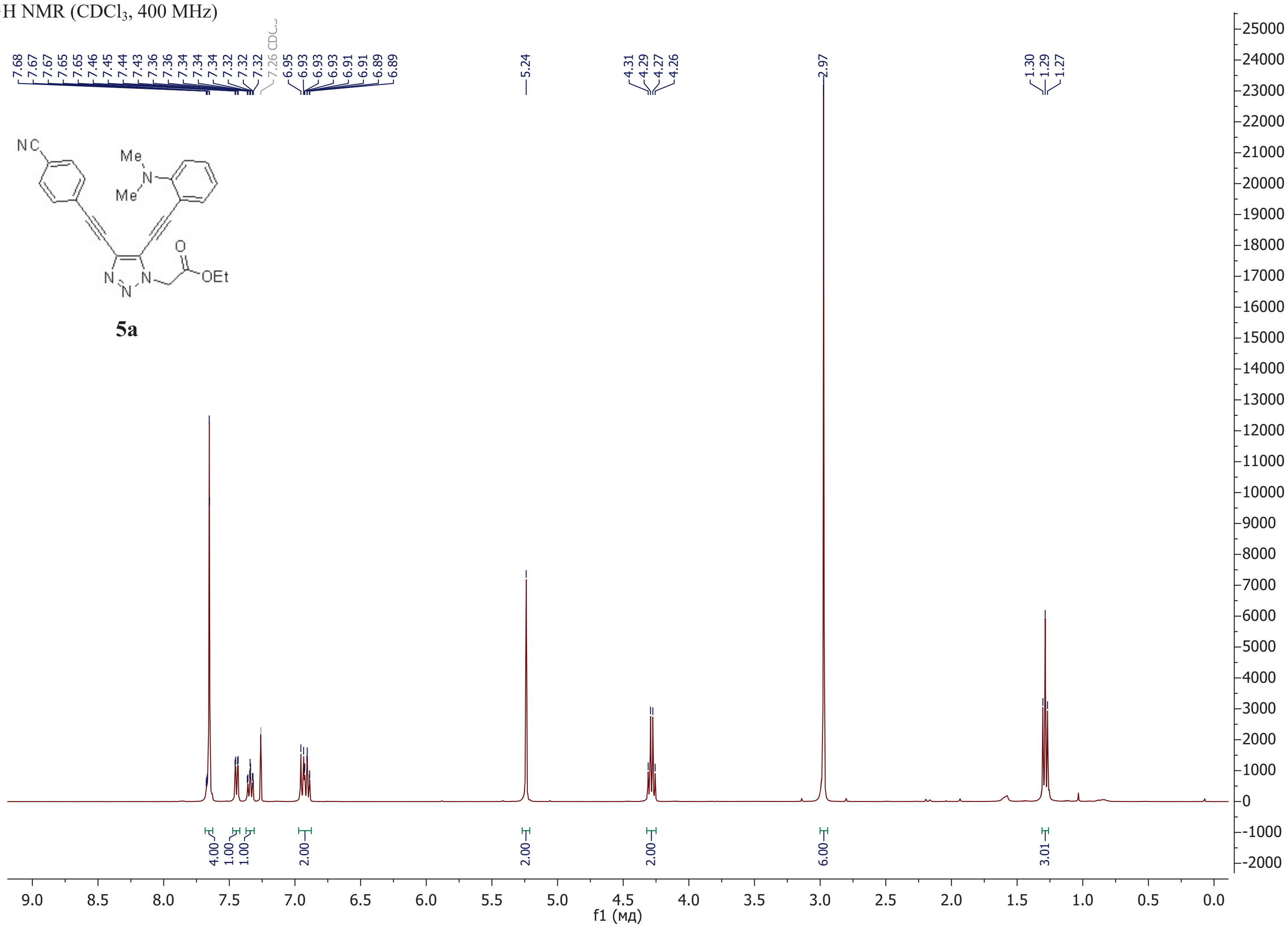

5a

^{13}C NMR ($CDCl_3$, 101 MHz)

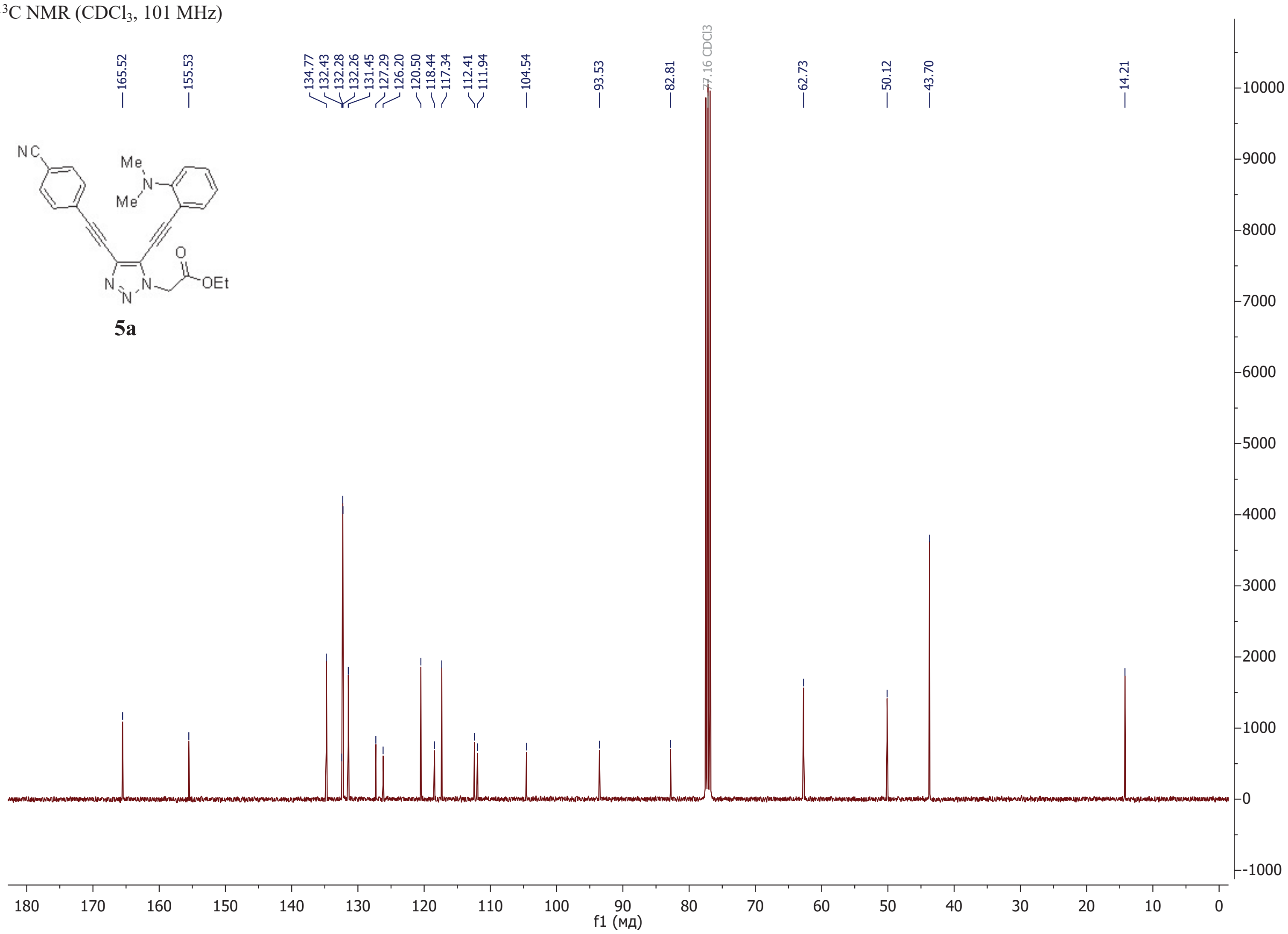

^{13}C NMR ($CDCl_3$, 101 MHz)

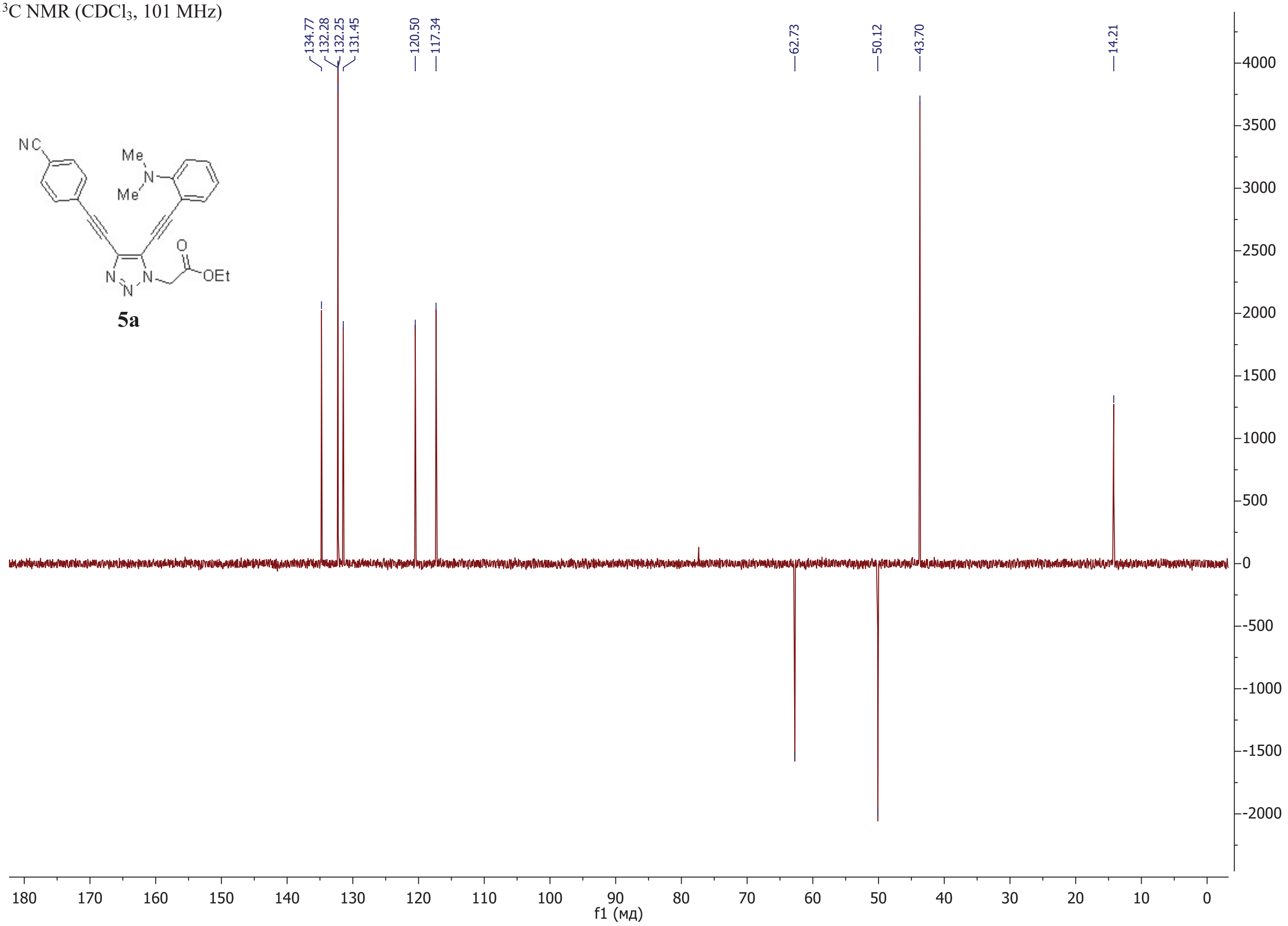

^{1}H NMR ($CDCl_3$, 400 MHz)

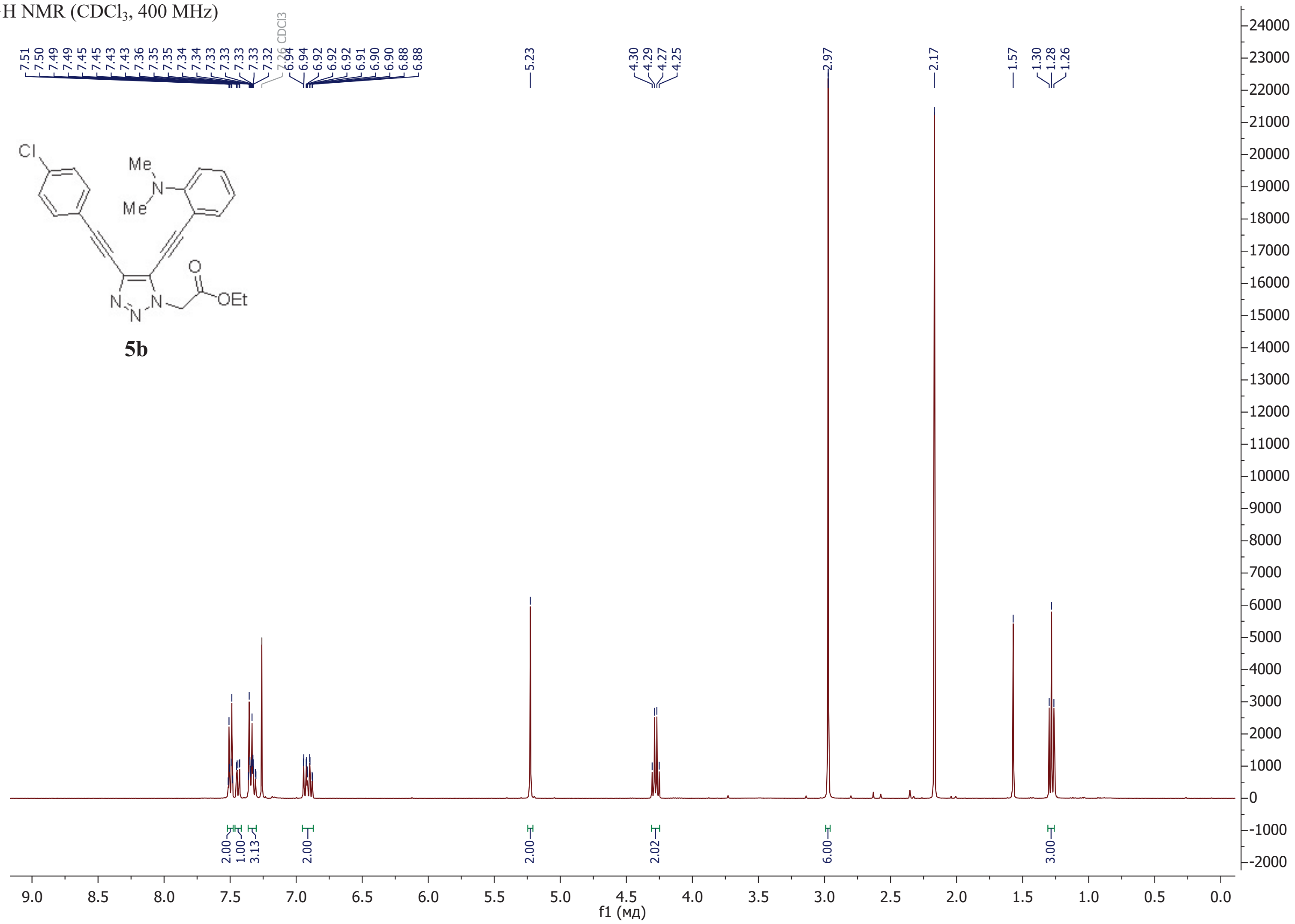

^{13}C NMR ($CDCl_3$, 101 MHz)

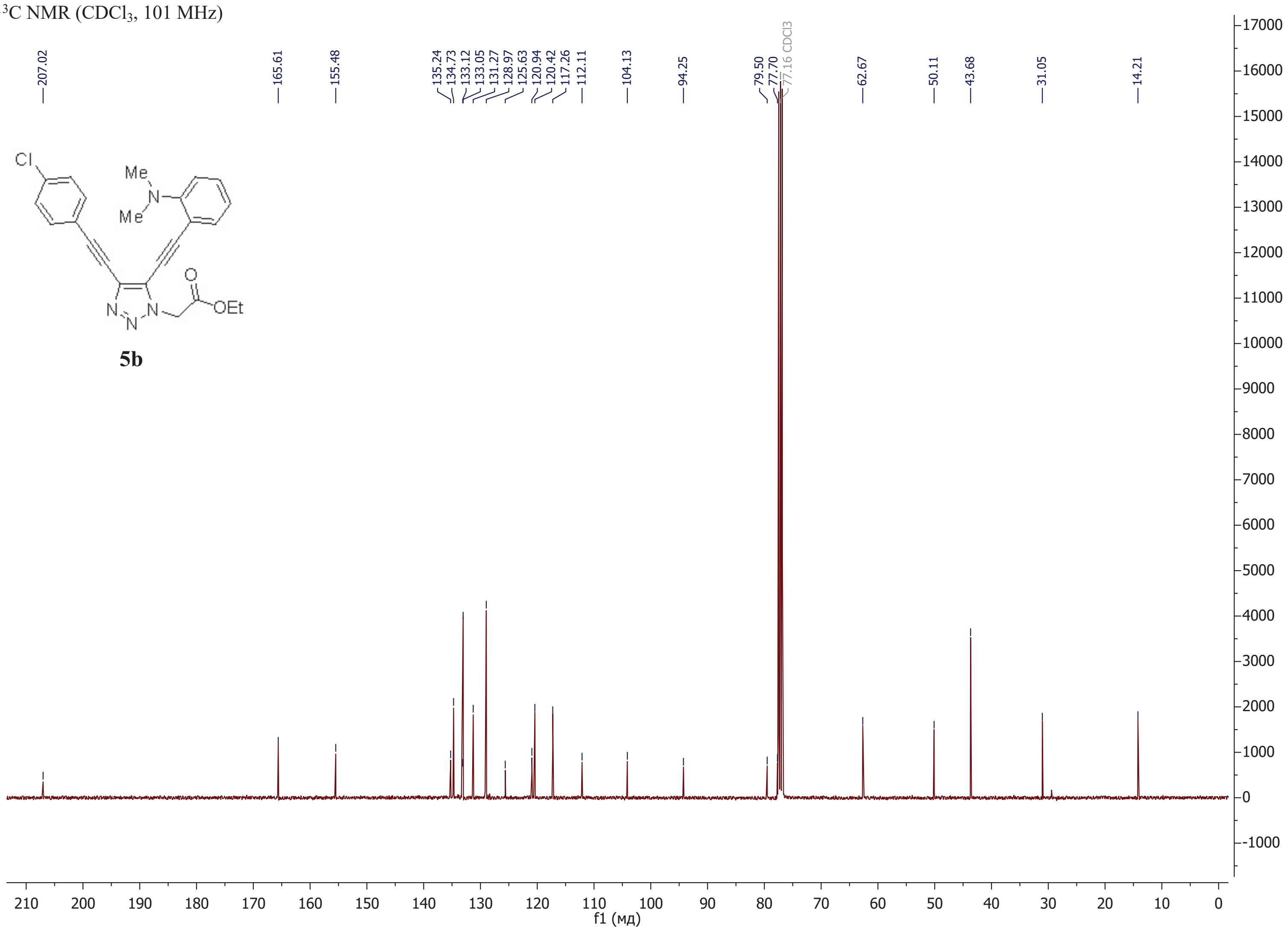

5b

^{13}C NMR ($CDCl_3$, 101 MHz)

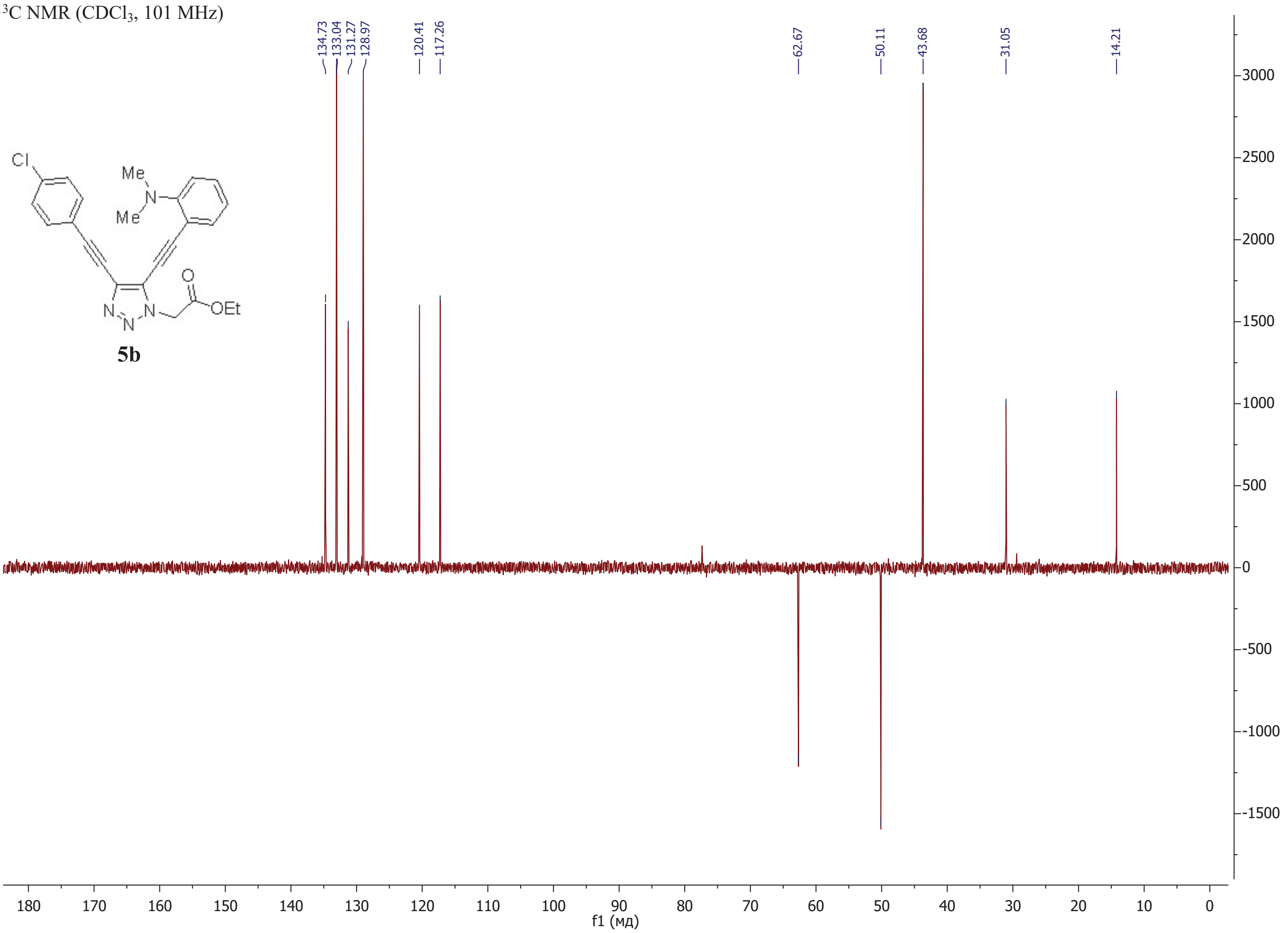

^{1}H NMR ($CDCl_3$, 400 MHz)

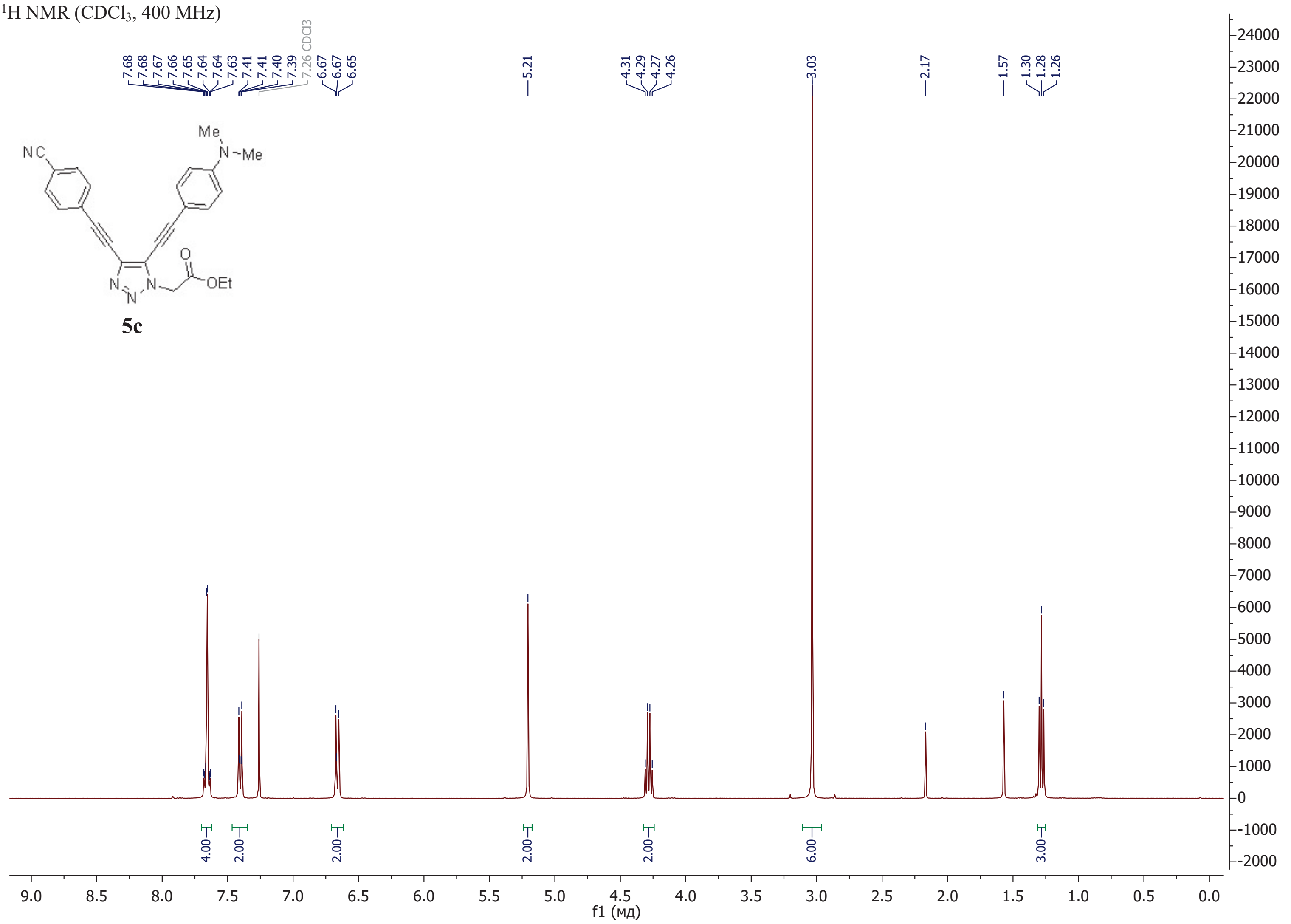

^{13}C NMR ($CDCl_3$, 101 MHz)

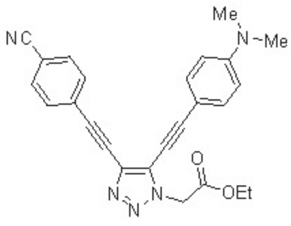

5c

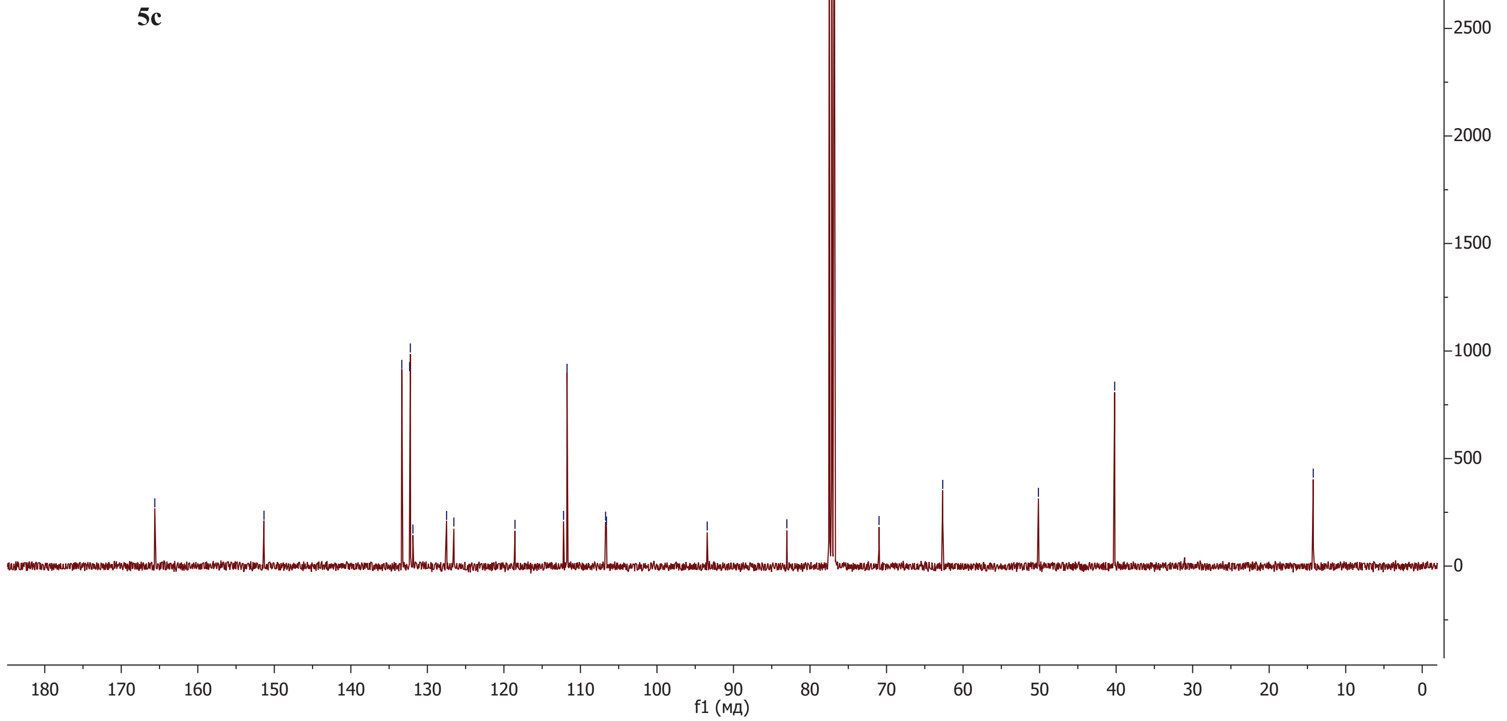

^{13}C NMR ($CDCl_3$, 101 MHz)

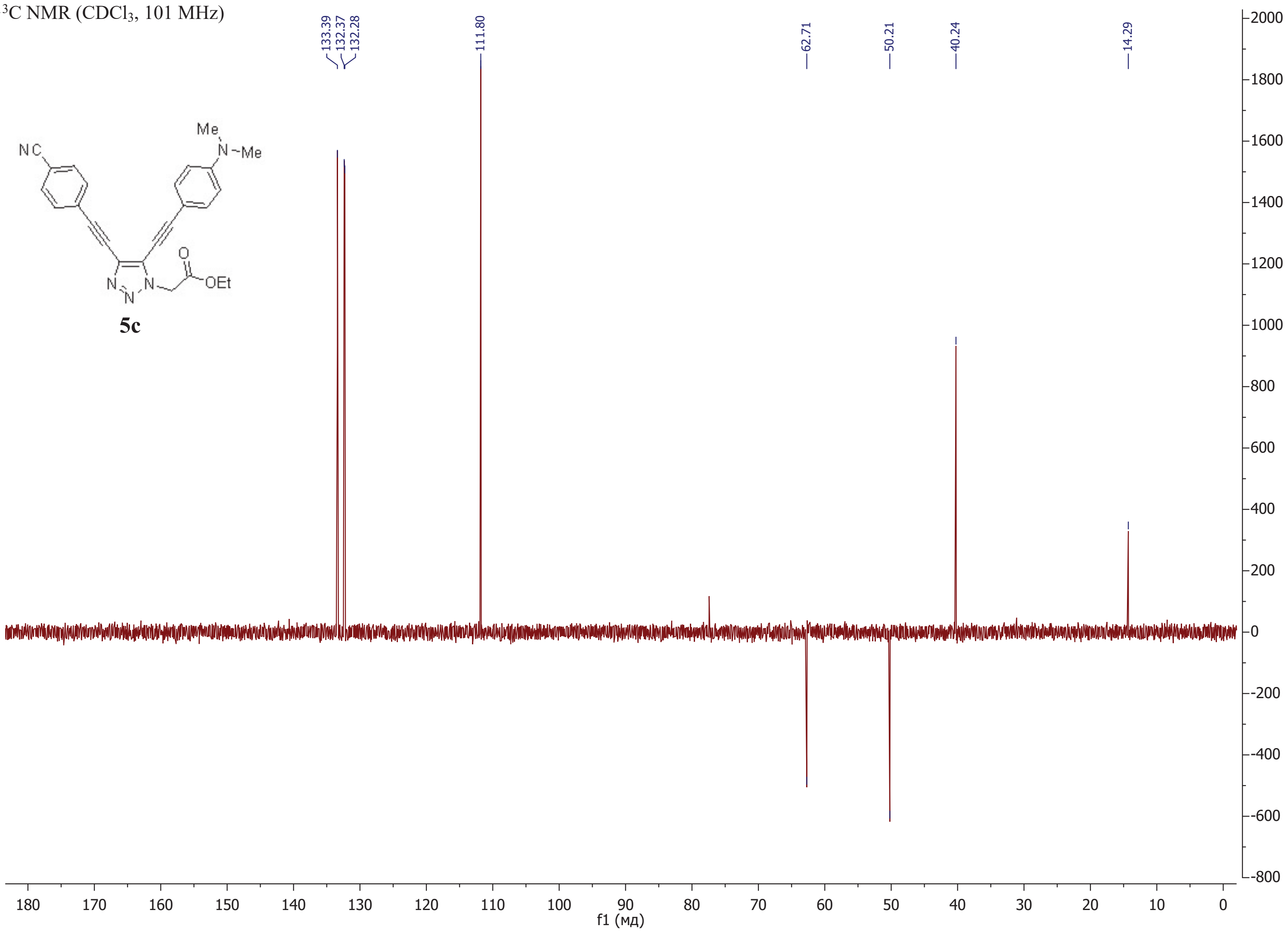

^{1}H NMR (CDCl$_3$, 400 MHz)

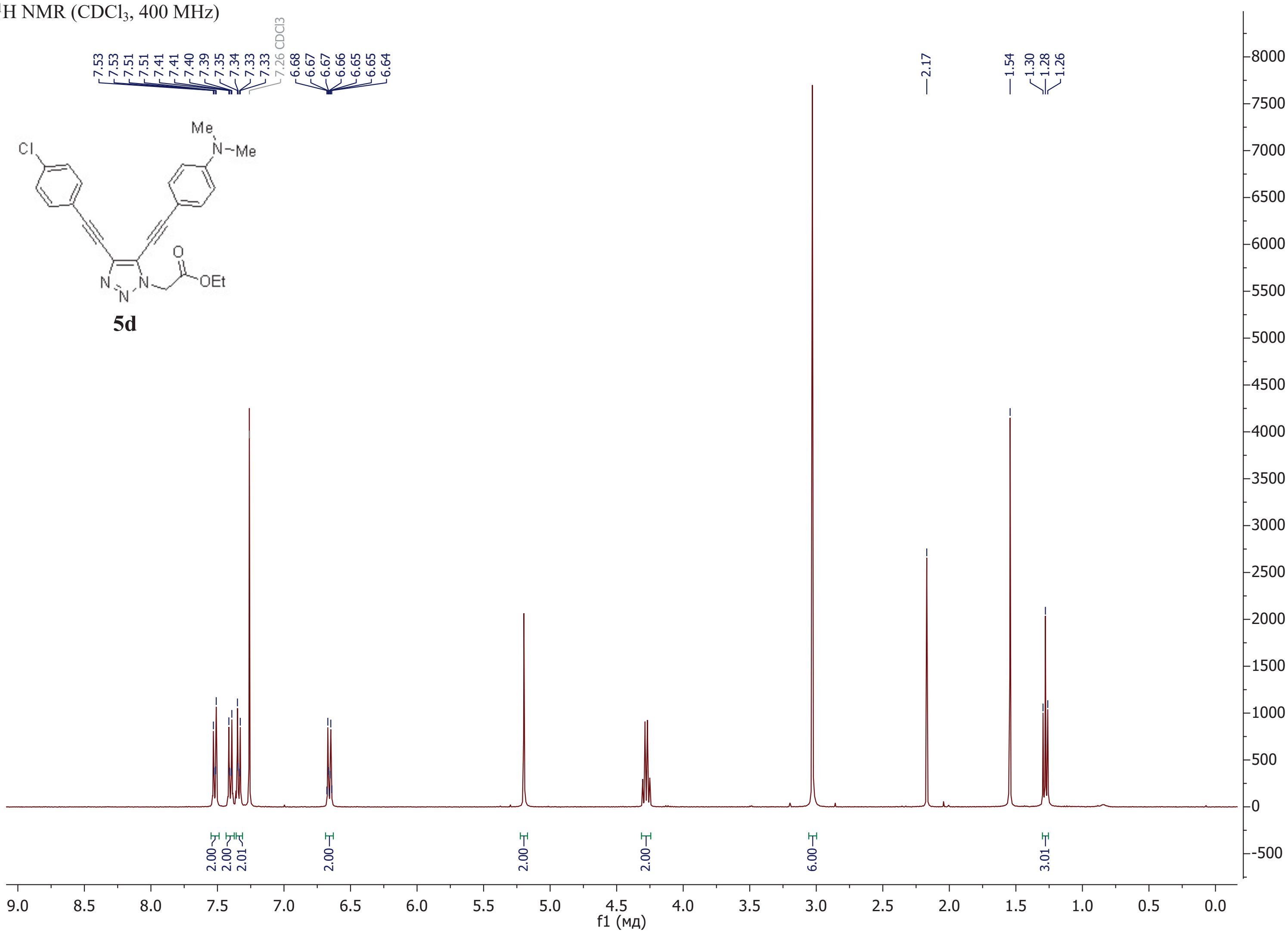

^{13}C NMR ($CDCl_3$, 101 MHz)

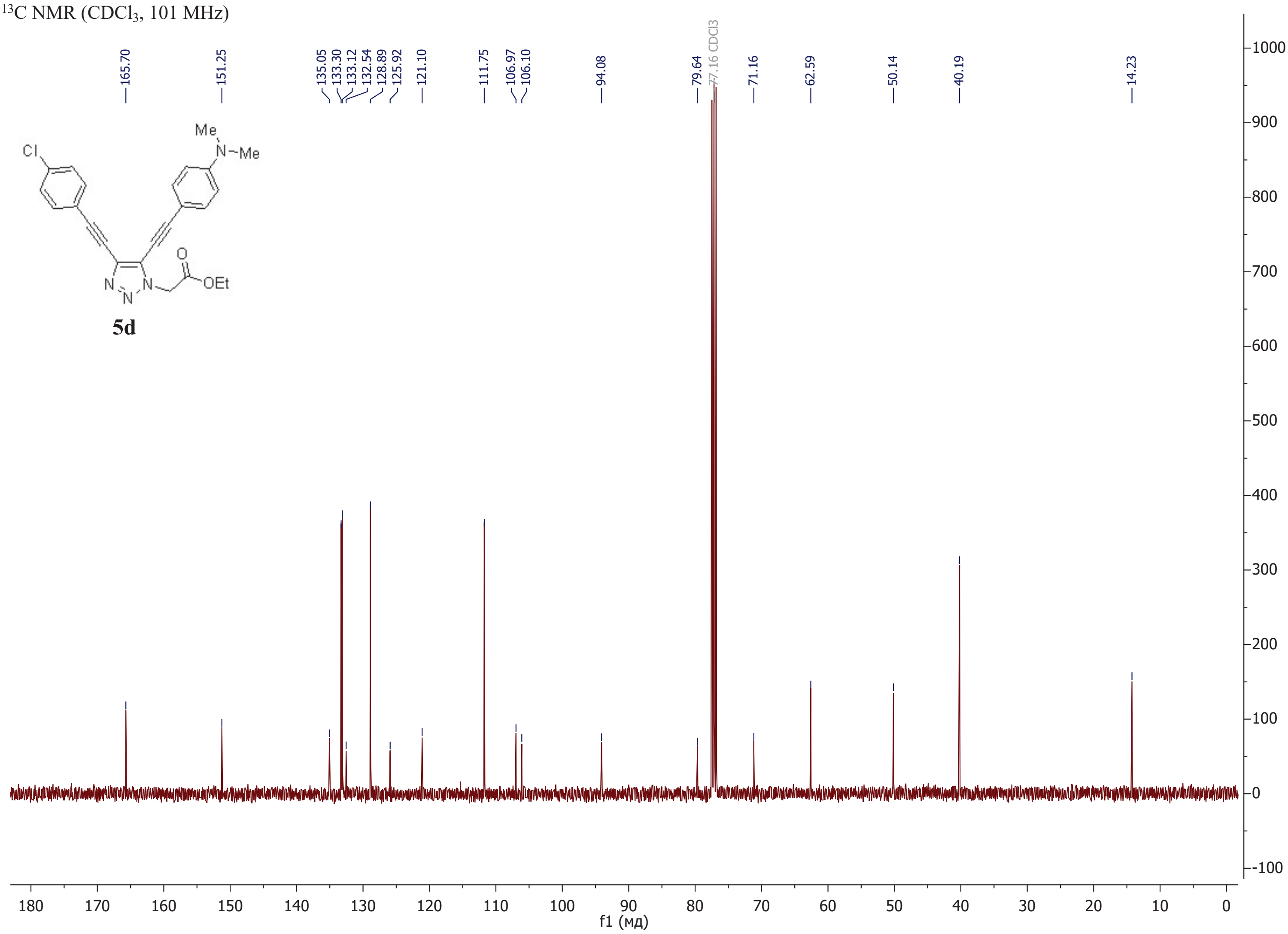

^{13}C NMR ($CDCl_3$, 101 MHz)

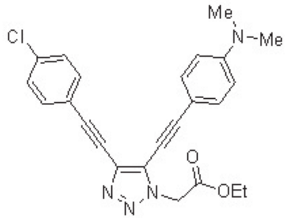

5d

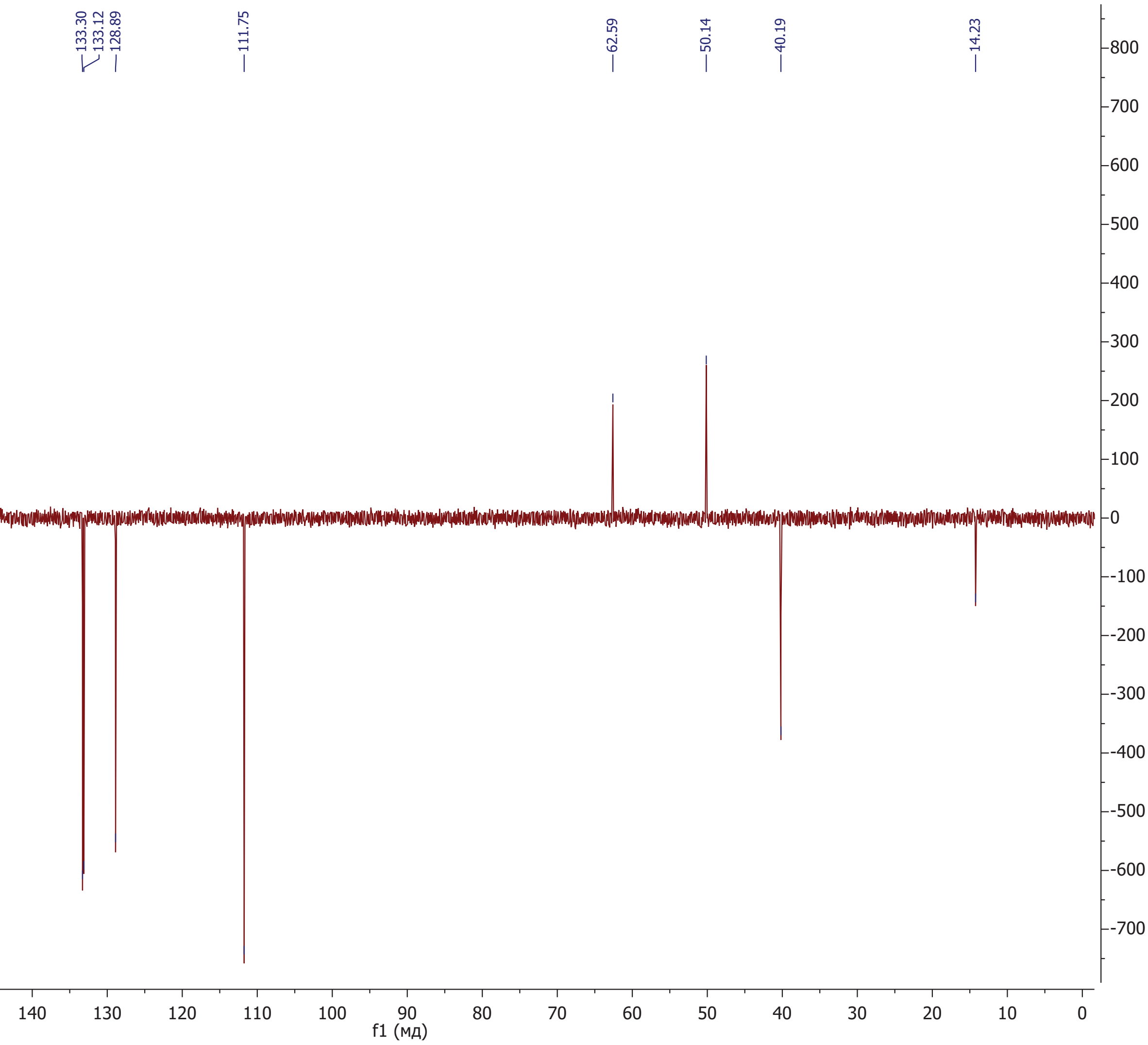

^{1}H NMR ($CDCl_3$, 400 MHz)

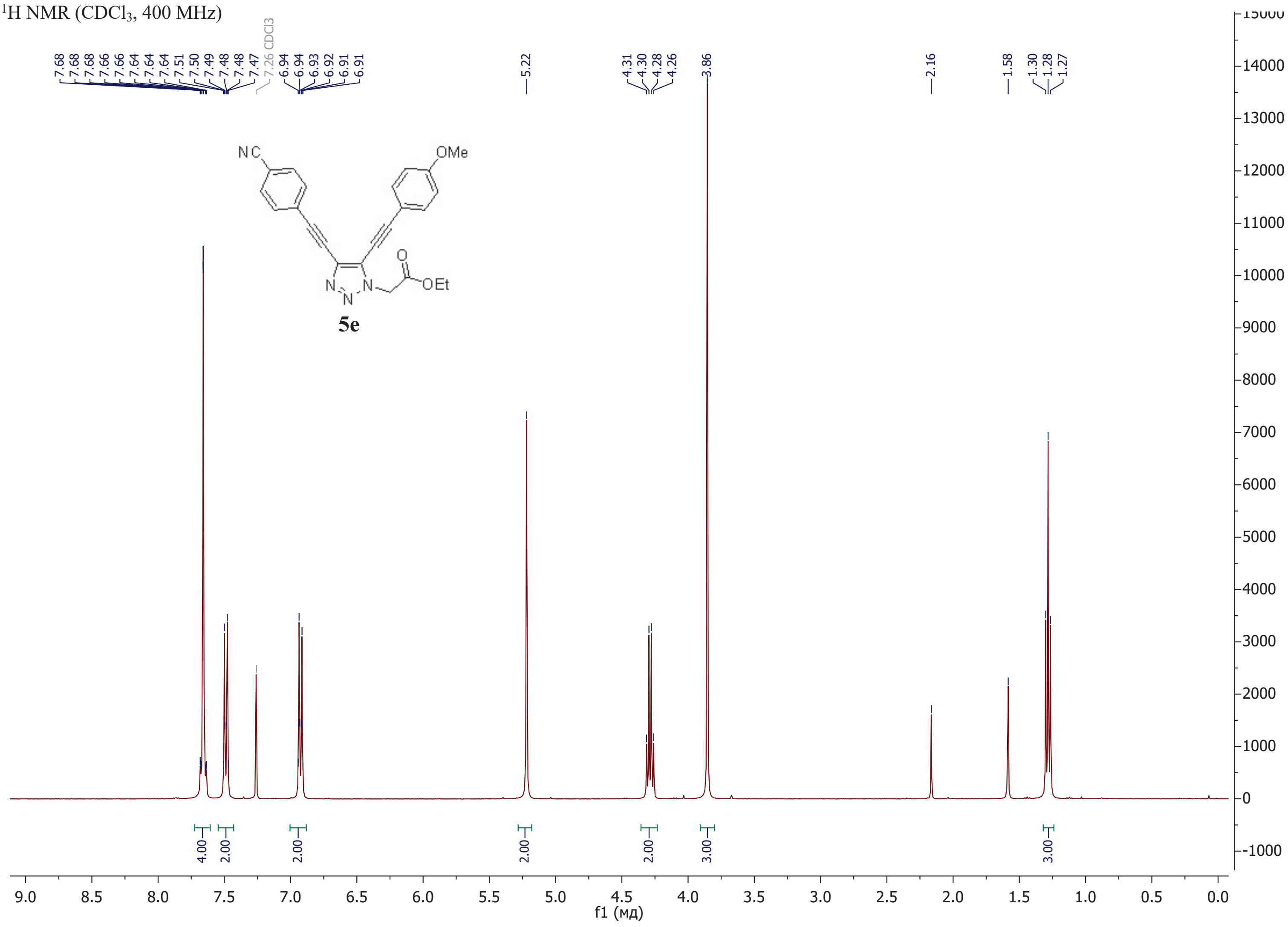

^{13}C NMR ($CDCl_3$, 101 MHz)

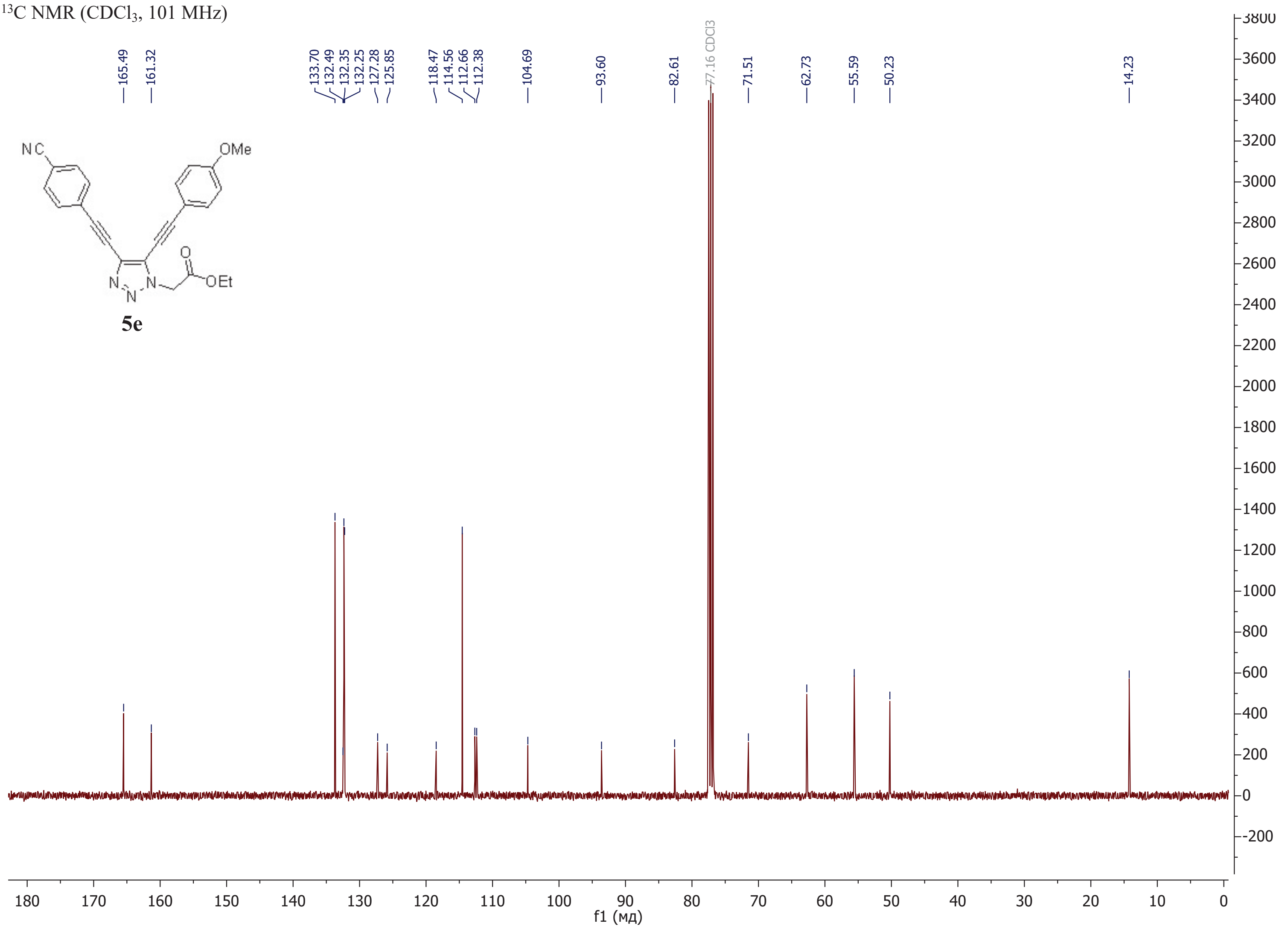

^{13}C NMR ($CDCl_3$, 101 MHz)

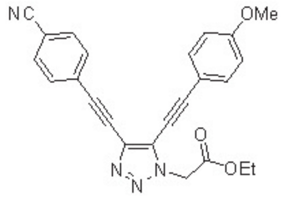

5e

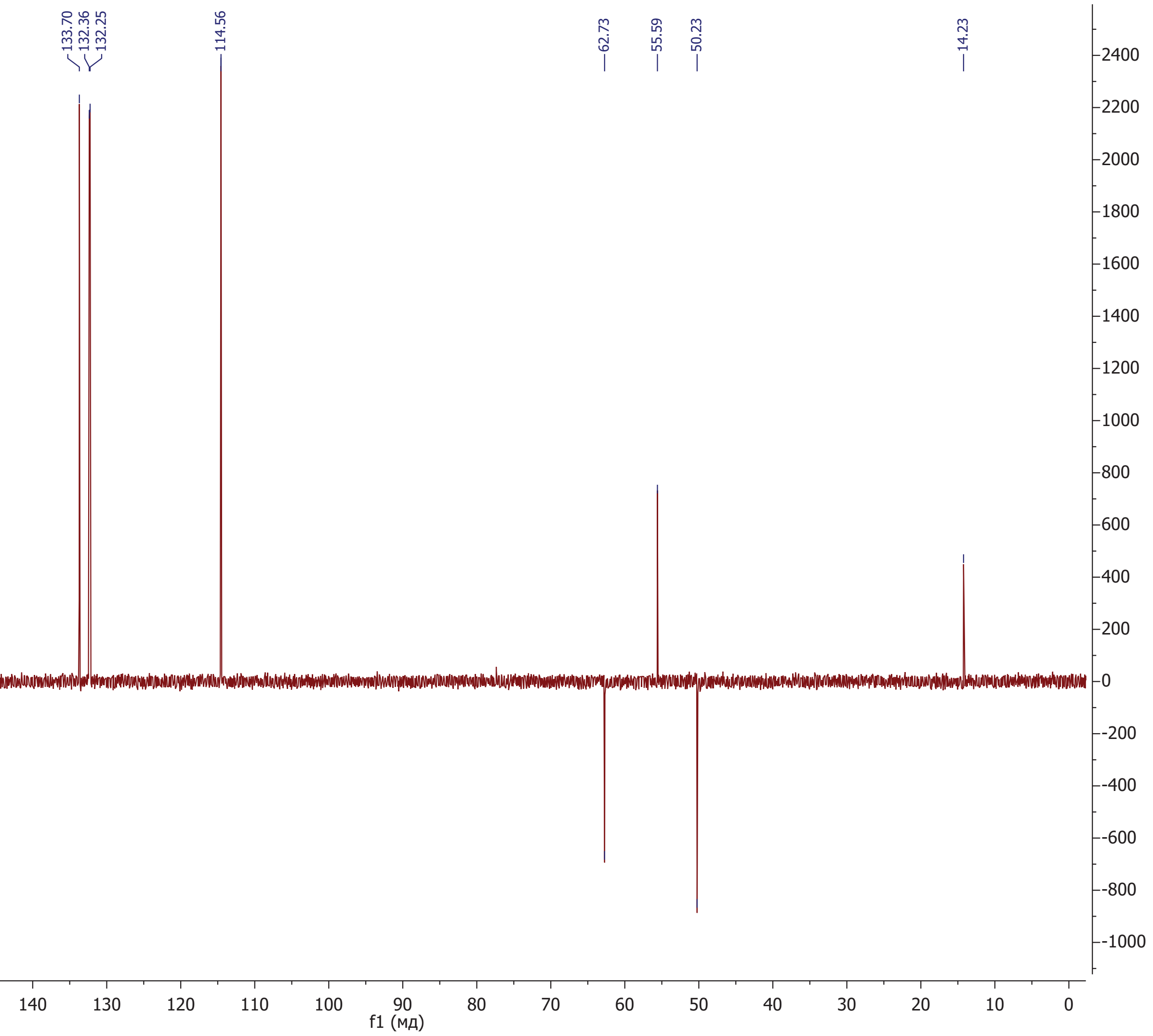

^{1}H NMR (CDCl$_3$, 400 MHz)

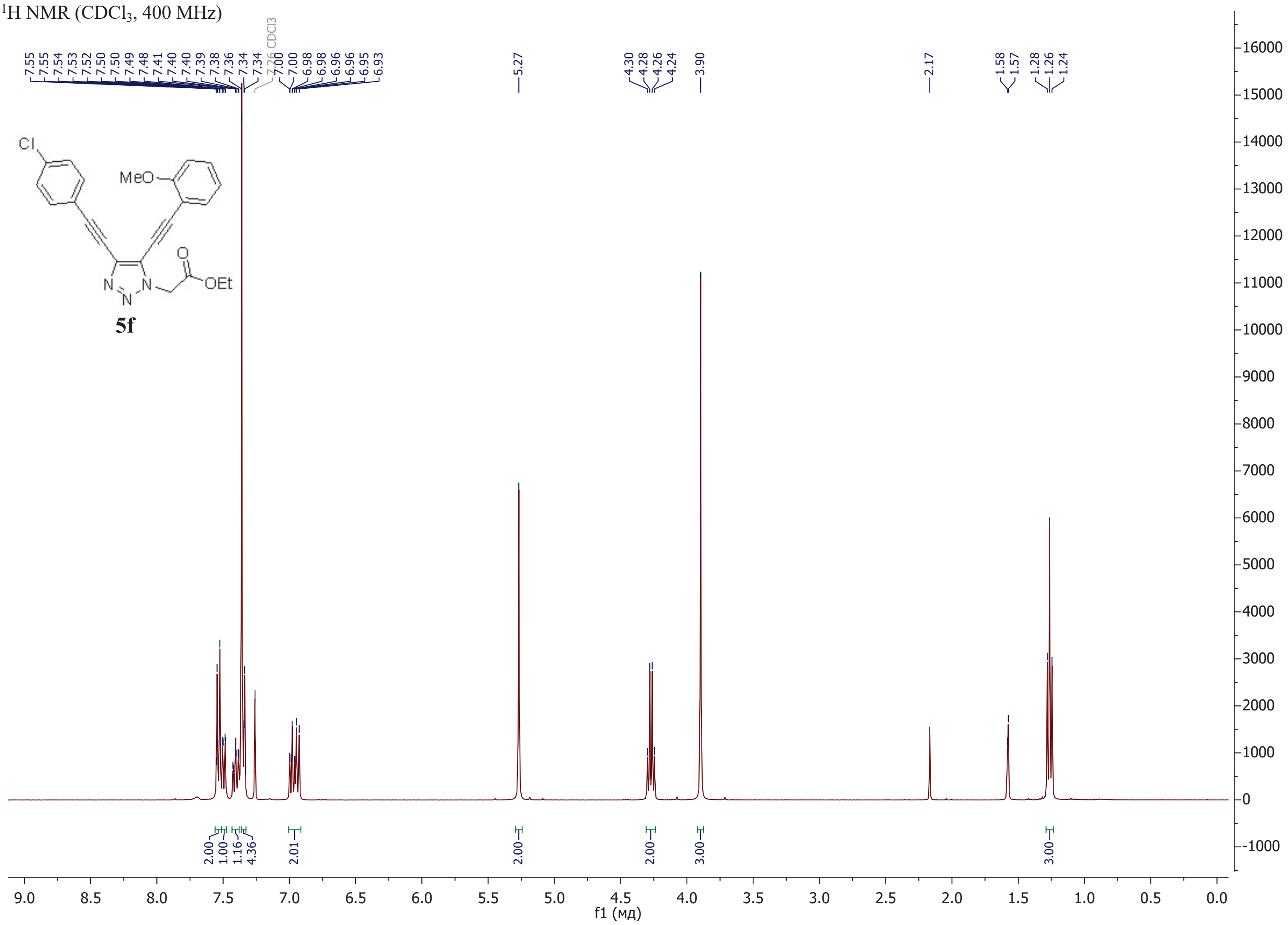

^{13}C NMR ($CDCl_3$, 101 MHz)

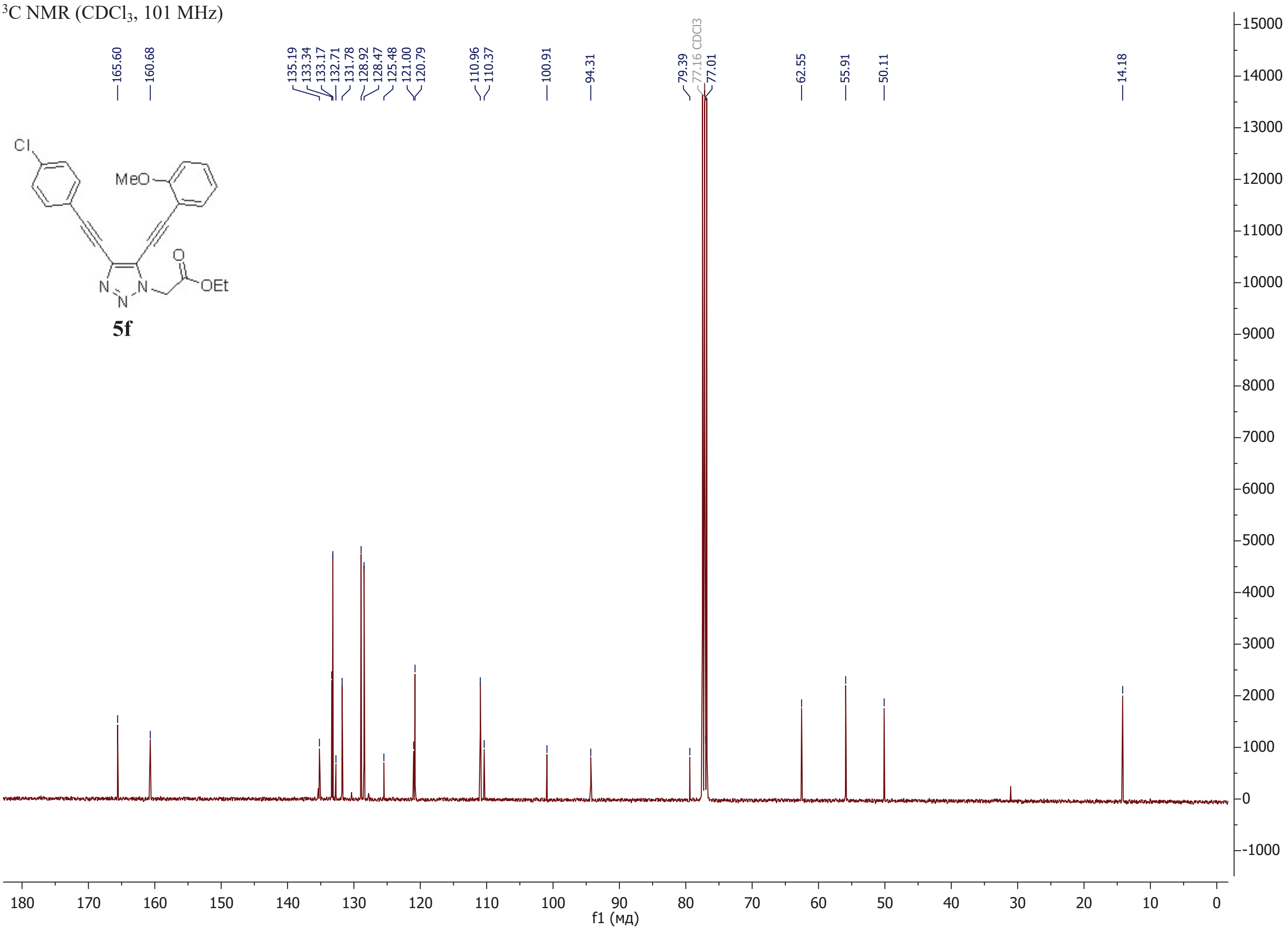

^{13}C NMR ($CDCl_3$, 101 MHz)

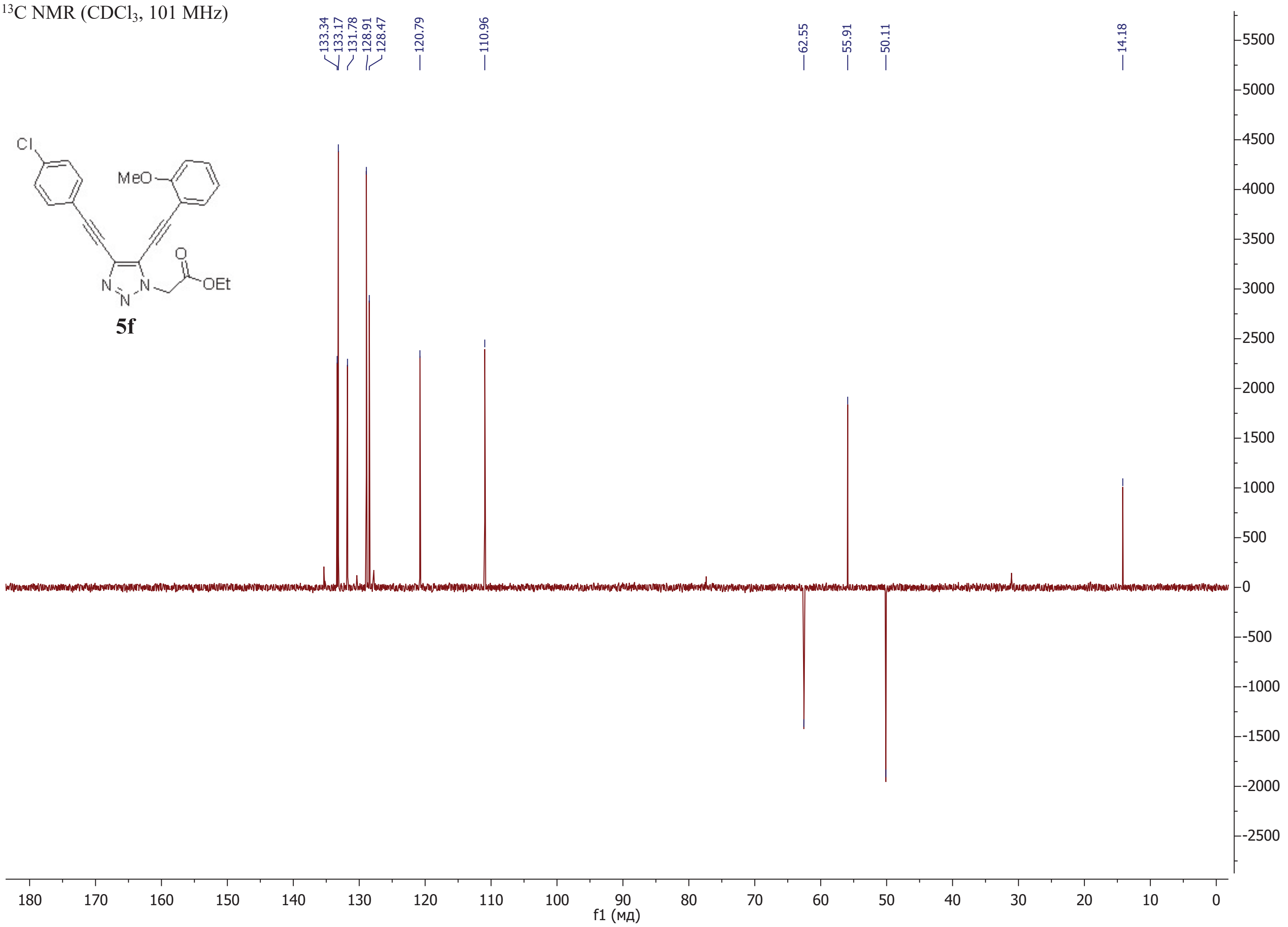

5f

^{1}H NMR ($CDCl_3$, 400 MHz)

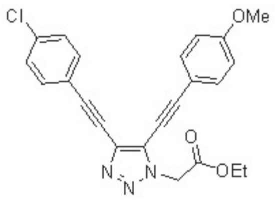

5g

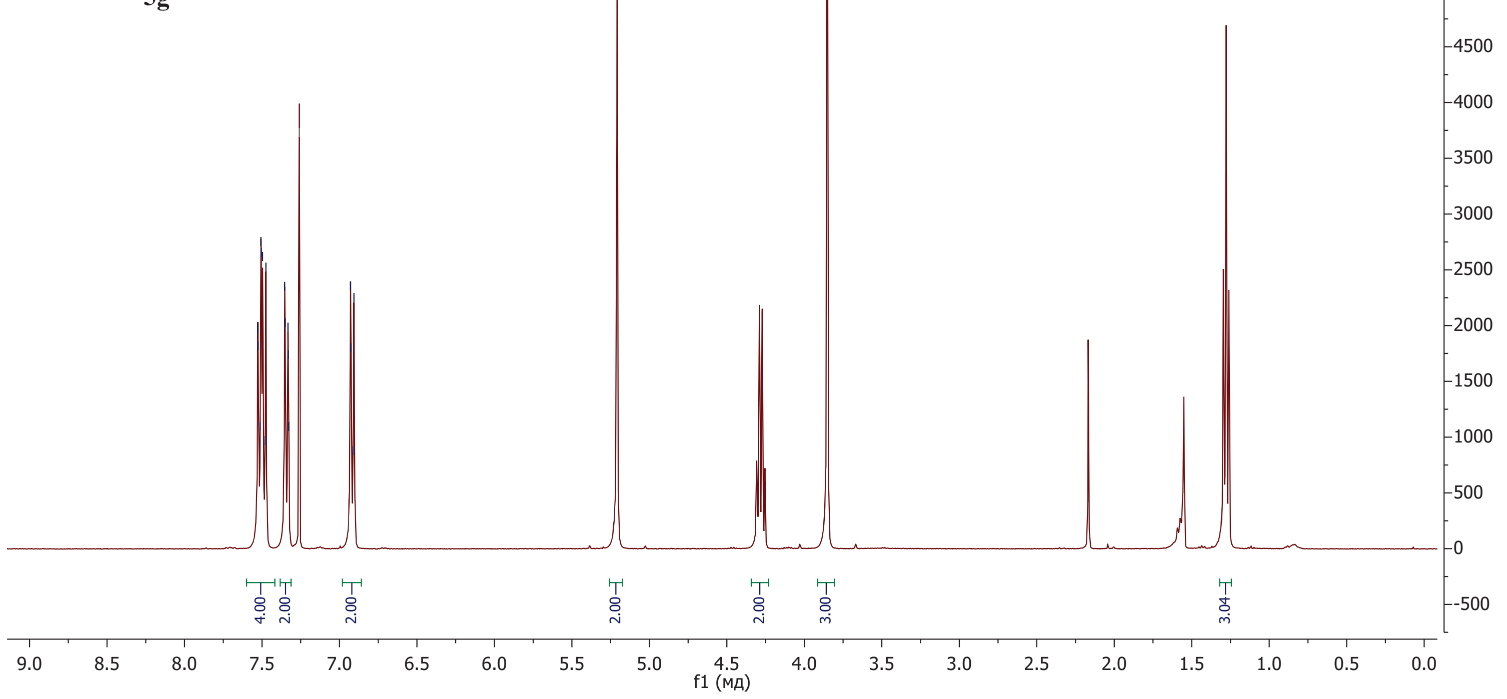

^{13}C NMR ($CDCl_3$, 101 MHz)

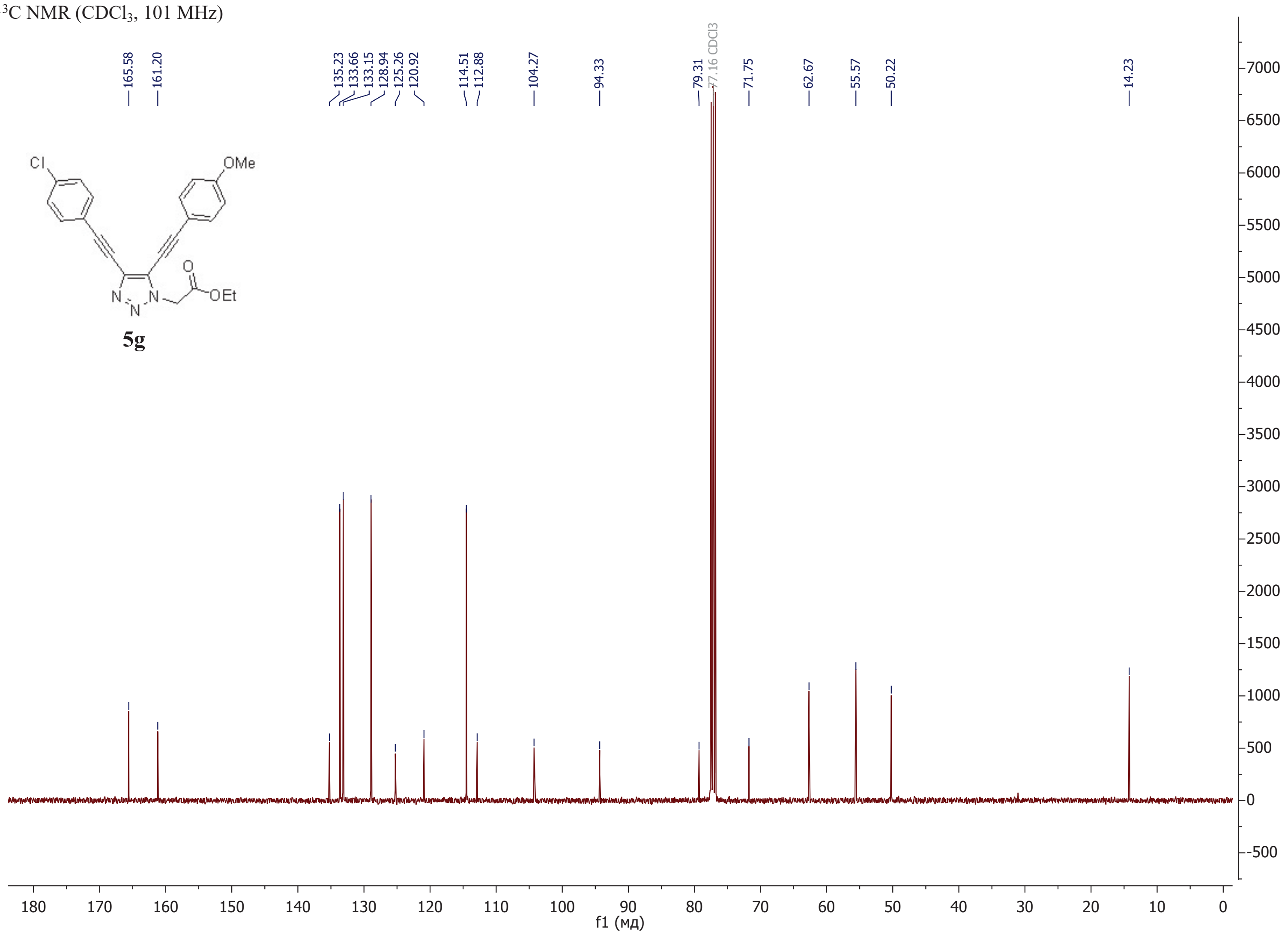

^{13}C NMR ($CDCl_3$, 101 MHz)

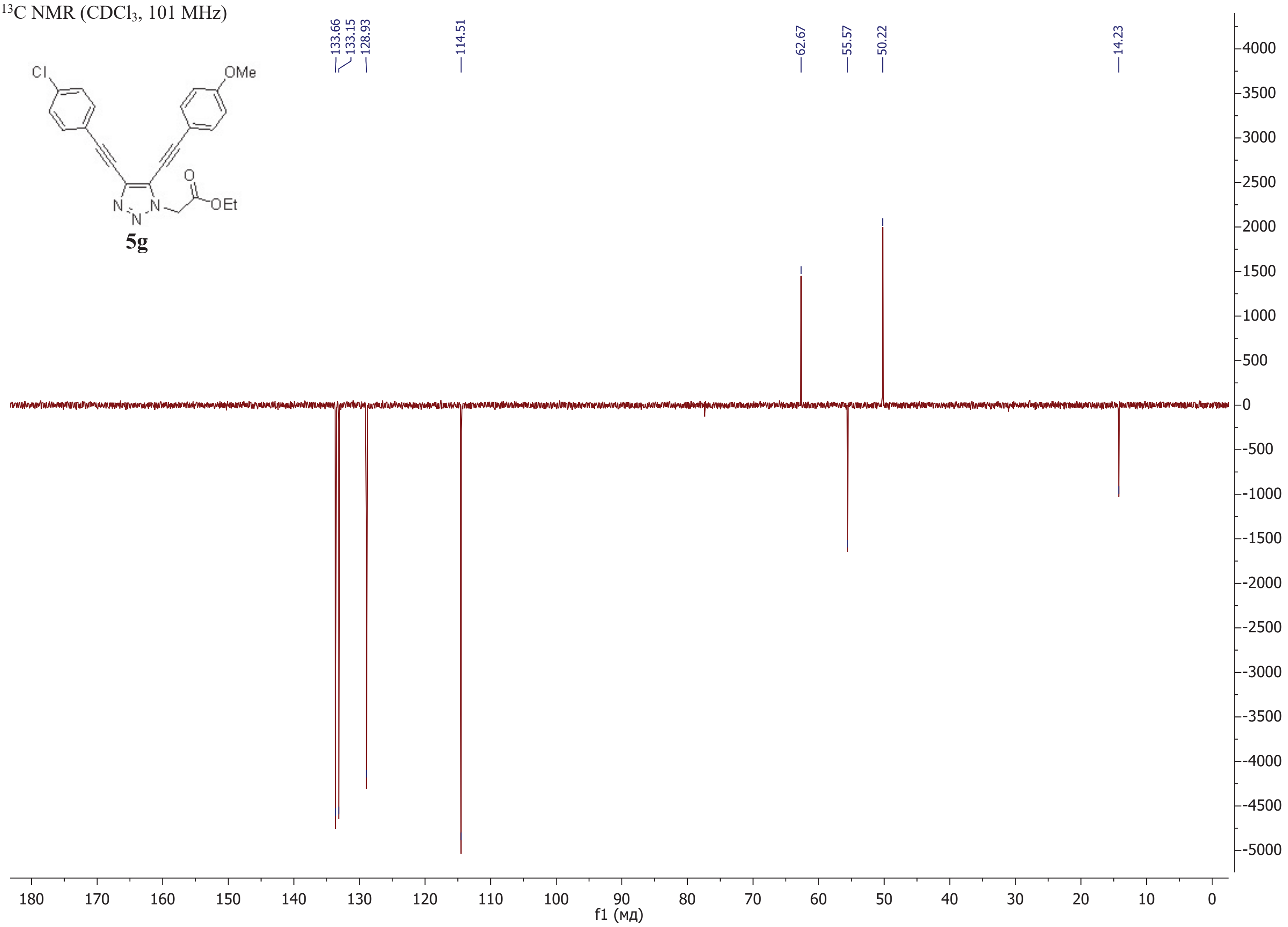

^{1}H NMR ($CDCl_3$, 400 MHz)

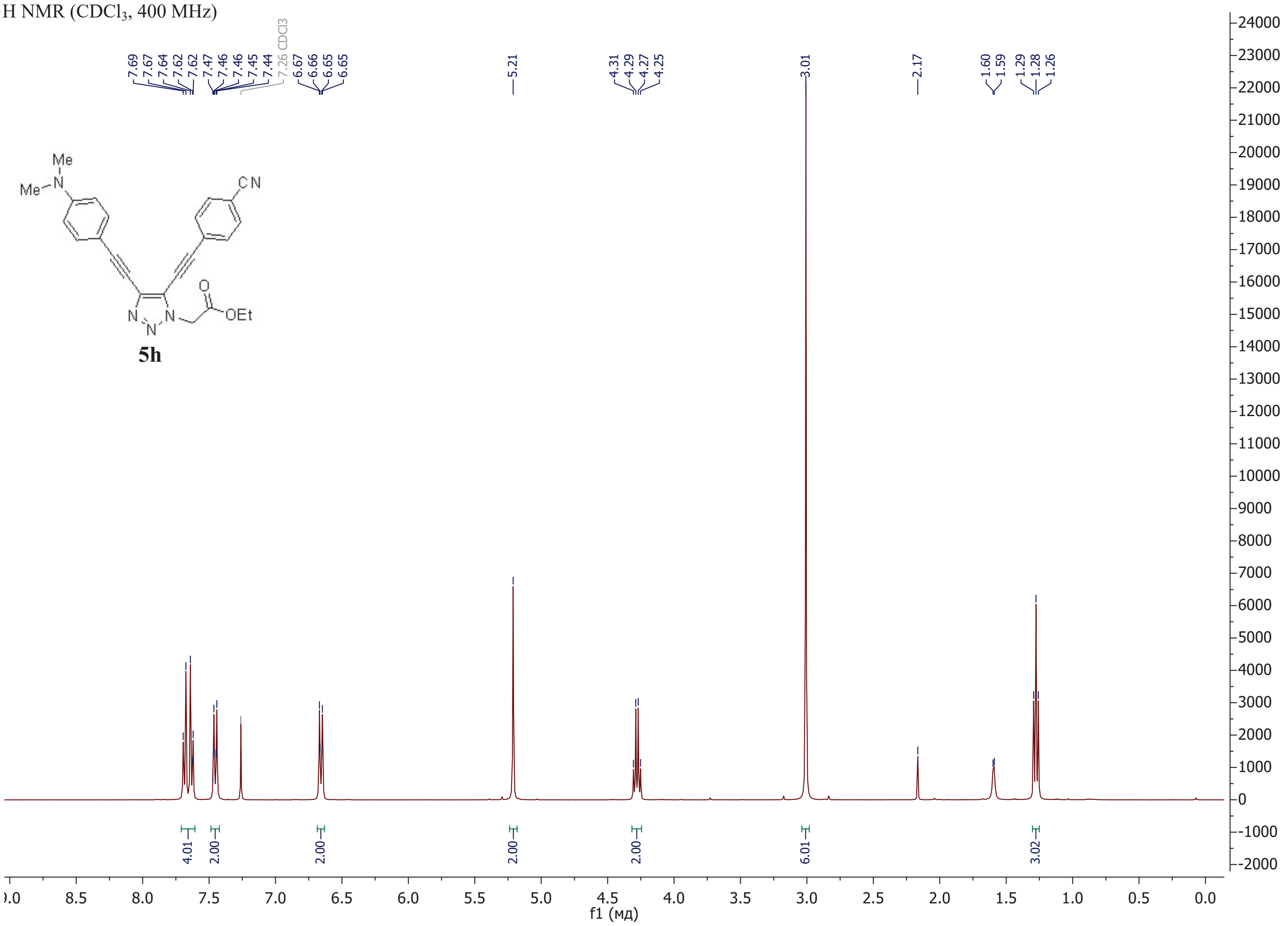

^{13}C NMR ($CDCl_3$, 101 MHz)

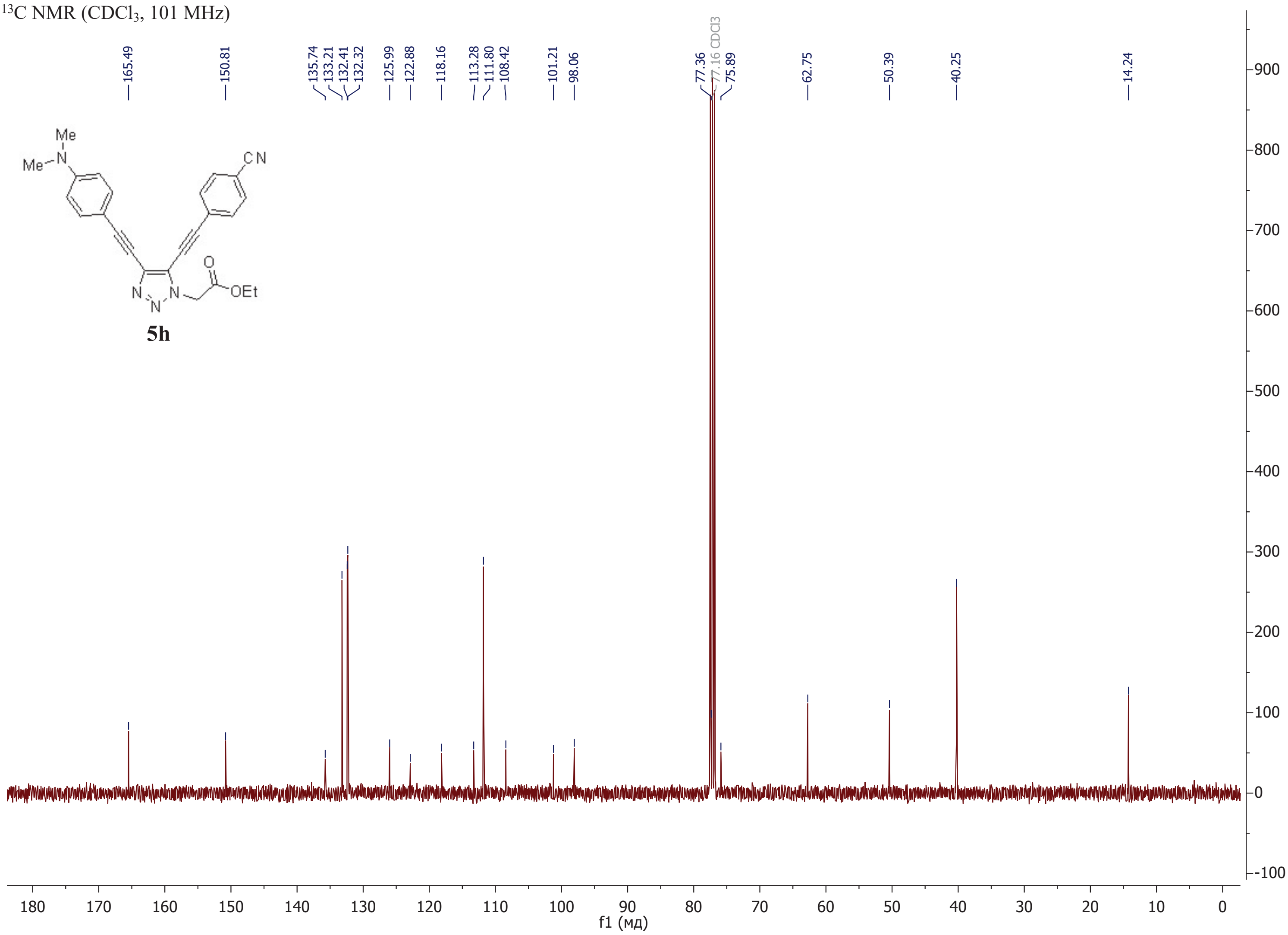

^{13}C NMR ($CDCl_3$, 101 MHz)

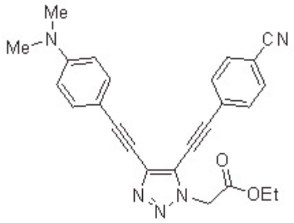

5h

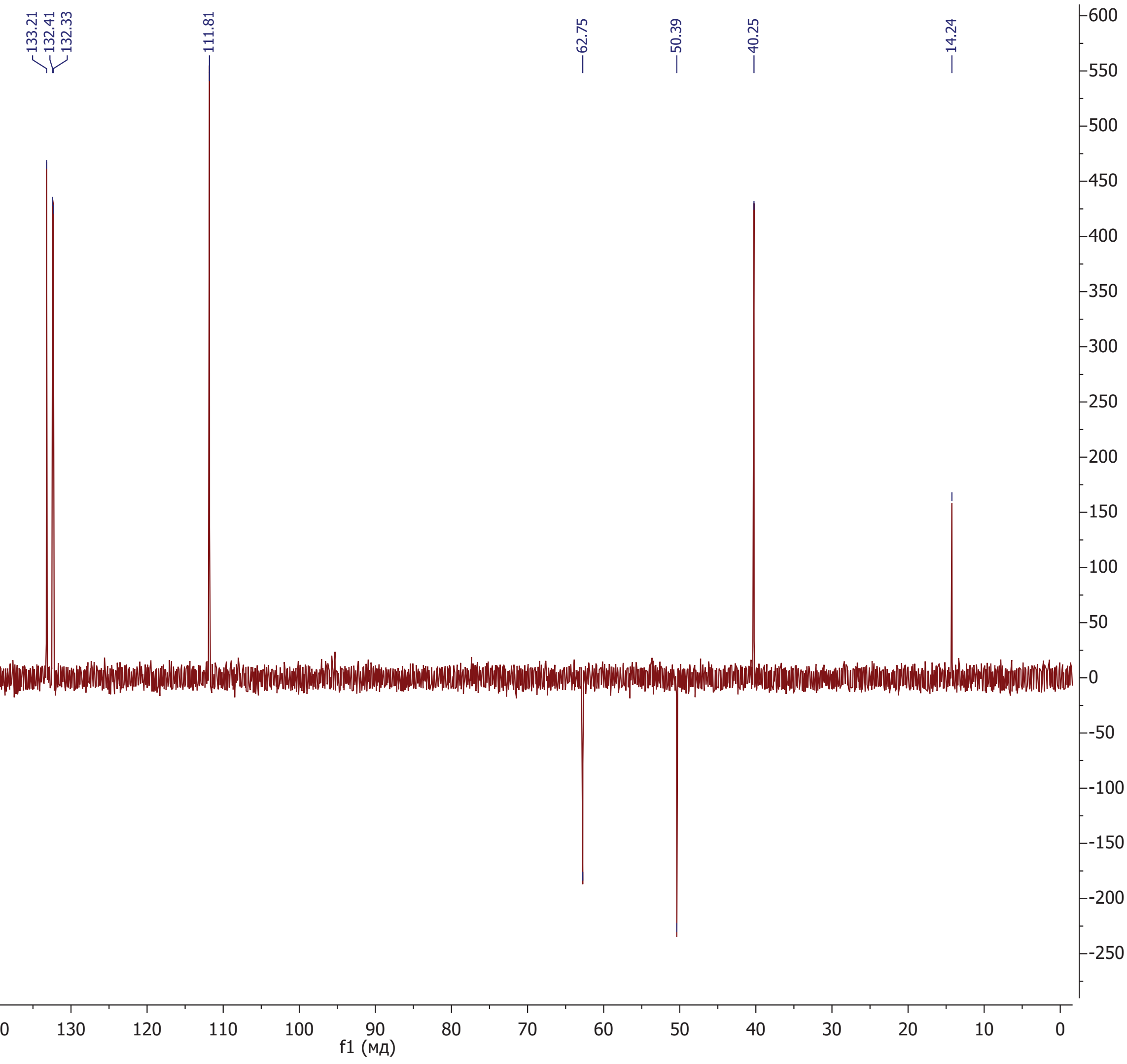

^{1}H NMR ($CDCl_3$, 400 MHz)

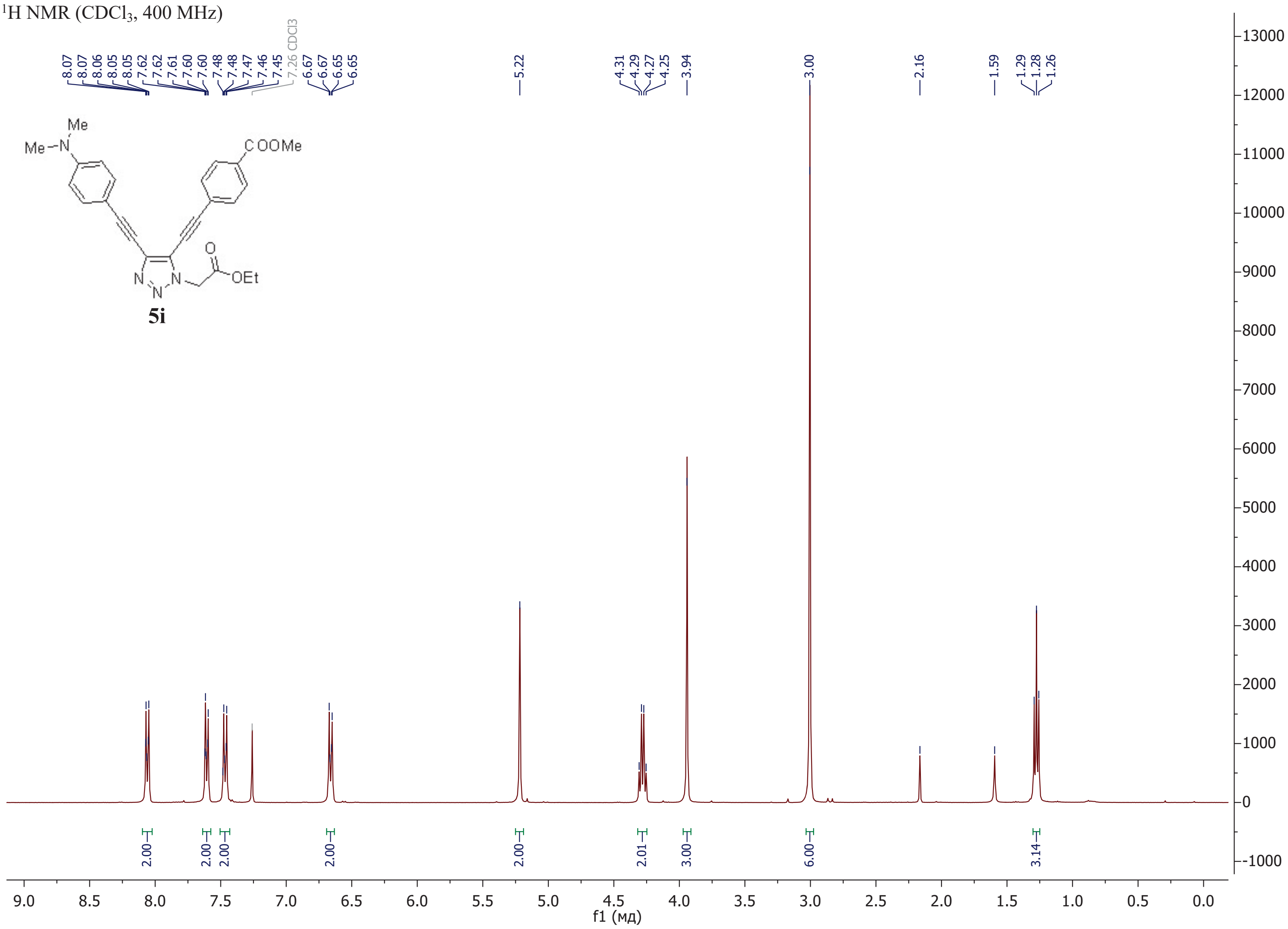

^{13}C NMR ($CDCl_3$, 101 MHz)

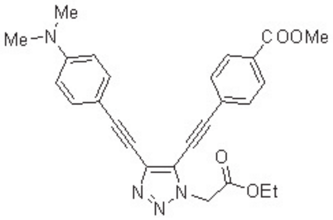

5i

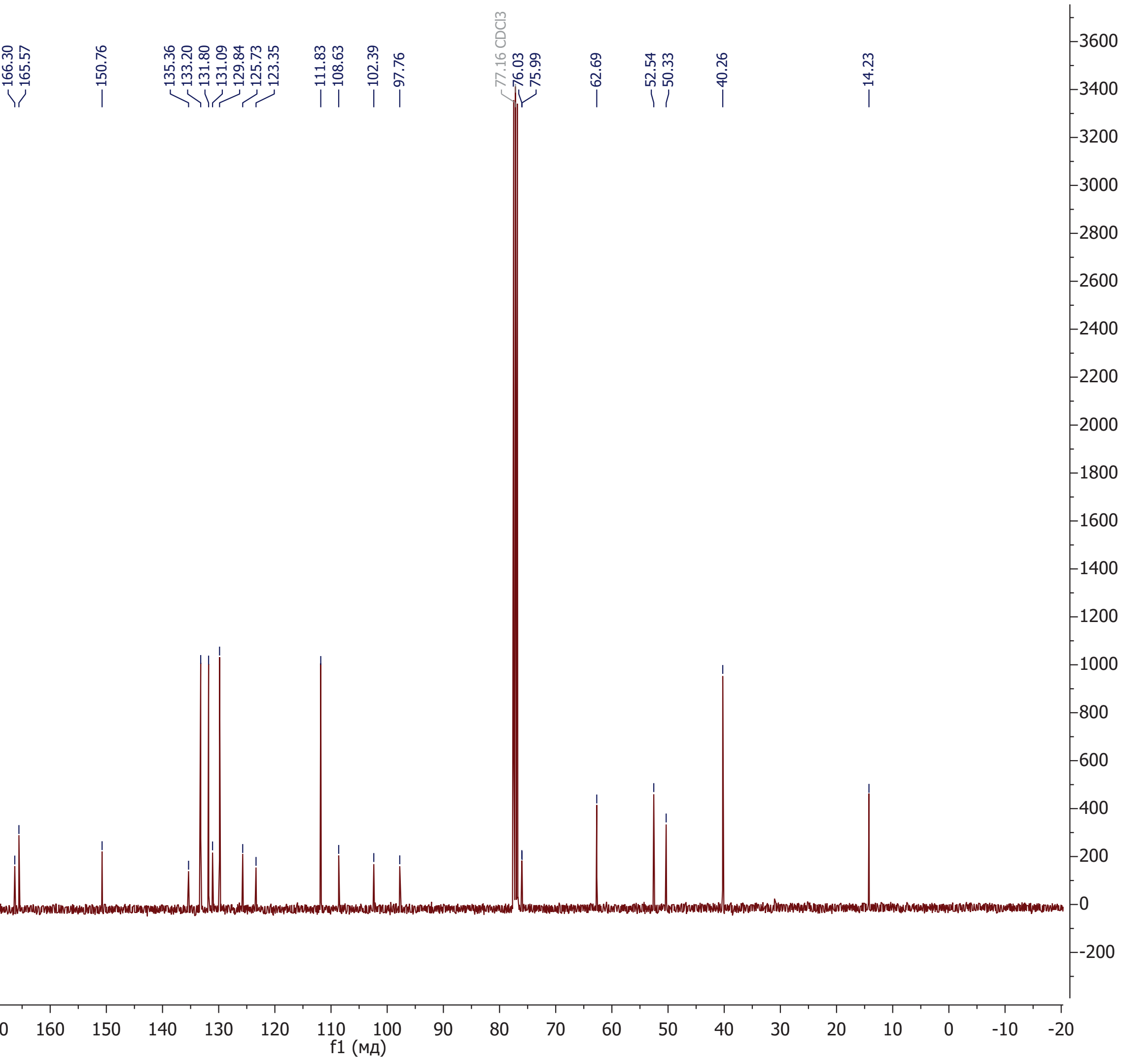

^{13}C NMR ($CDCl_3$, 101 MHz)

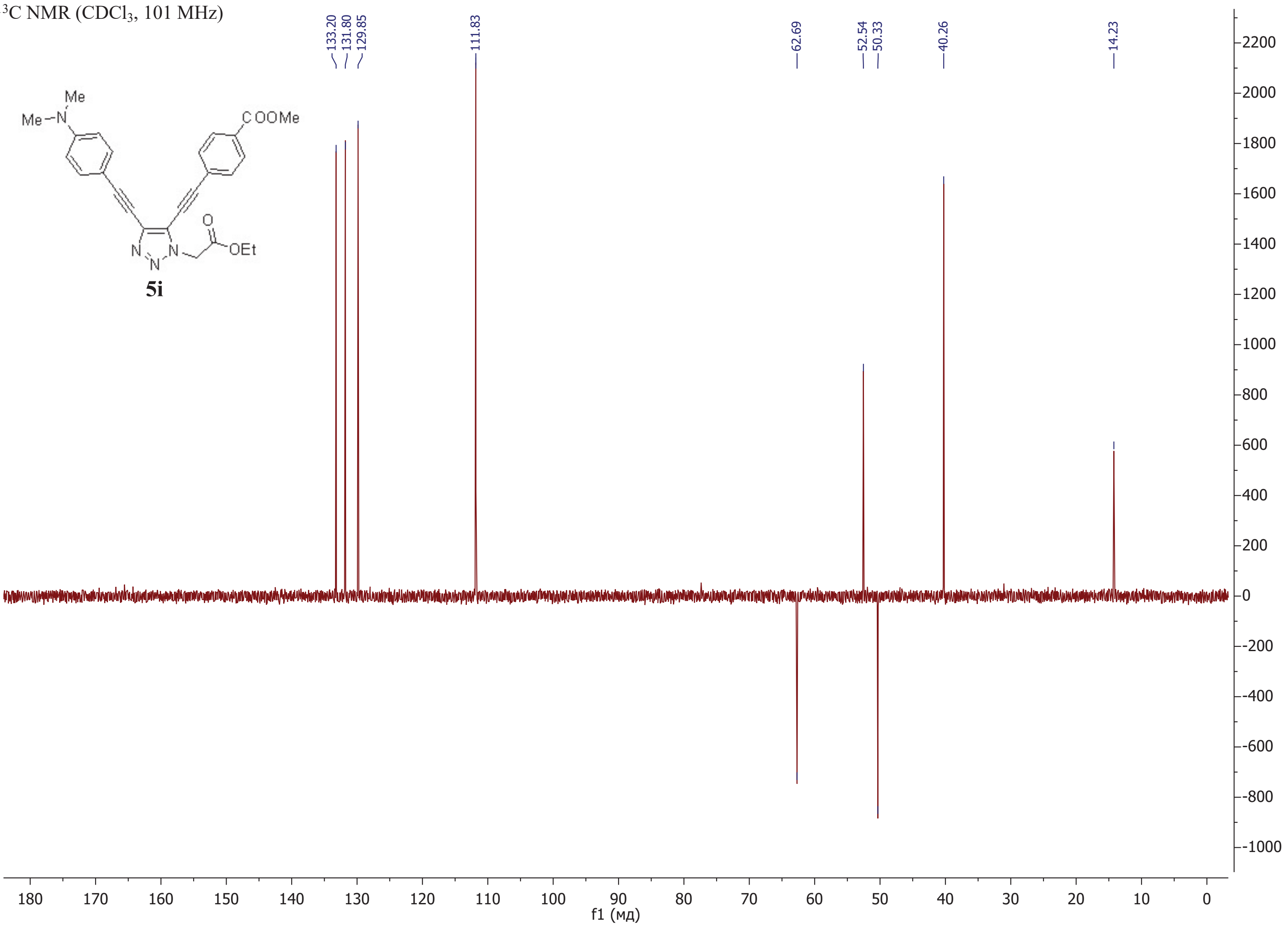

5i

^1H NMR ($CDCl_3$, 400 MHz)

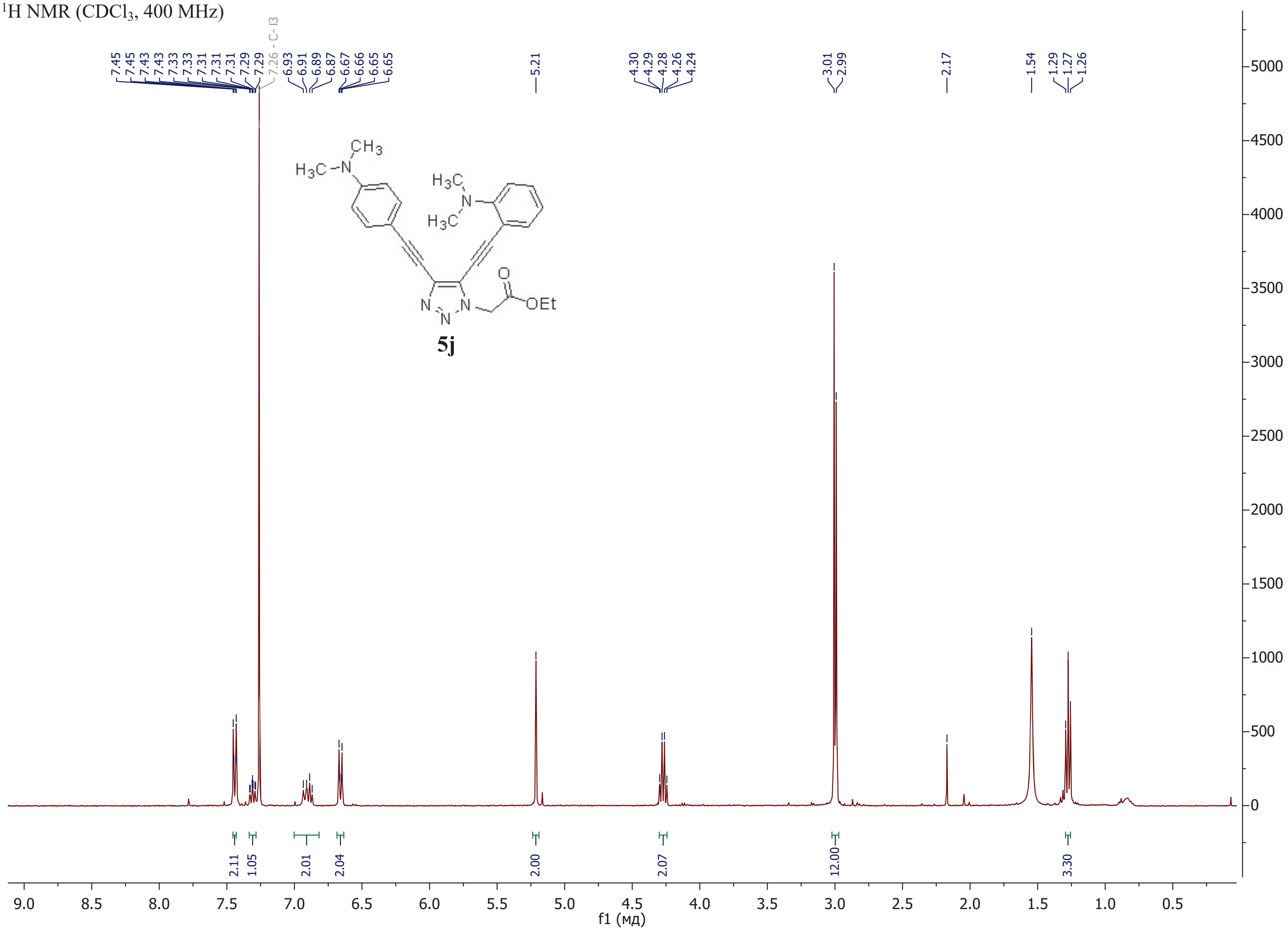

^{13}C NMR ($CDCl_3$, 126 MHz)

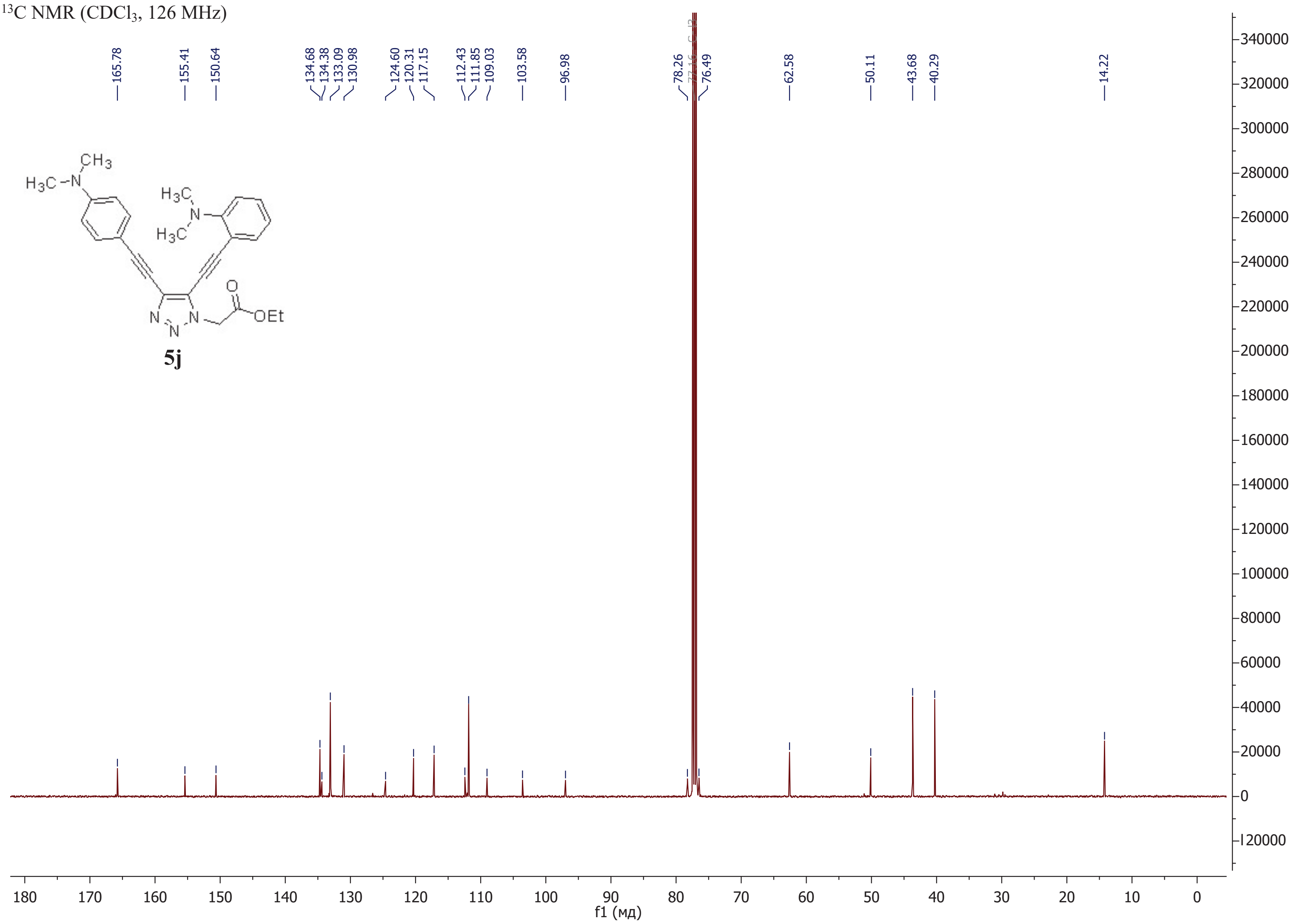

^{13}C NMR ($CDCl_3$, 126 MHz)

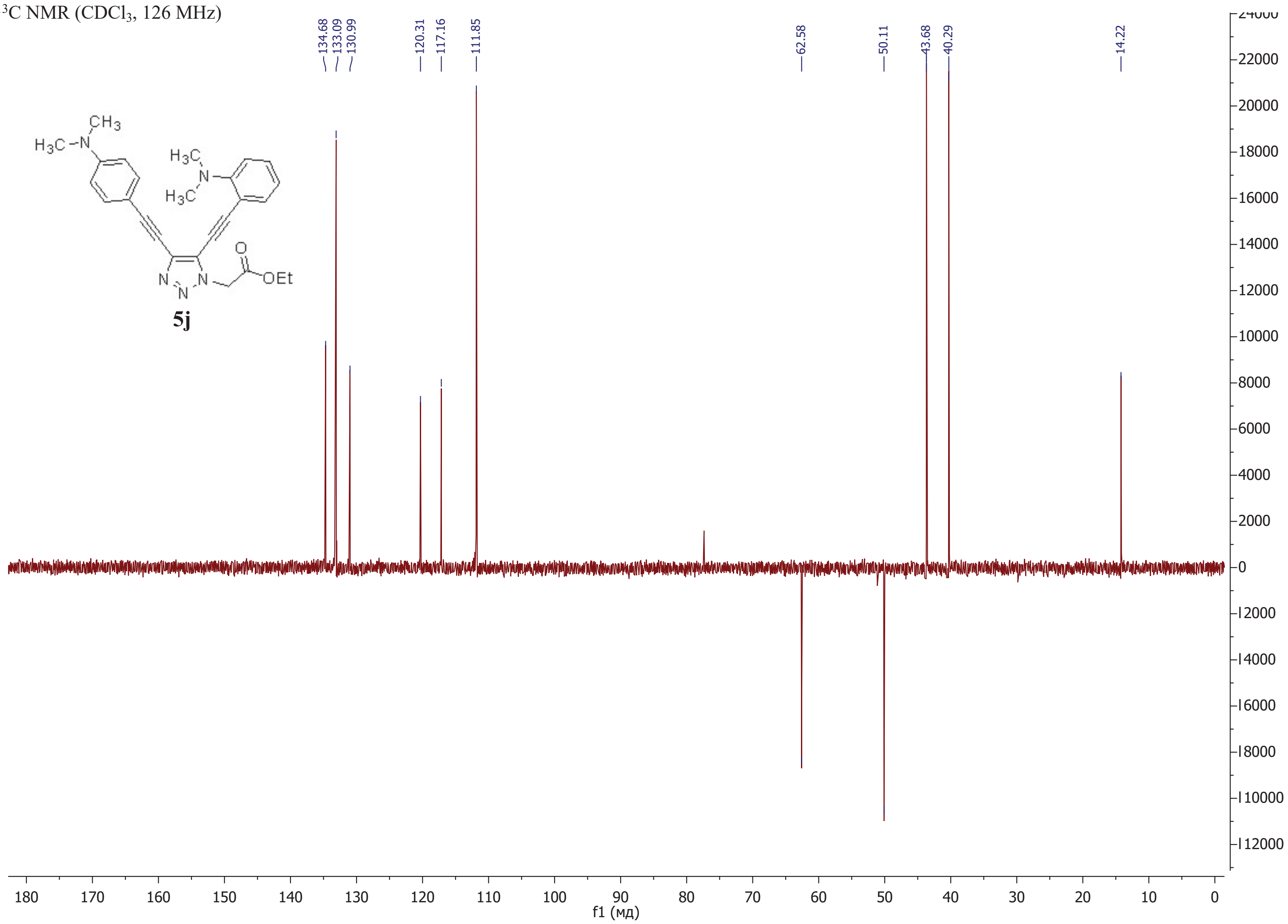

^{1}H NMR ($CDCl_3$, 400 MHz)

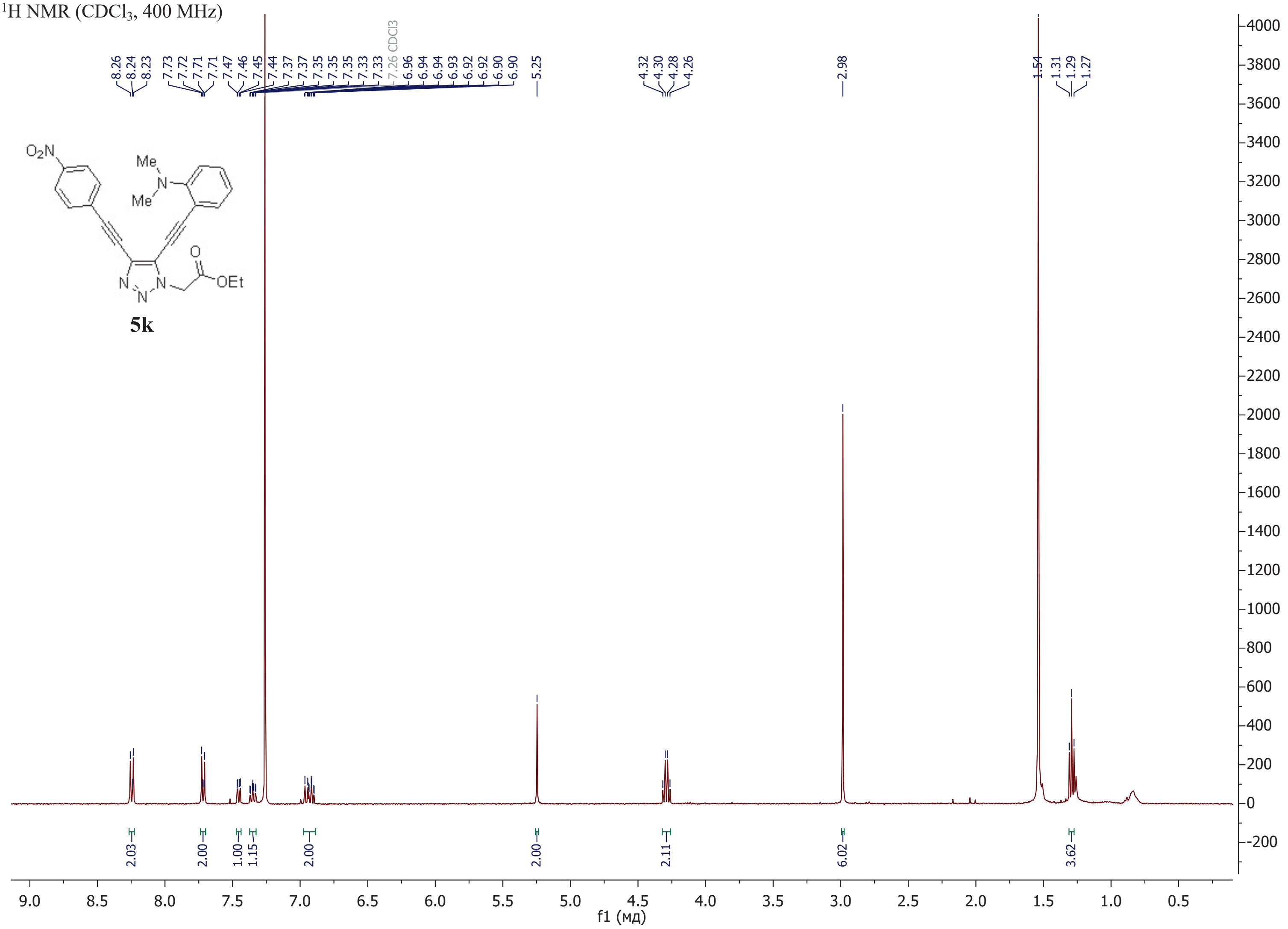

^{13}C NMR ($CDCl_3$, 101 MHz)

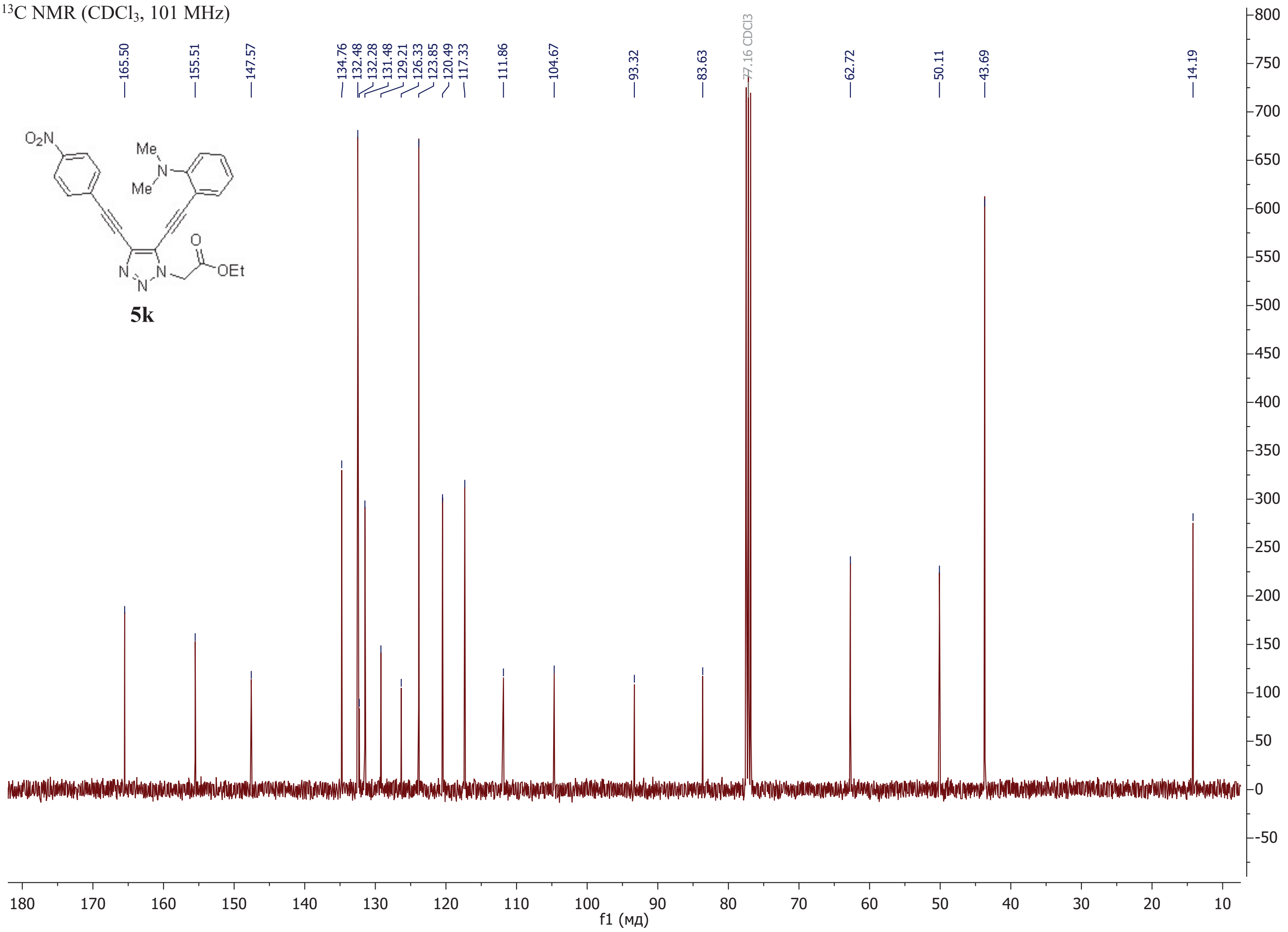

5k

^{13}C NMR ($CDCl_3$, 101 MHz)

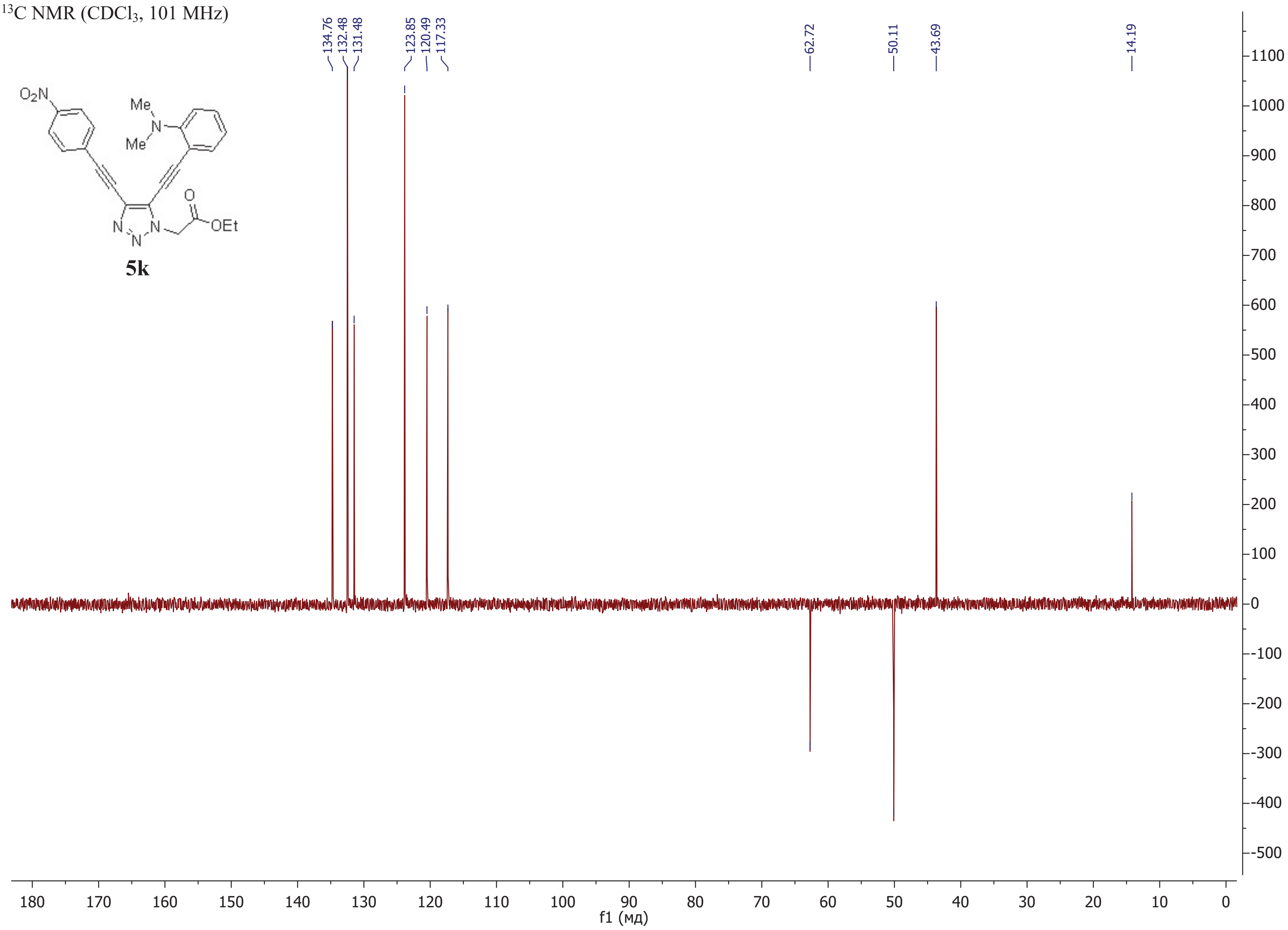

^{1}H NMR ($CDCl_3$, 400 MHz)

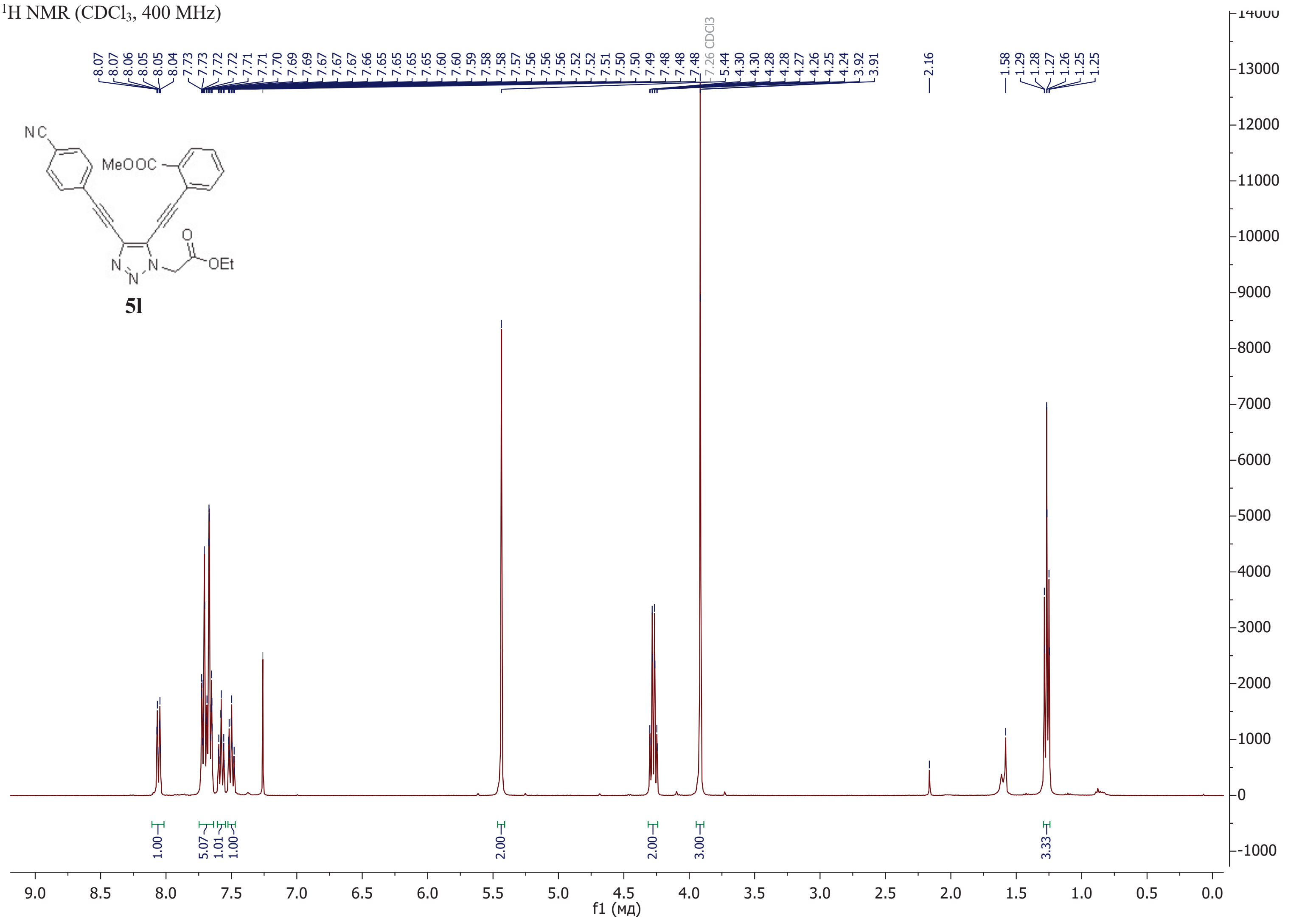

^{13}C NMR ($CDCl_3$, 101 MHz)

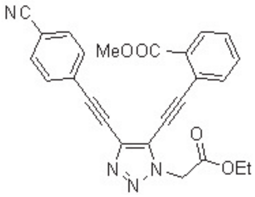

5l

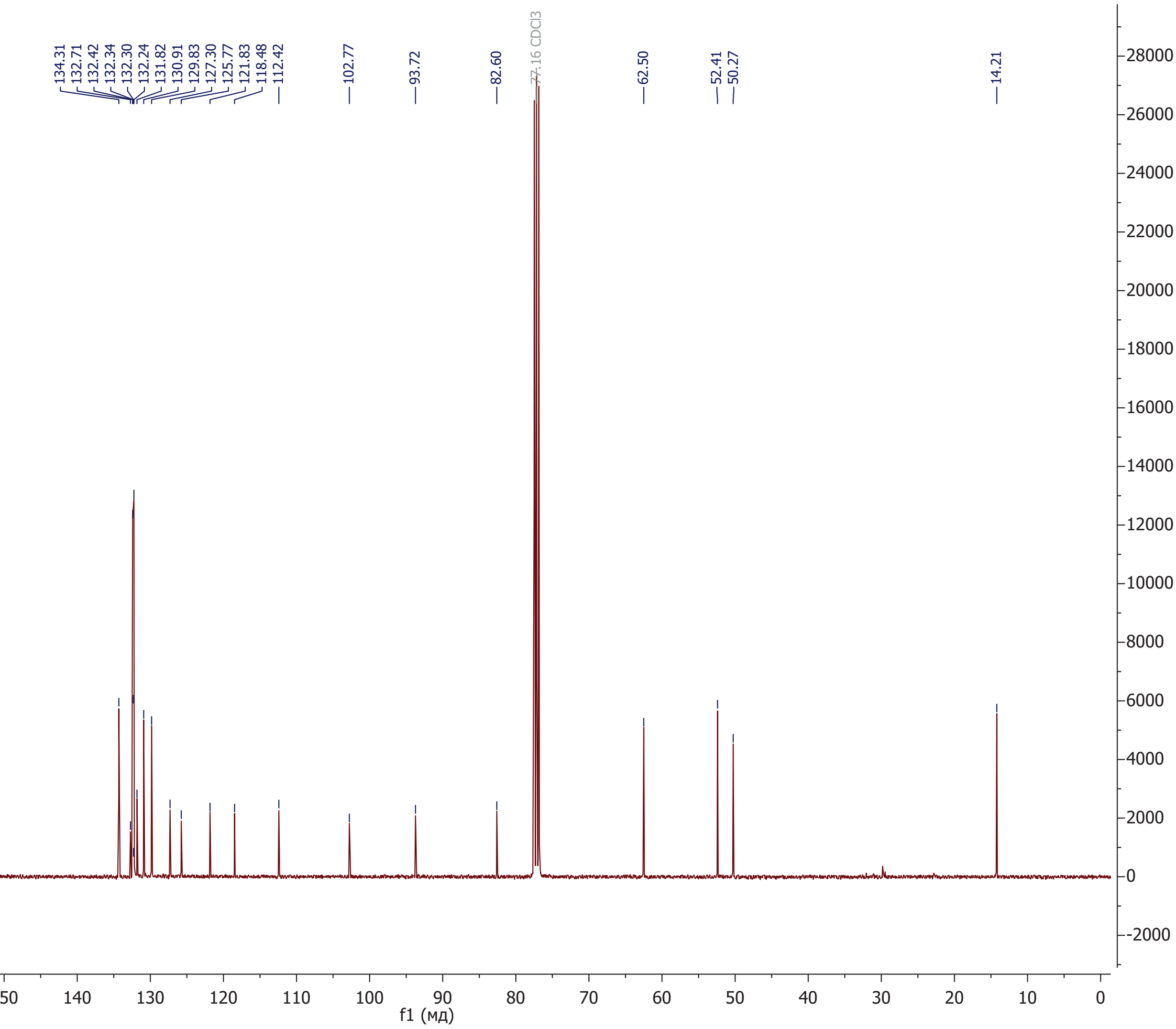

^{13}C NMR ($CDCl_3$, 101 MHz)

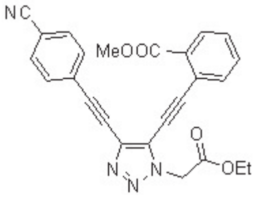

5l

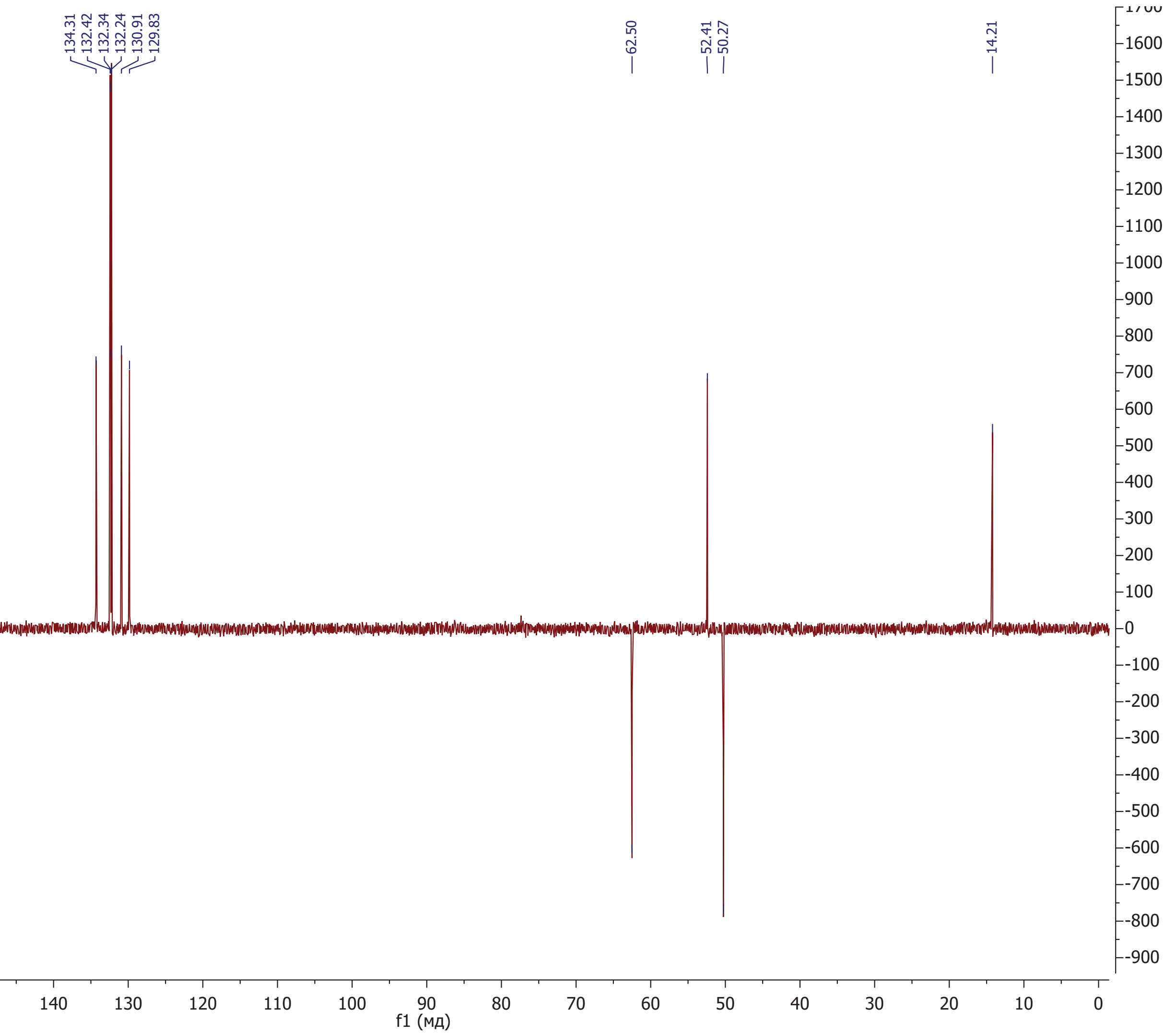

^{1}H NMR ($CDCl_3$, 400 MHz)

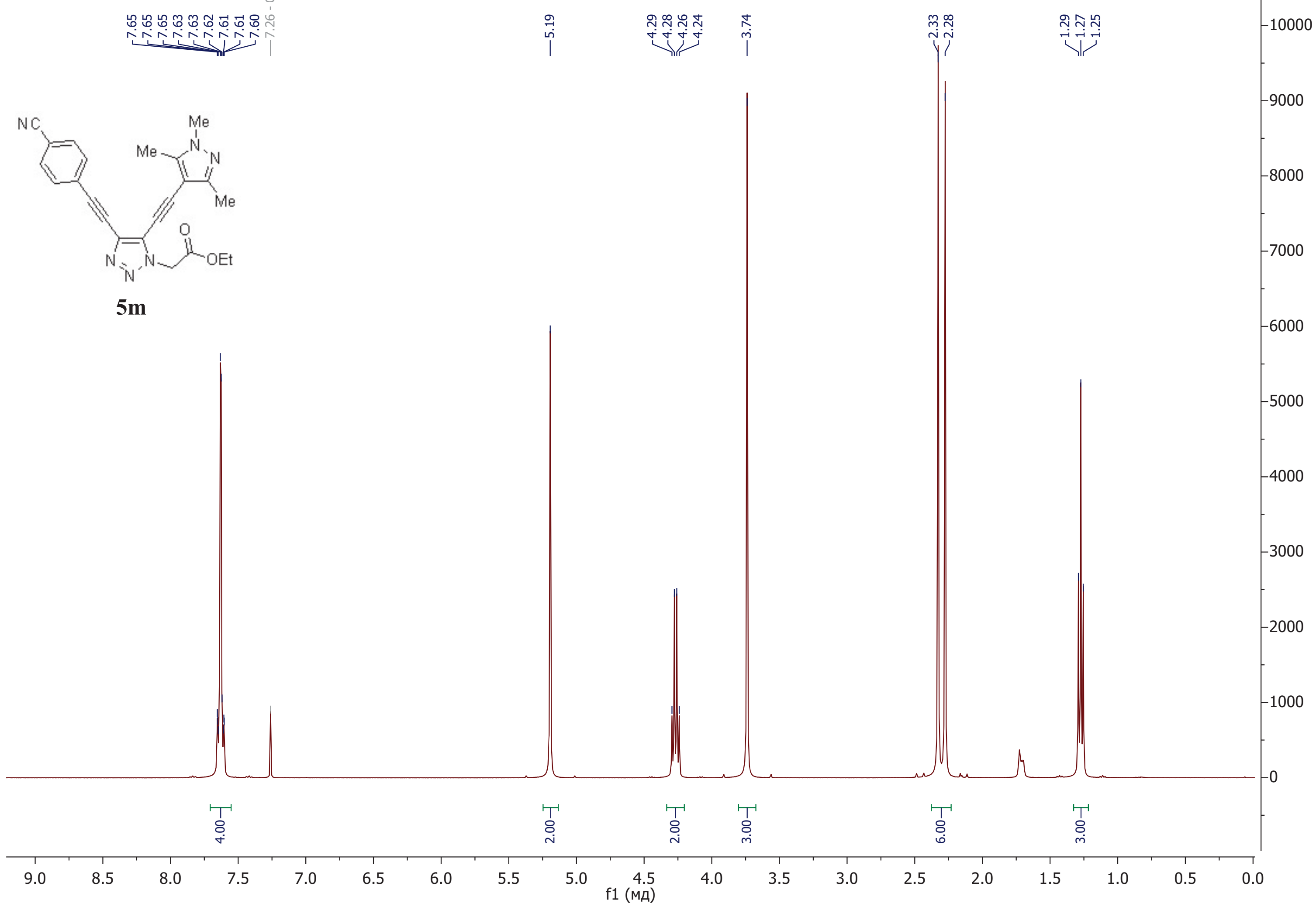

^{13}C NMR ($CDCl_3$, 101 MHz)

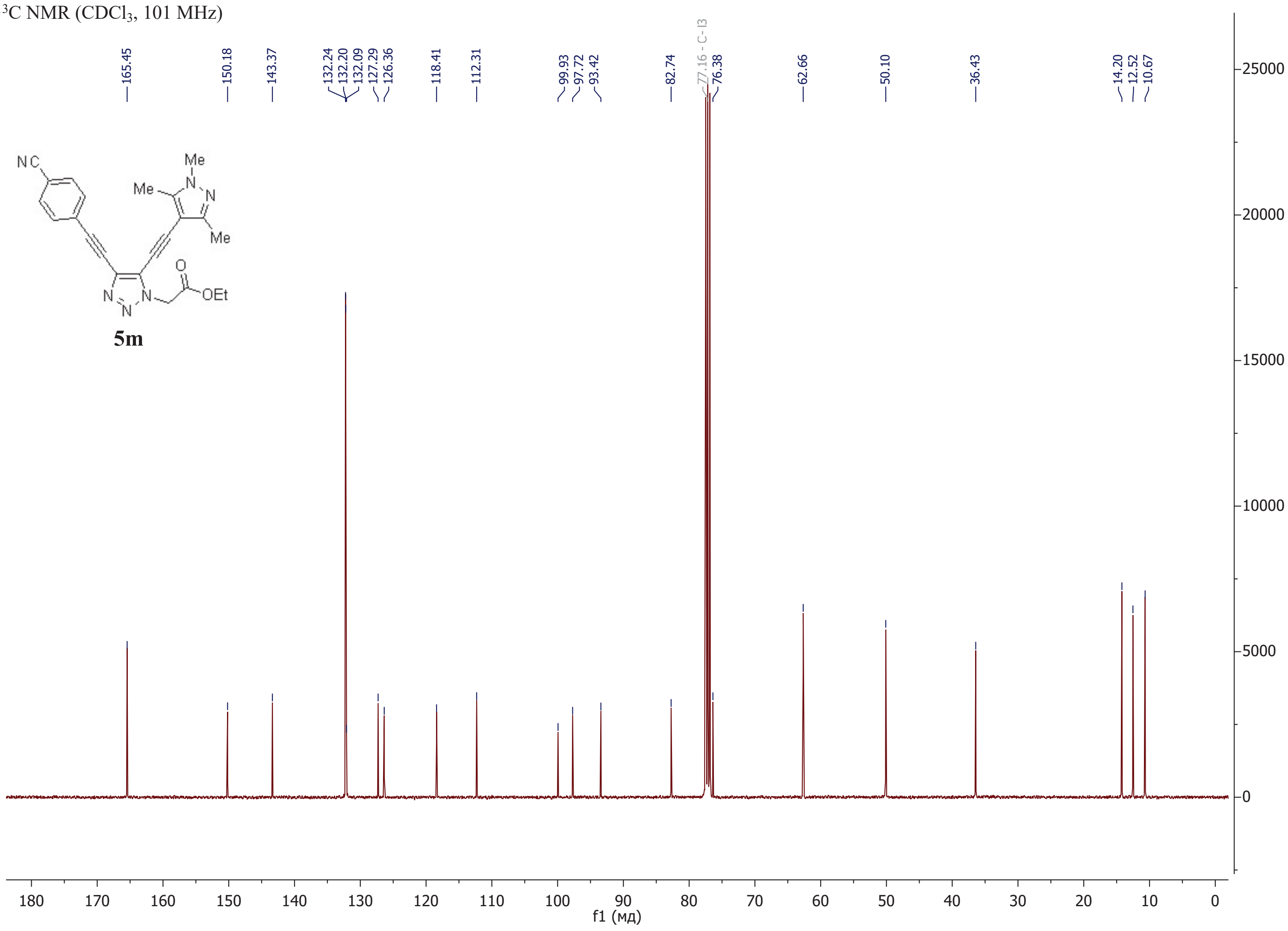

5m

^{13}C NMR ($CDCl_3$, 101 MHz)

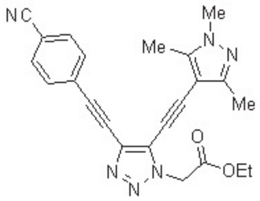

5m

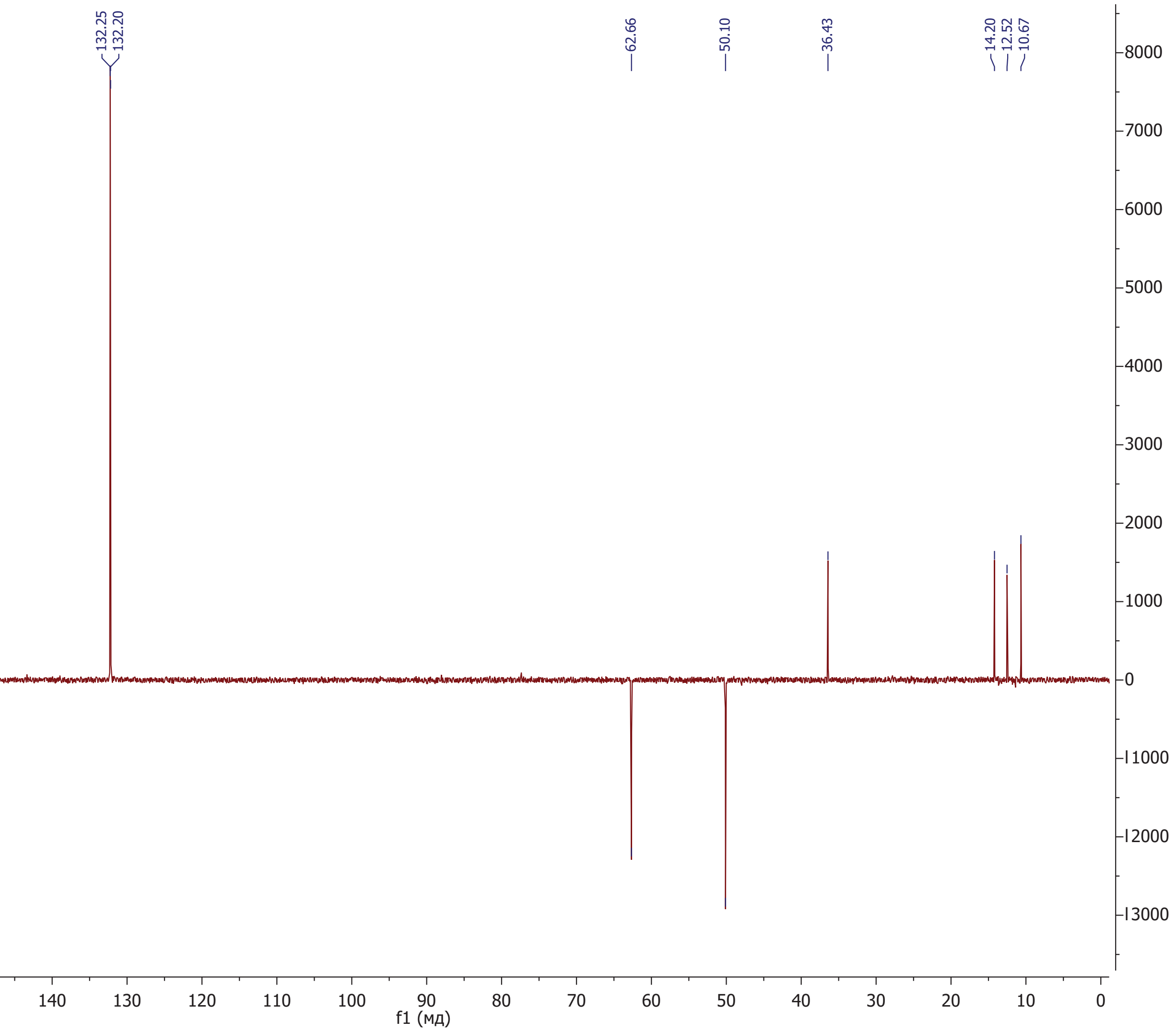

^{1}H NMR ($CDCl_3$, 400 MHz)

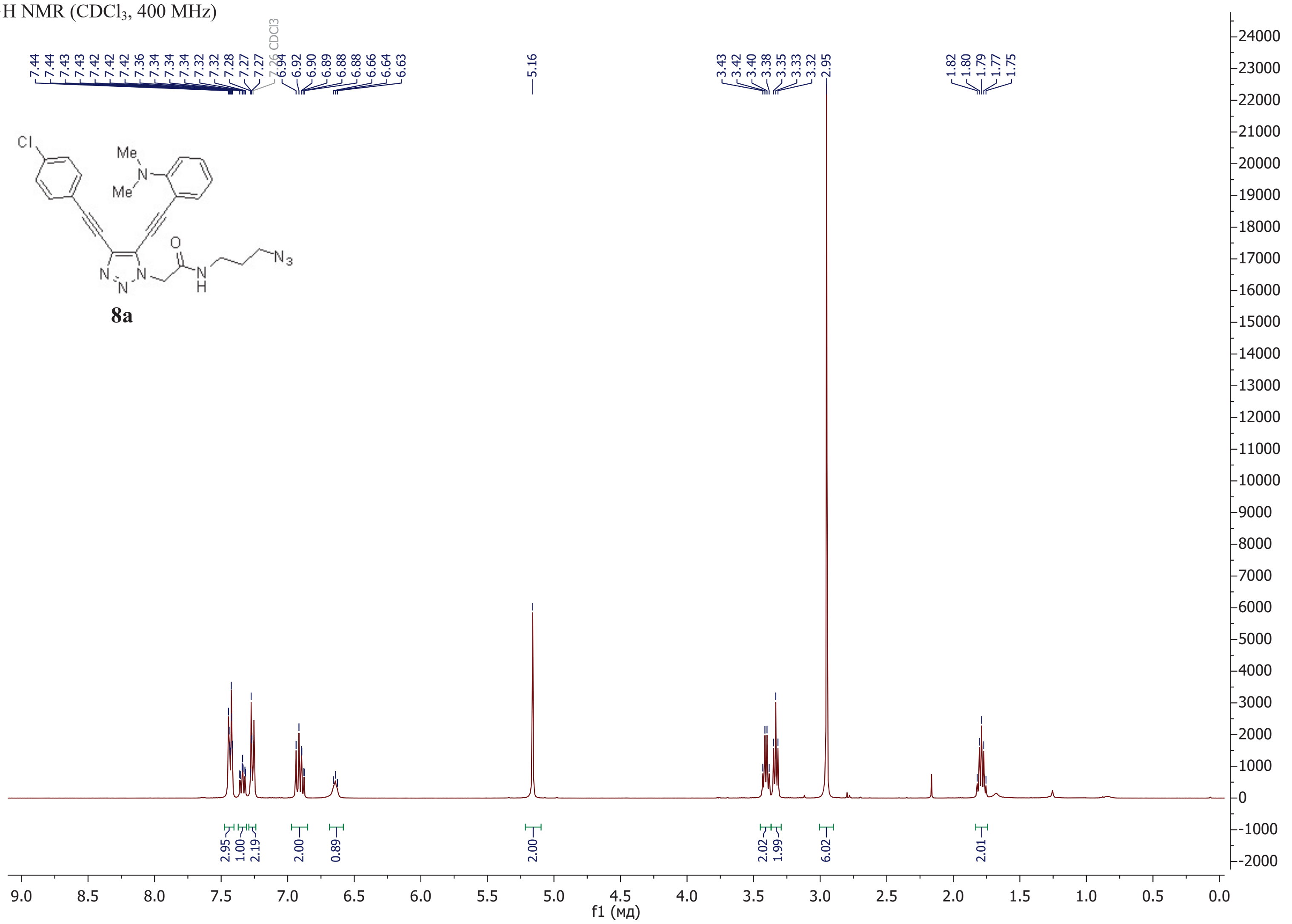

^{13}C NMR ($CDCl_3$, 101 MHz)

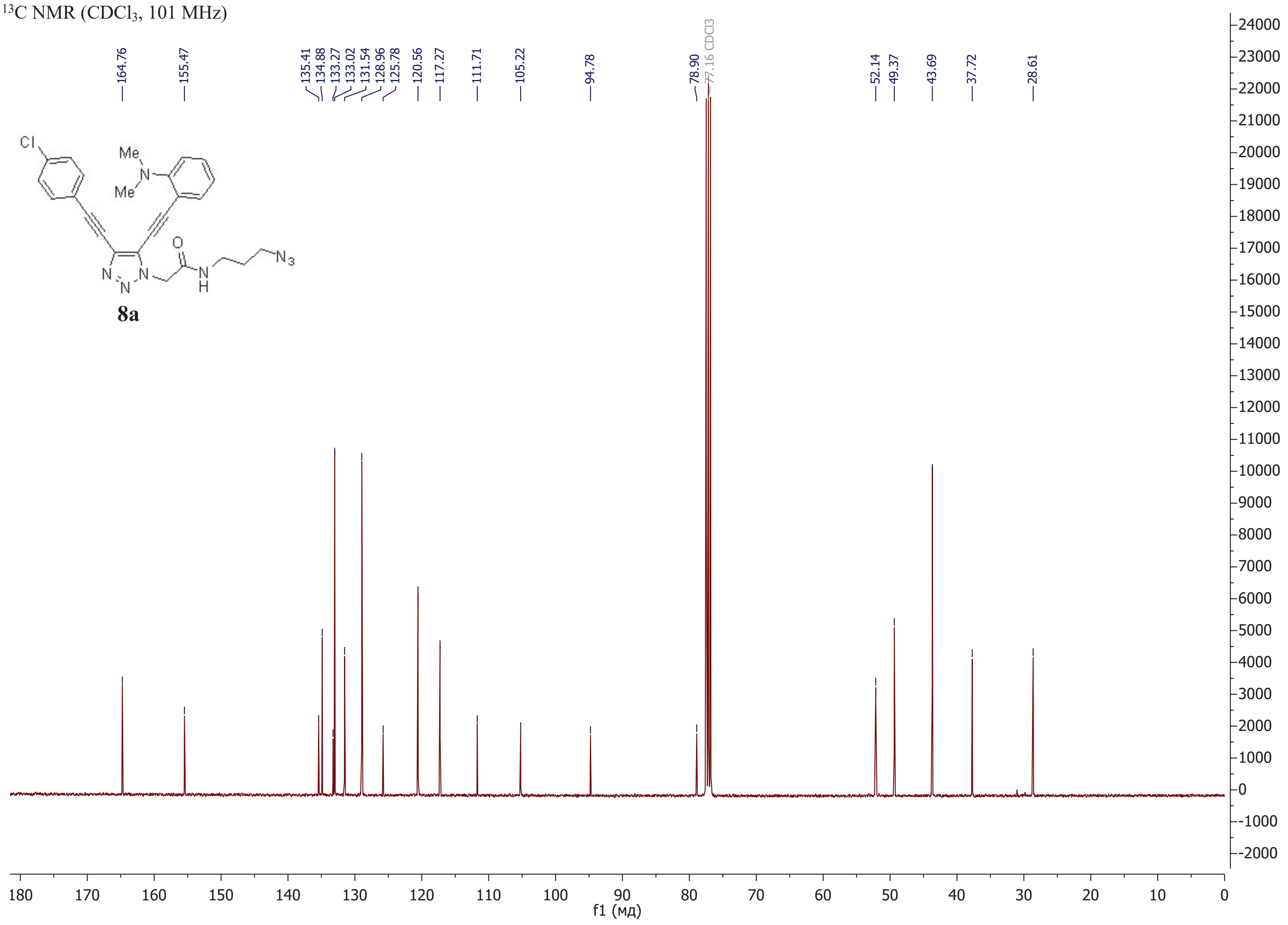

^{13}C NMR ($CDCl_3$, 101 MHz)

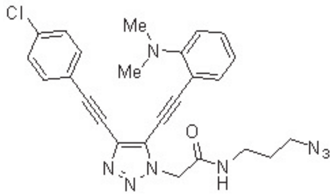

8a

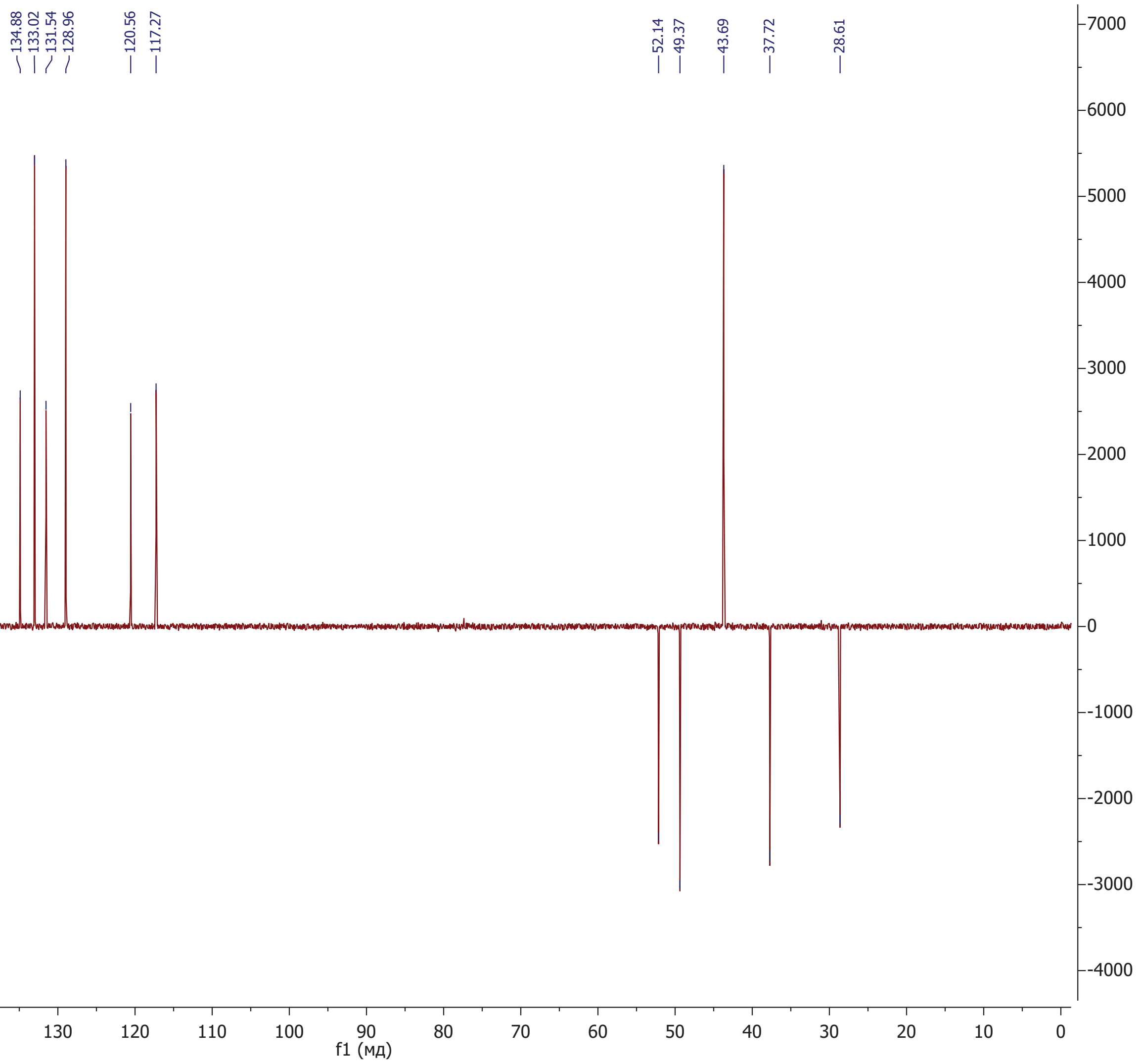

^{1}H NMR ($CDCl_3$, 400 MHz)

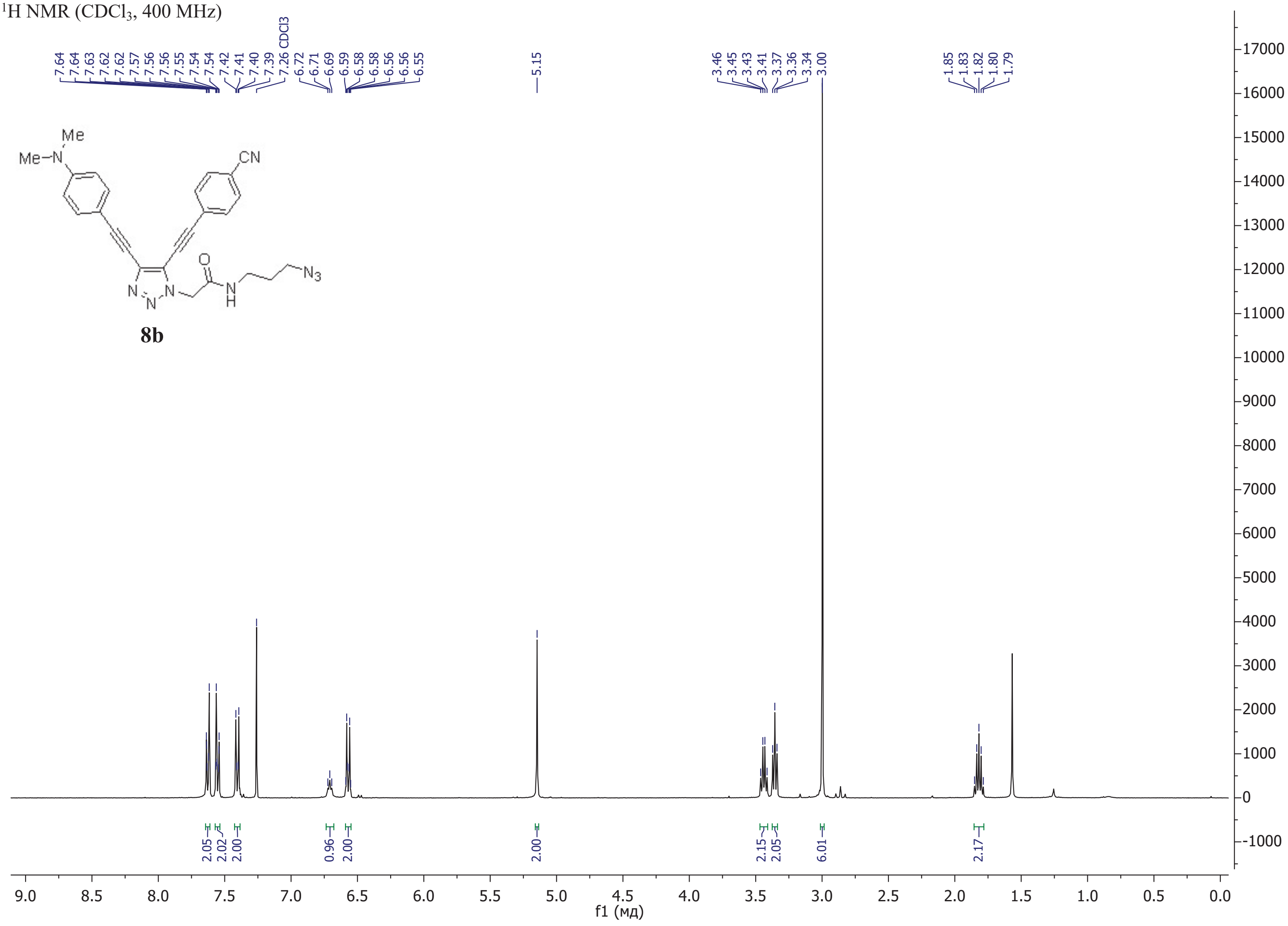

8b

^{13}C NMR ($CDCl_3$, 101 MHz)

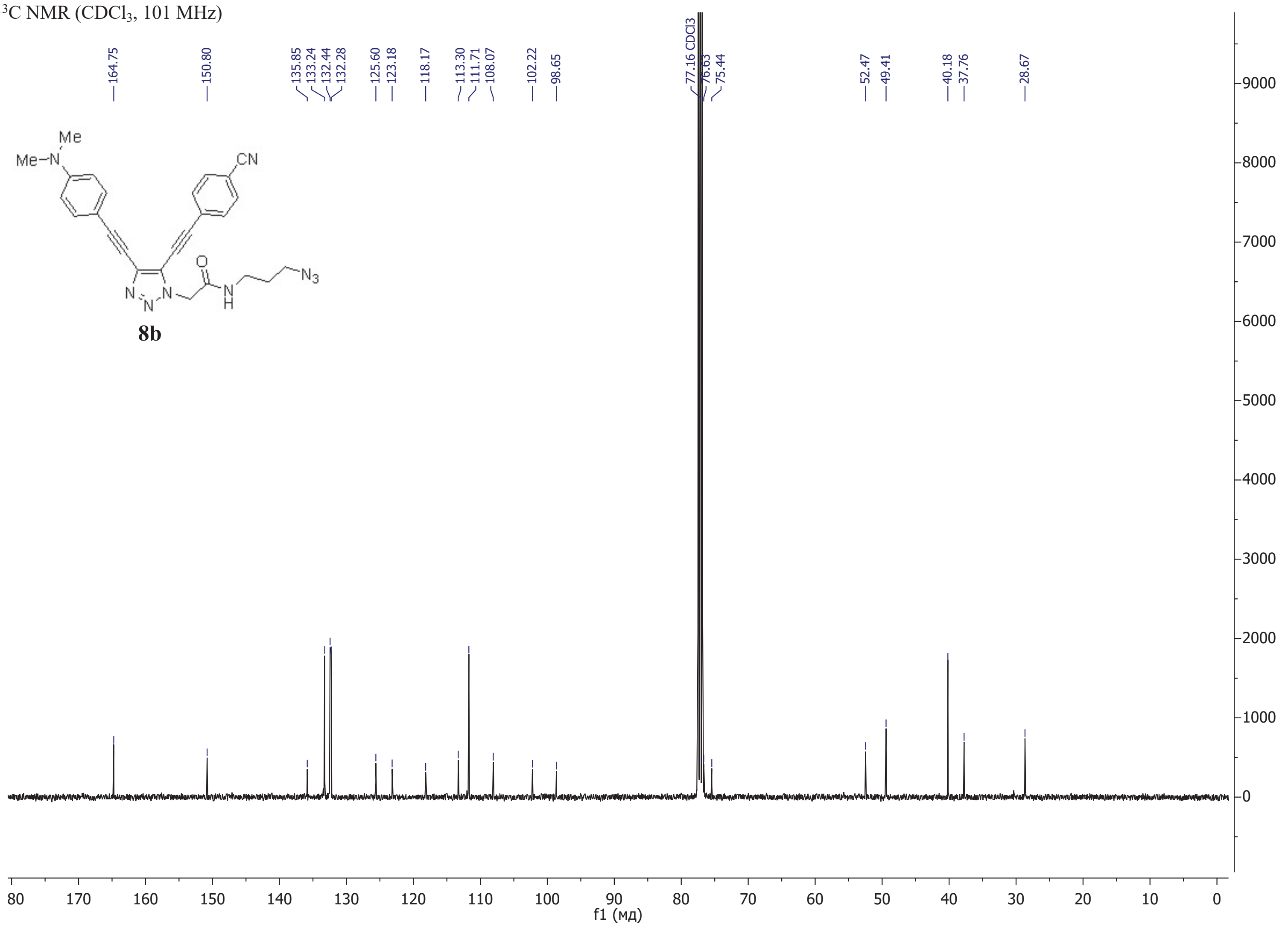

^{13}C NMR ($CDCl_3$, 101 MHz)

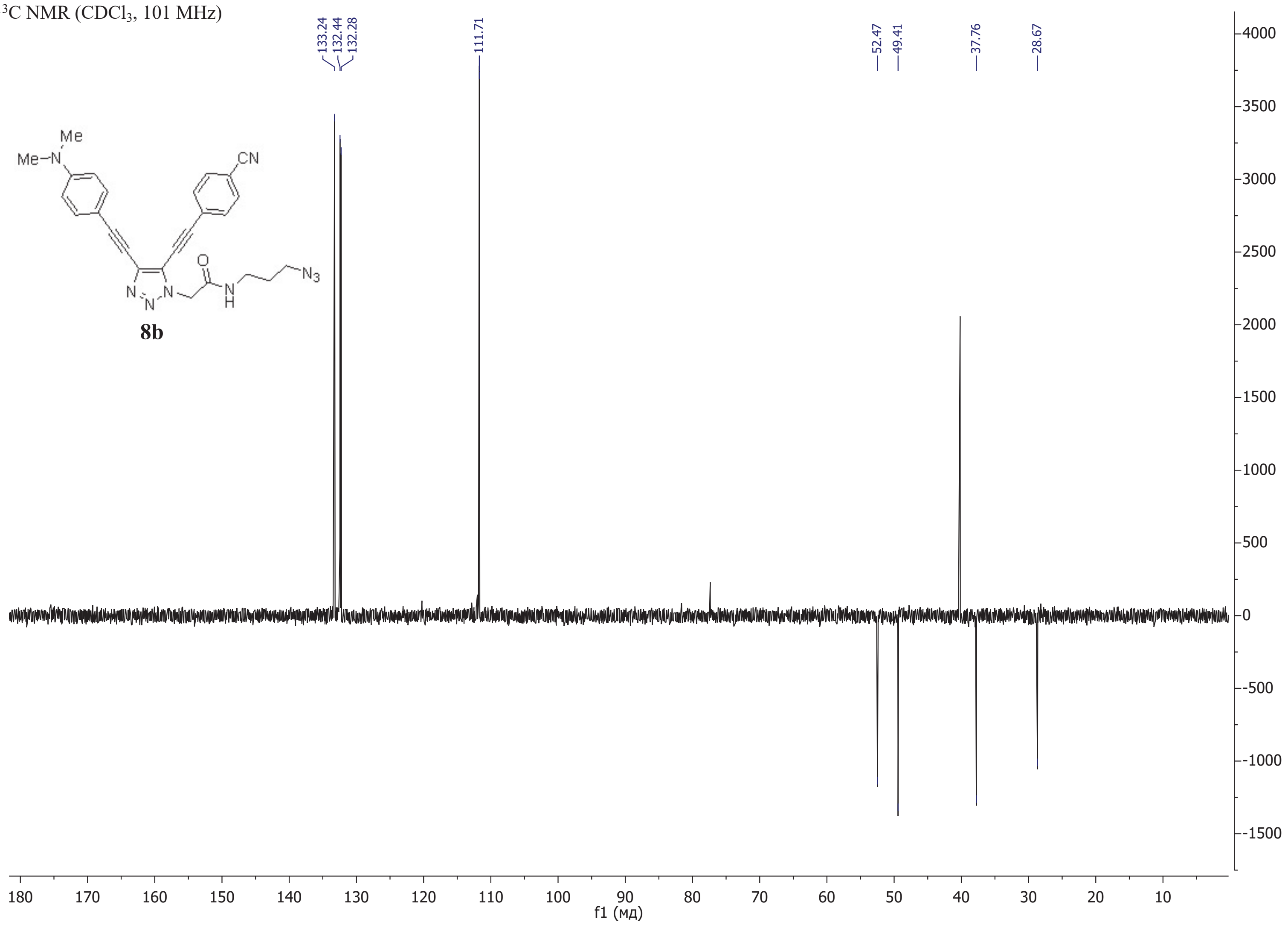

^{1}H NMR ($CDCl_3$, 400 MHz)

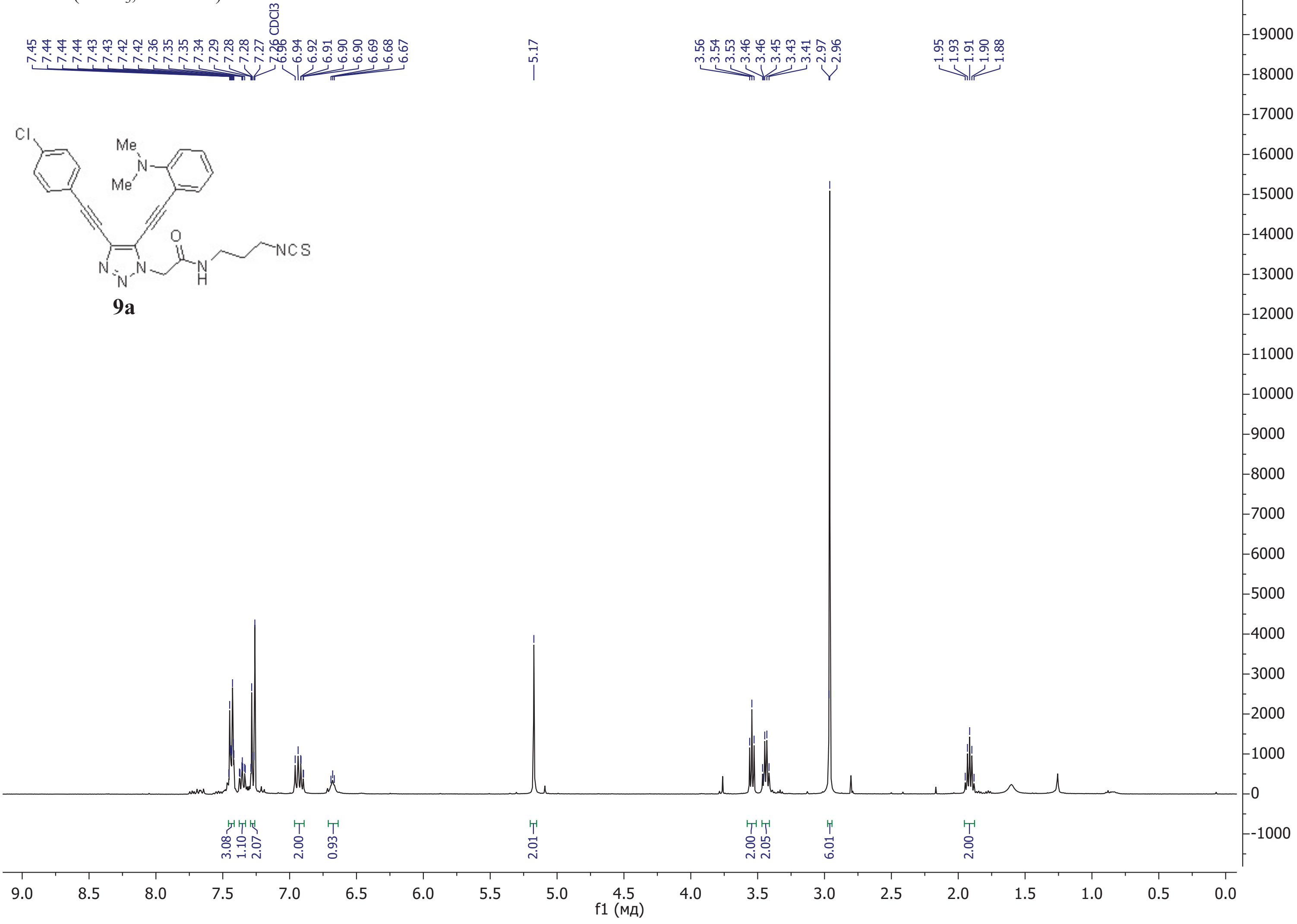

9a

^{13}C NMR ($CDCl_3$, 101 MHz)

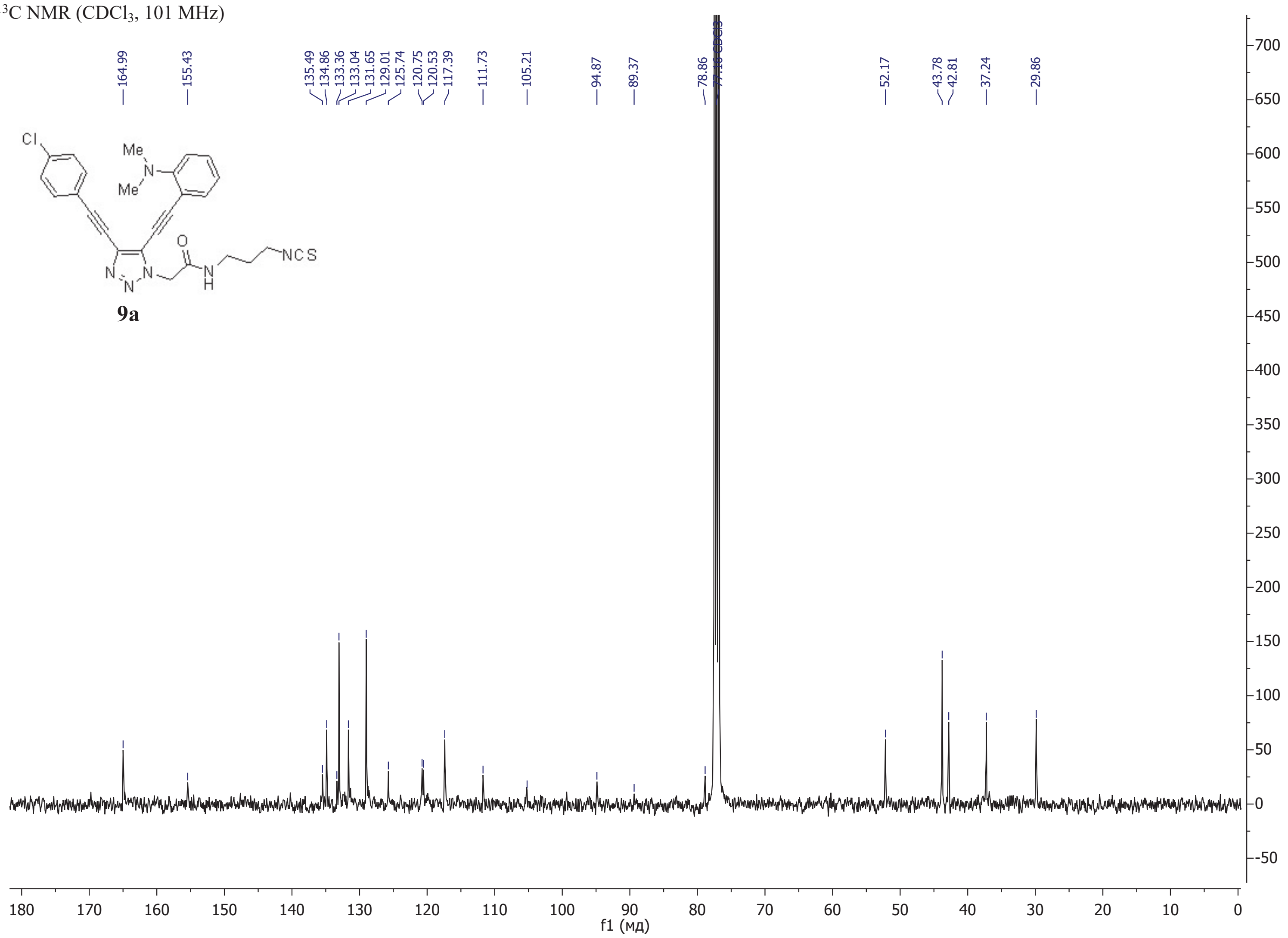

^{13}C NMR ($CDCl_3$, 101 MHz)

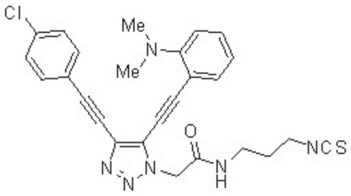

9a

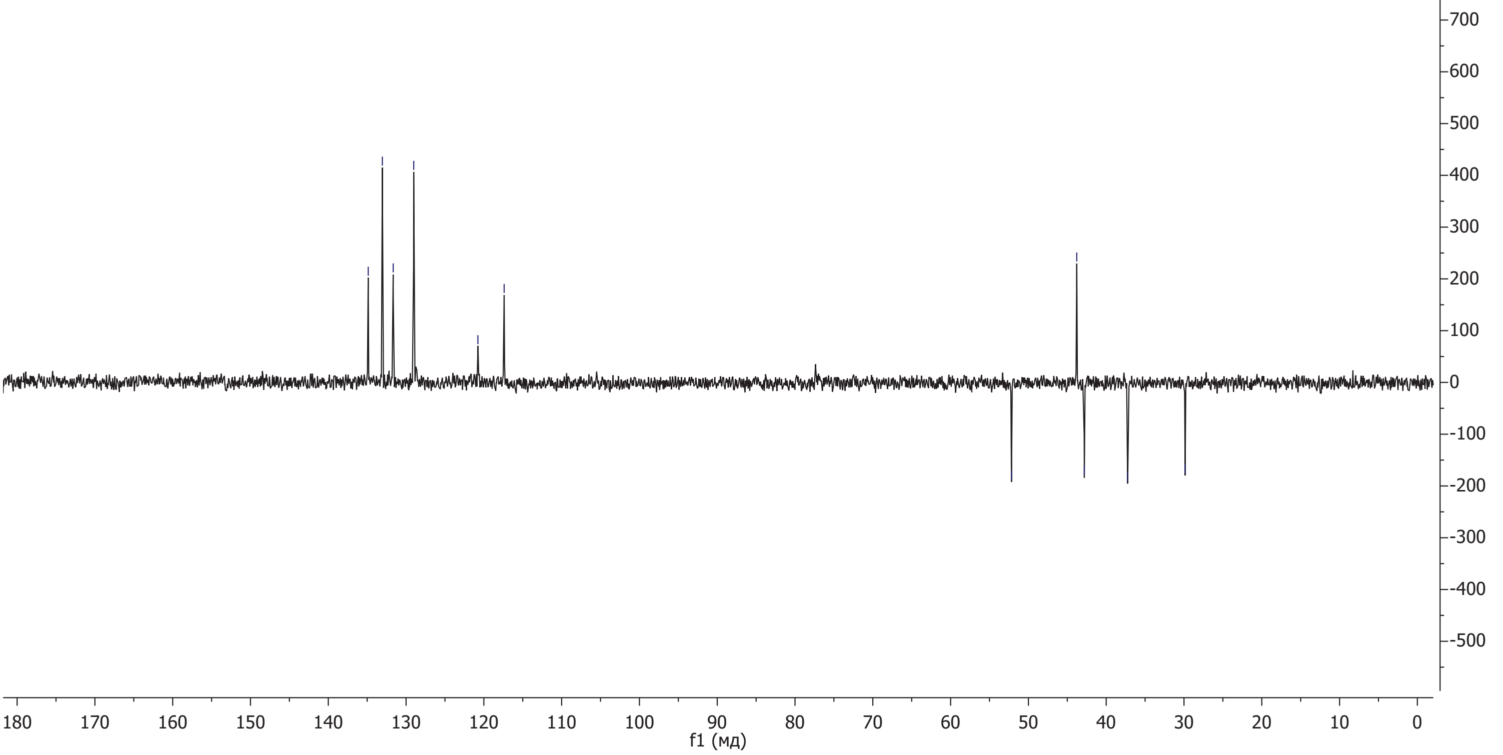

3a

Mass Spectrum Report

Analysis Info

Analysis Name	D:\Data\2018\november\20\TOK62.d	Acquisition Date	20.11.2018 13:13:01
Method	tune_low.m	Operator	Bruker Customer
Sample Name	TOK62	Instrument / Ser#	micrOTOF 10223
Comment	MeOH		

Acquisition Parameter

Source Type	ESI	Ion Polarity	Positive	Set Nebulizer	0.4 Bar
Focus	Not active			Set Dry Heater	180 °C
Scan Begin	50 m/z	Set Capillary	4500 V	Set Dry Gas	4.0 l/min
Scan End	2000 m/z	Set End Plate Offset	-500 V	Set Divert Valve	Source

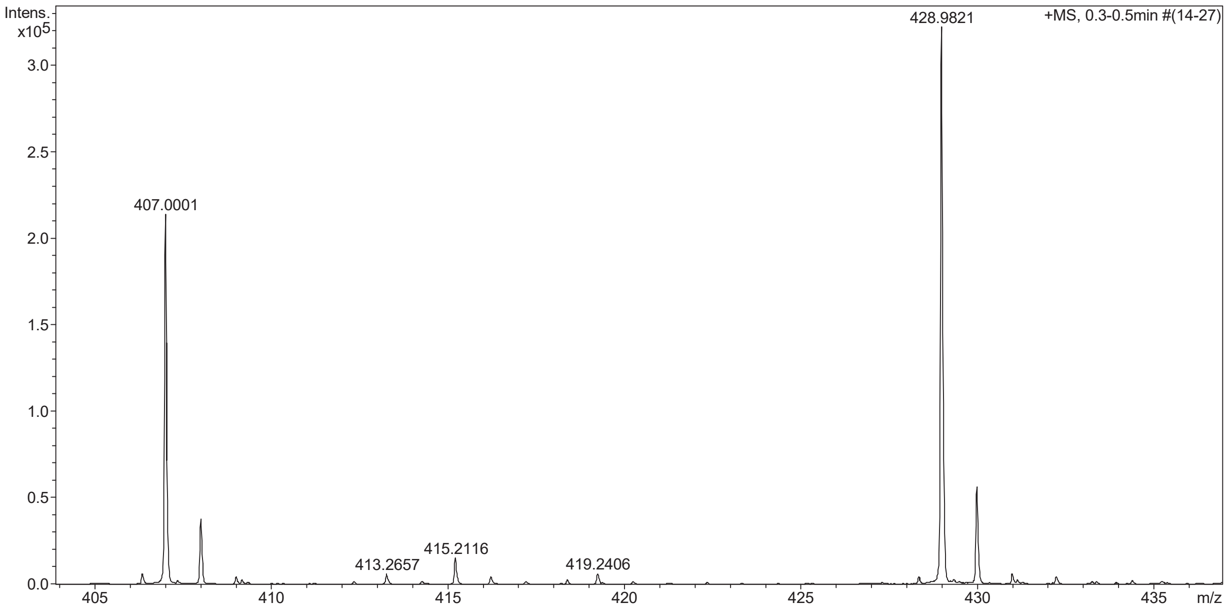

3b

Display Report

Analysis Info

Analysis Name	D:\Data\Work\2018\October\23\TOK_48_000001.d	Acquisition Date	23-Oct-18 14:08:54
Method	tune_wide_pos.m	Operator	BDAL@DE
Sample Name	TOK_48_	Instrument	maXis 62
Comment			

Acquisition Parameter

Source Type	ESI	Ion Polarity	Positive	Set Nebulizer	0.4 Bar
Focus	Active	Set Capillary	4500 V	Set Dry Heater	180 °C
Scan Begin	300 m/z	Set End Plate Offset	-500 V	Set Dry Gas	4.0 l/min
Scan End	3000 m/z	Set Collision Cell RF	1000.0 Vpp	Set Divert Valve	Source

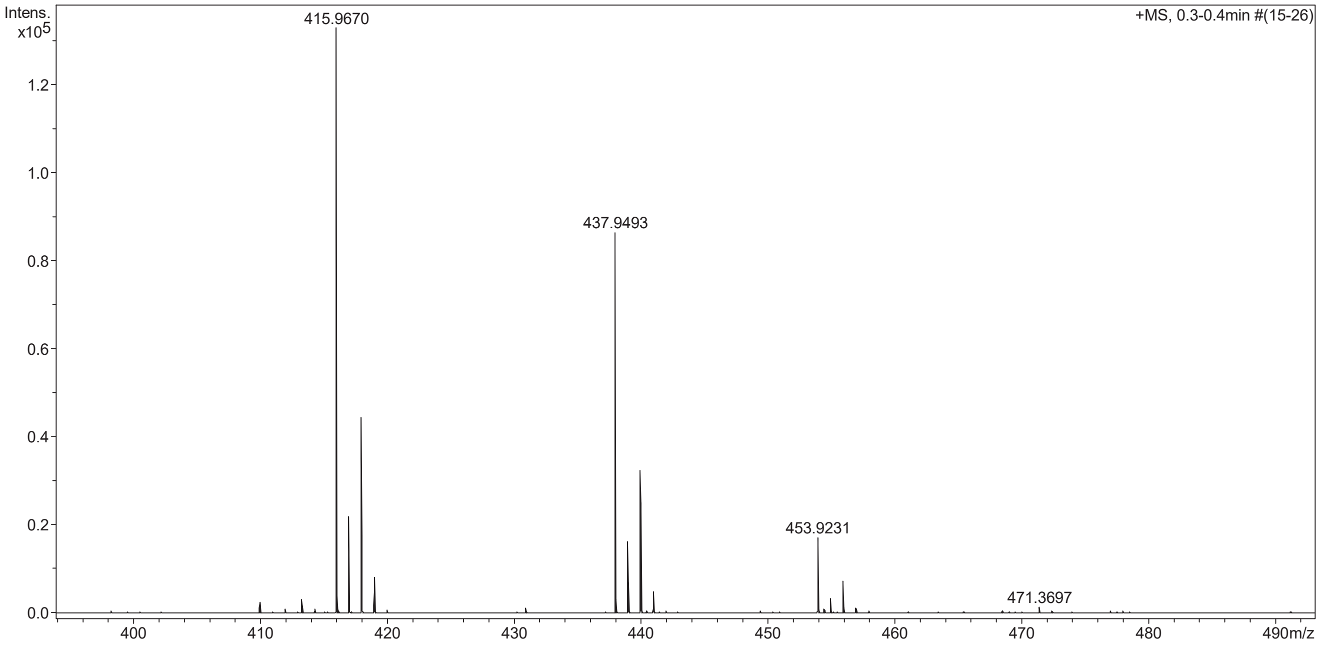

Create PDF files without this message by purchasing novaPDF printer (http://www.novapdf.com)

3c

Mass Spectrum Report

Analysis Info

Analysis Name	D:\Data\2018\november\13\TOK58.d	Acquisition Date	13.11.2018 10:18:33
Method	tune_low.m	Operator	Bruker Customer
Sample Name	TOK58	Instrument / Ser#	micrOTOF 10223
Comment	MeOH		

Acquisition Parameter

Source Type	ESI	Ion Polarity	Positive	Set Nebulizer	0.4 Bar
Focus	Not active			Set Dry Heater	180 °C
Scan Begin	50 m/z	Set Capillary	4500 V	Set Dry Gas	4.0 l/min
Scan End	3000 m/z	Set End Plate Offset	-500 V	Set Divert Valve	Source

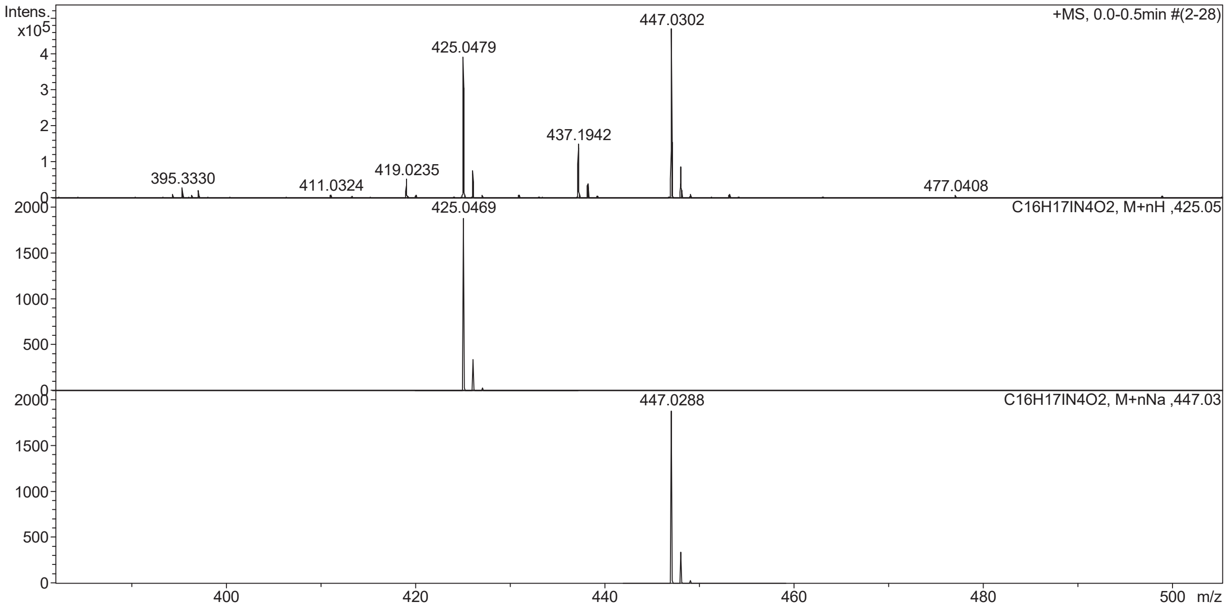

3d

Mass Spectrum Report

Analysis Info

Analysis Name	D:\Data\2018\october\05\TOK38.d	Acquisition Date	05.10.2018 9:49:27
Method	tune_low.m	Operator	Bruker Customer
Sample Name	TOK38	Instrument / Ser#	micrOTOF 10223
Comment	MeOH		

Acquisition Parameter

Source Type	ESI	Ion Polarity	Positive	Set Nebulizer	0.4 Bar
Focus	Not active			Set Dry Heater	180 °C
Scan Begin	50 m/z	Set Capillary	4500 V	Set Dry Gas	4.0 l/min
Scan End	3000 m/z	Set End Plate Offset	-500 V	Set Divert Valve	Source

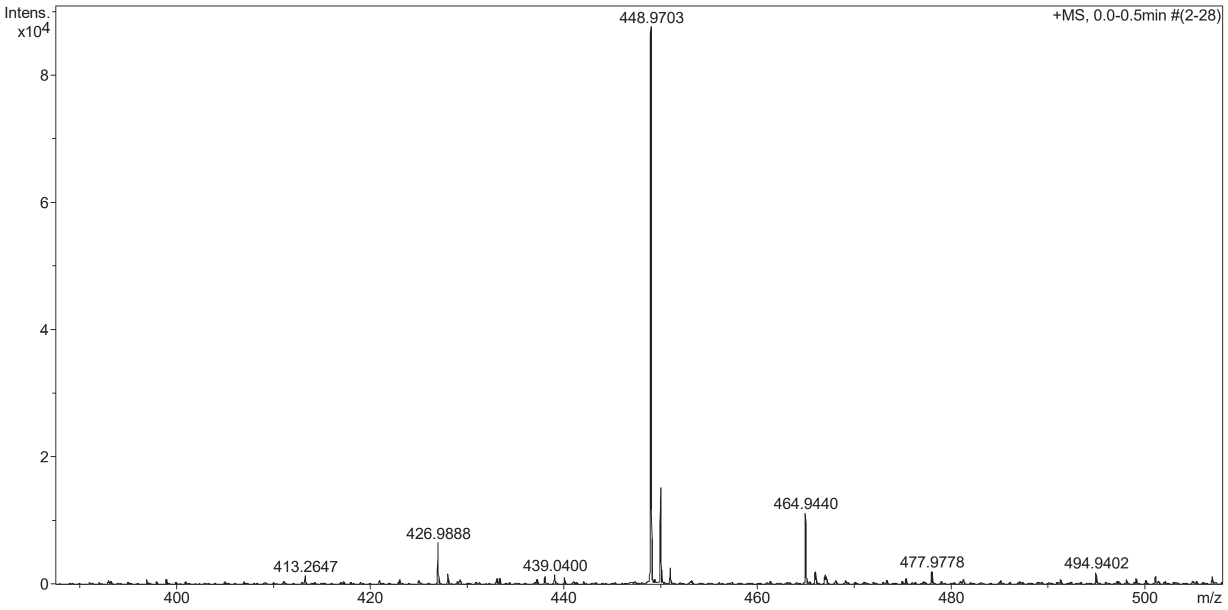

5a

Mass Spectrum Report

Analysis Info

Analysis Name	D:\Data\2019\april\09\TOK128.d	Acquisition Date	09.04.2019 17:30:18
Method	tune_low.m	Operator	Bruker Customer
Sample Name	TOK128	Instrument / Ser#	micrOTOF 10223
Comment	MeOH		

Acquisition Parameter

Source Type	ESI	Ion Polarity	Positive	Set Nebulizer	0.4 Bar
Focus	Not active			Set Dry Heater	180 °C
Scan Begin	50 m/z	Set Capillary	4500 V	Set Dry Gas	4.0 l/min
Scan End	3000 m/z	Set End Plate Offset	-500 V	Set Divert Valve	Source

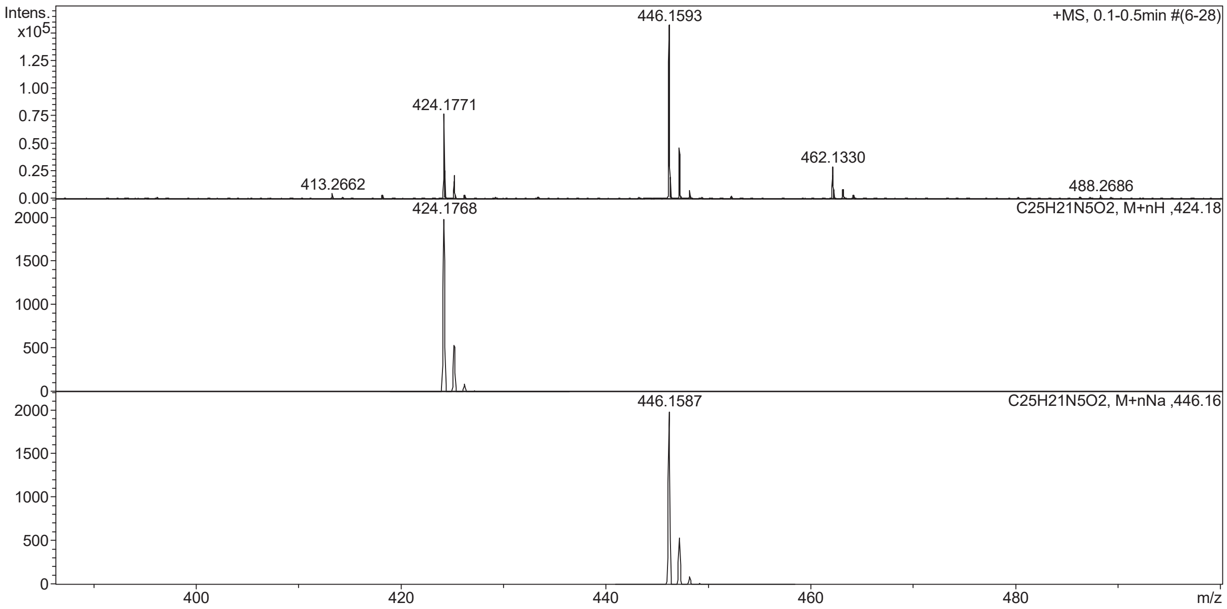

5b

Mass Spectrum Report

Analysis Info

Analysis Name	D:\Data\2019\february\08\TOK102.d	Acquisition Date	08.02.2019 14:19:51
Method	tune_low.m	Operator	Bruker Customer
Sample Name	TOK102	Instrument / Ser#	micrOTOF 10223
Comment	MeOH		

Acquisition Parameter

Source Type	ESI	Ion Polarity	Positive	Set Nebulizer	0.4 Bar
Focus	Not active			Set Dry Heater	180 °C
Scan Begin	50 m/z	Set Capillary	4500 V	Set Dry Gas	4.0 l/min
Scan End	3000 m/z	Set End Plate Offset	-500 V	Set Divert Valve	Source

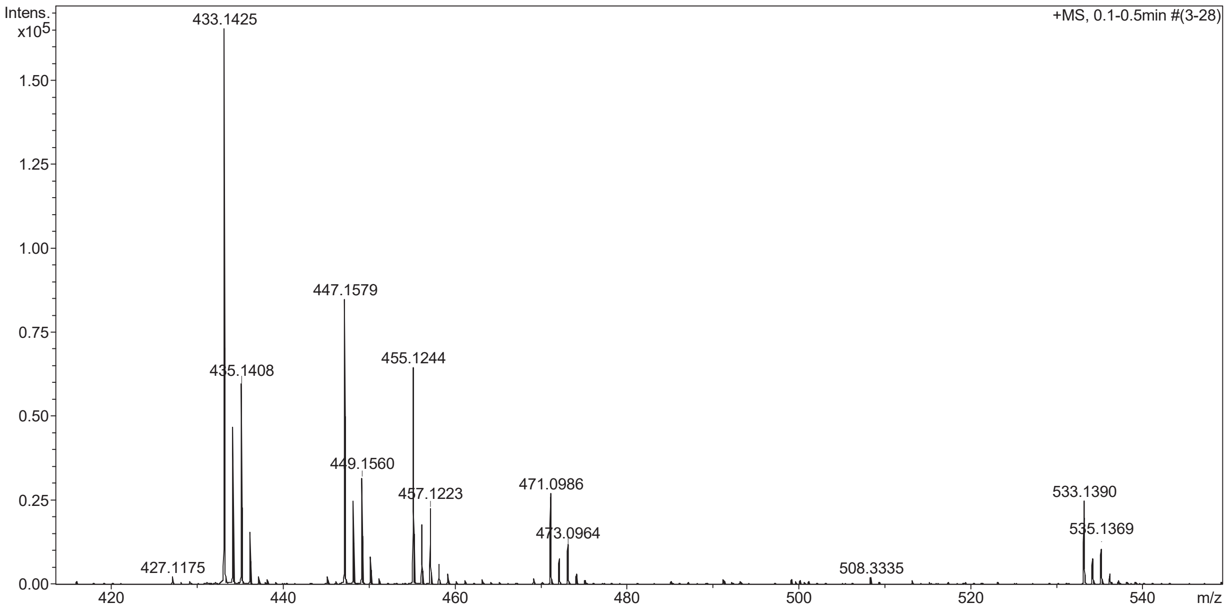

5c

Mass Spectrum Report

**Analysis Info**		Acquisition Date	03.12.2018 13:48:26
Analysis Name	D:\Data\2018\december\3\TOK70.d		
Method	tune_low.m	Operator	Bruker Customer
Sample Name	TOK70	Instrument / Ser#	micrOTOF 10223
Comment	MeOH 50v		

Acquisition Parameter

Source Type	ESI	Ion Polarity	Positive	Set Nebulizer	0.4 Bar
Focus	Not active			Set Dry Heater	180 °C
Scan Begin	50 m/z	Set Capillary	4500 V	Set Dry Gas	4.0 l/min
Scan End	3000 m/z	Set End Plate Offset	-500 V	Set Divert Valve	Source

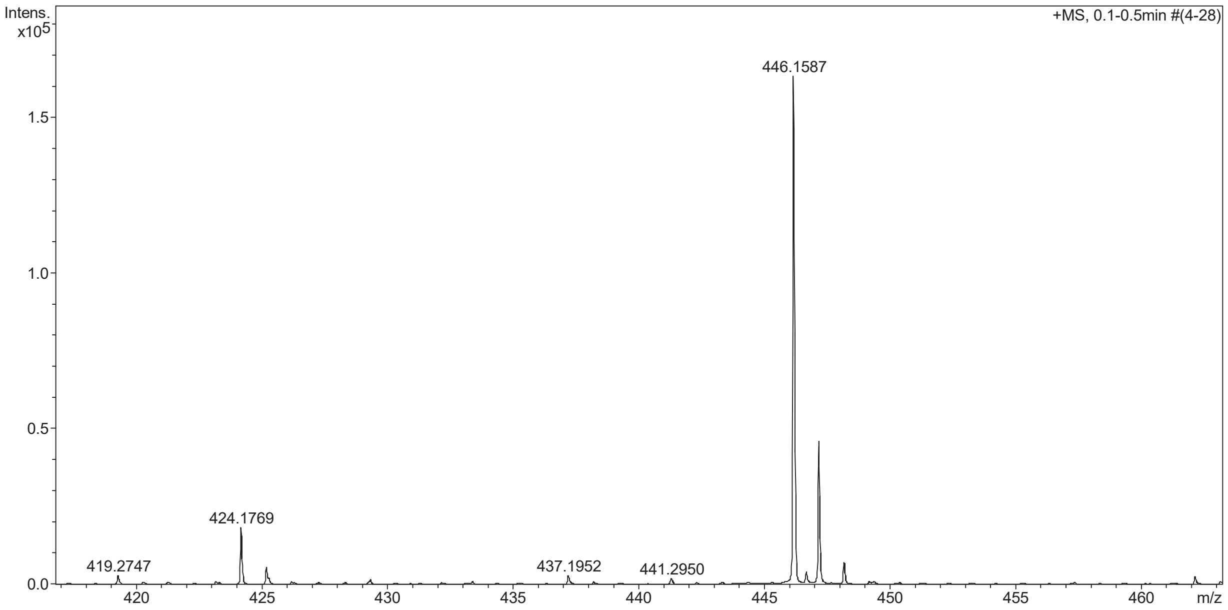

5d

Mass Spectrum Report

Analysis Info

Analysis Name	D:\Data\2019\february\08\TOK101.d	Acquisition Date	08.02.2019 14:05:29
Method	tune_low.m	Operator	Bruker Customer
Sample Name	TOK101	Instrument / Ser#	micrOTOF 10223
Comment	MeOH		

Acquisition Parameter

Source Type	ESI	Ion Polarity	Positive	Set Nebulizer	0.4 Bar
Focus	Not active			Set Dry Heater	180 °C
Scan Begin	50 m/z	Set Capillary	4500 V	Set Dry Gas	4.0 l/min
Scan End	3000 m/z	Set End Plate Offset	-500 V	Set Divert Valve	Source

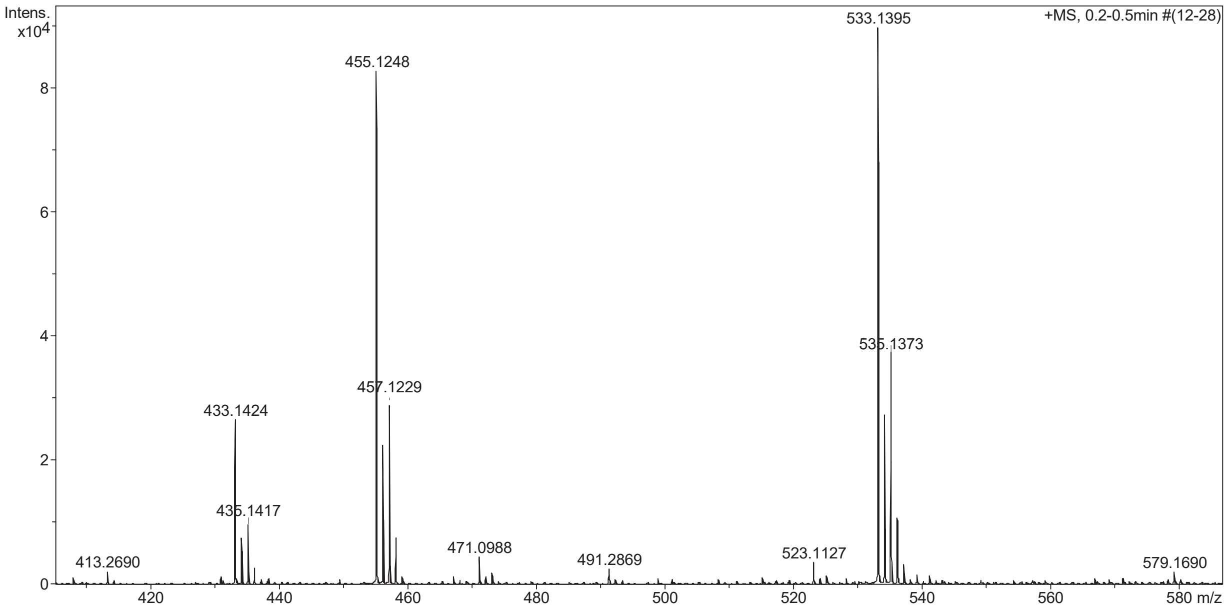

5e

Mass Spectrum Report

Analysis Info

Analysis Name	D:\Data\2018\december\3\TOK71.d	Acquisition Date	03.12.2018 13:57:39
Method	tune_low.m	Operator	Bruker Customer
Sample Name	TOK71	Instrument / Ser#	micrOTOF 10223
Comment	MeOH 50v		

Acquisition Parameter

Source Type	ESI	Ion Polarity	Positive	Set Nebulizer	0.4 Bar
Focus	Not active			Set Dry Heater	180 °C
Scan Begin	50 m/z	Set Capillary	4500 V	Set Dry Gas	4.0 l/min
Scan End	3000 m/z	Set End Plate Offset	-500 V	Set Divert Valve	Source

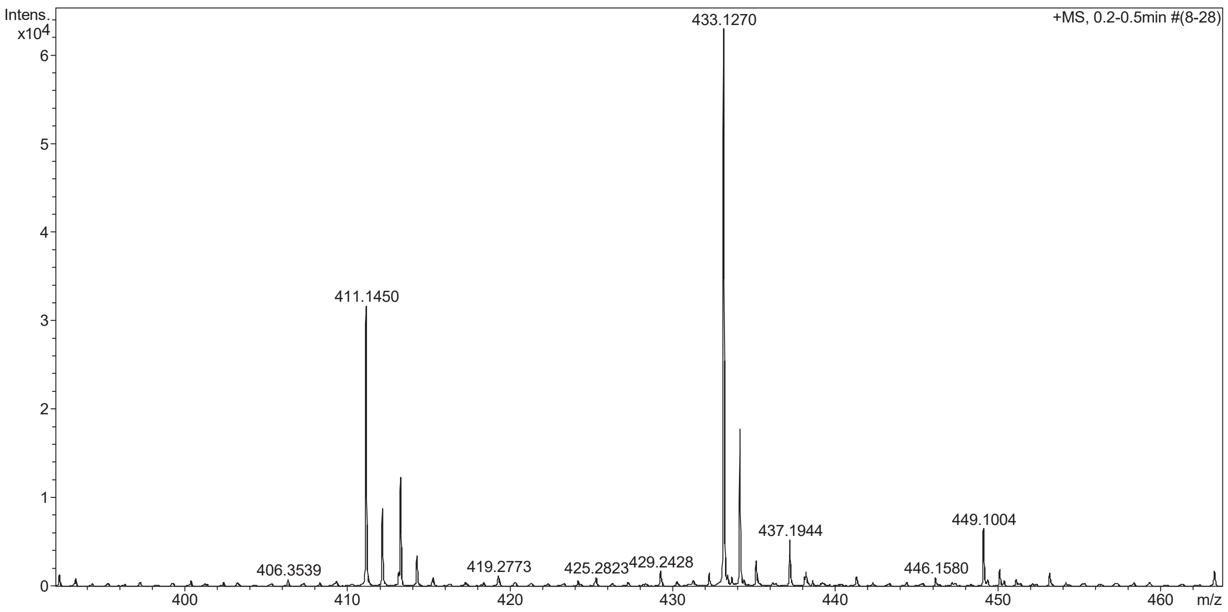

5f

Mass Spectrum Report

Analysis Info

Analysis Name	D:\Data\2019\january\21\TOK64.d	Acquisition Date	21.01.2019 14:21:16
Method	tune_low.m	Operator	Bruker Customer
Sample Name	TOK64	Instrument / Ser#	micrOTOF 10223
Comment	MeOH		

Acquisition Parameter

Source Type	ESI	Ion Polarity	Positive	Set Nebulizer	0.4 Bar
Focus	Not active			Set Dry Heater	180 °C
Scan Begin	50 m/z	Set Capillary	4500 V	Set Dry Gas	4.0 l/min
Scan End	3000 m/z	Set End Plate Offset	-500 V	Set Divert Valve	Source

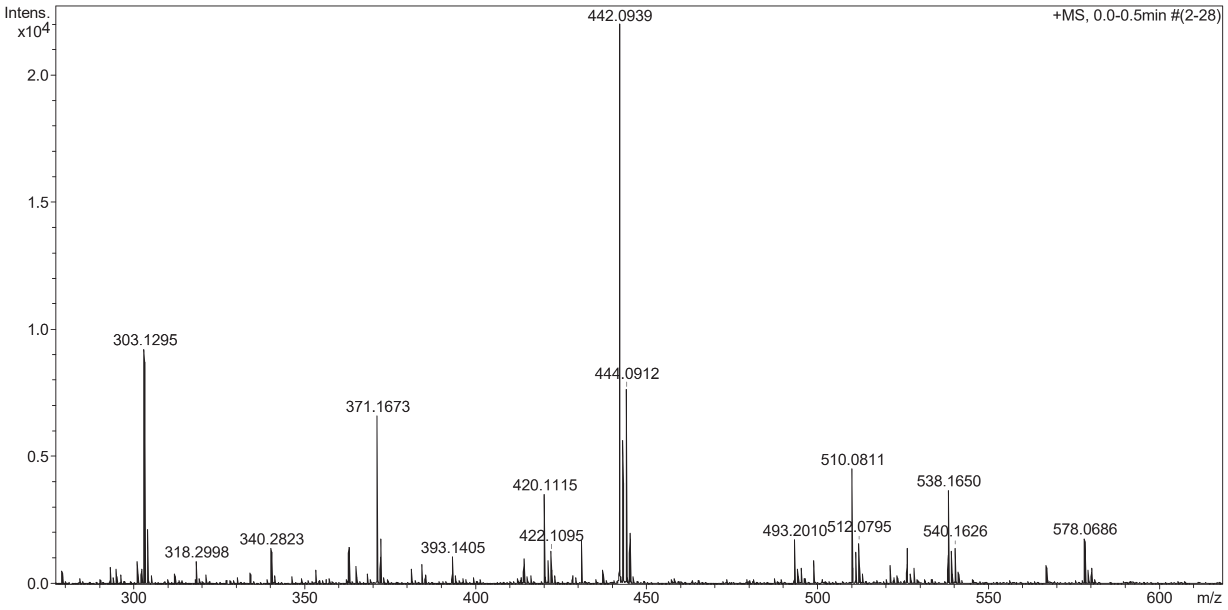

5g

Mass Spectrum Report

Analysis Info

Analysis Name	D:\Data\Data\2019\may\24\TOK-100.d	Acquisition Date	24.05.2019 13:43:53
Method	tune_low.m	Operator	Bruker Customer
Sample Name	TOK-100	Instrument / Ser#	micrOTOF 10223
Comment	MeOH		

Acquisition Parameter

Source Type	ESI	Ion Polarity	Positive	Set Nebulizer	1.0 Bar
Focus	Not active			Set Dry Heater	180 °C
Scan Begin	50 m/z	Set Capillary	4500 V	Set Dry Gas	8.0 l/min
Scan End	3000 m/z	Set End Plate Offset	-500 V	Set Divert Valve	Source

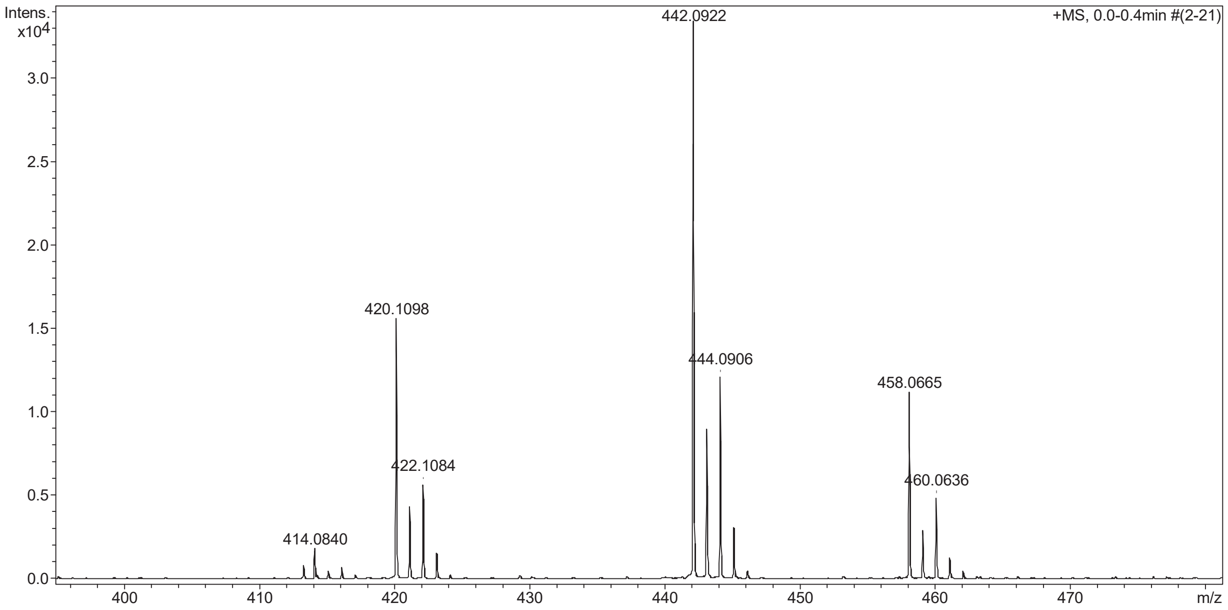

5h

Mass Spectrum Report

Analysis Info

Analysis Name	D:\Data\2019\january\21\TOK72.d	Acquisition Date	21.01.2019 14:34:19
Method	tune_low.m	Operator	Bruker Customer
Sample Name	TOK72	Instrument / Ser#	micrOTOF 10223
Comment	MeOH 300v		

Acquisition Parameter

Source Type	ESI	Ion Polarity	Positive	Set Nebulizer	0.4 Bar
Focus	Not active			Set Dry Heater	180 °C
Scan Begin	50 m/z	Set Capillary	4500 V	Set Dry Gas	4.0 l/min
Scan End	3000 m/z	Set End Plate Offset	-500 V	Set Divert Valve	Source

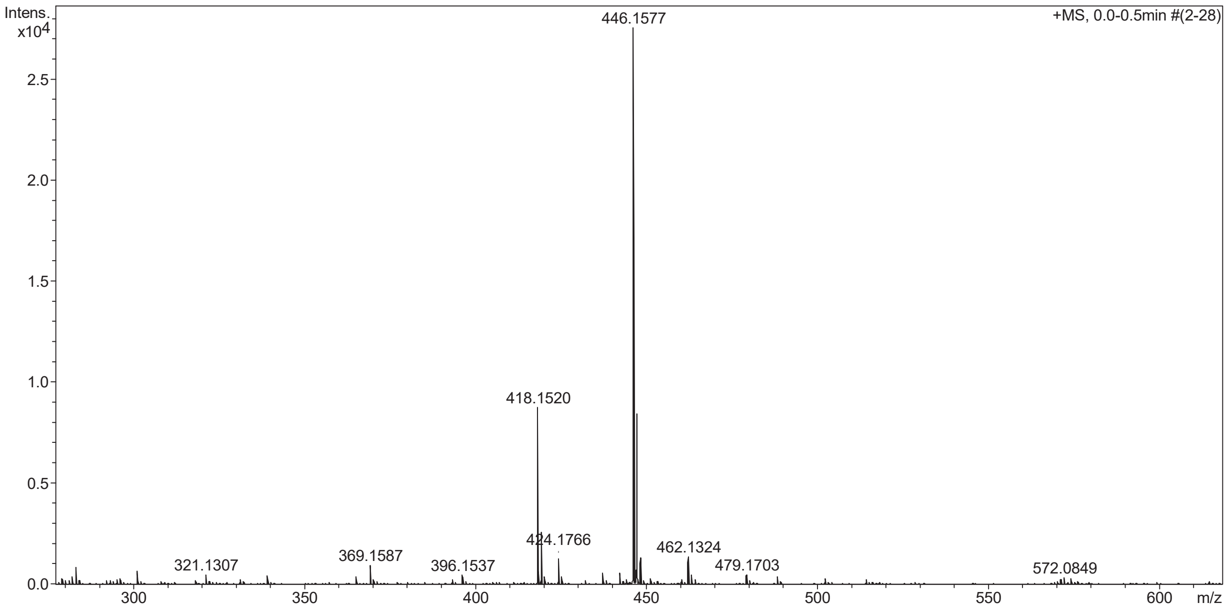

5i

Mass Spectrum Report

Analysis Info

Analysis Name	D:\Data\2018\december\3\TOK63.d	Acquisition Date	03.12.2018 13:40:50
Method	tune_low.m	Operator	Bruker Customer
Sample Name	TOK63	Instrument / Ser#	micrOTOF 10223
Comment	MeOH 50v		

Acquisition Parameter

Source Type	ESI	Ion Polarity	Positive	Set Nebulizer	0.4 Bar
Focus	Not active			Set Dry Heater	180 °C
Scan Begin	50 m/z	Set Capillary	4500 V	Set Dry Gas	4.0 l/min
Scan End	3000 m/z	Set End Plate Offset	-500 V	Set Divert Valve	Source

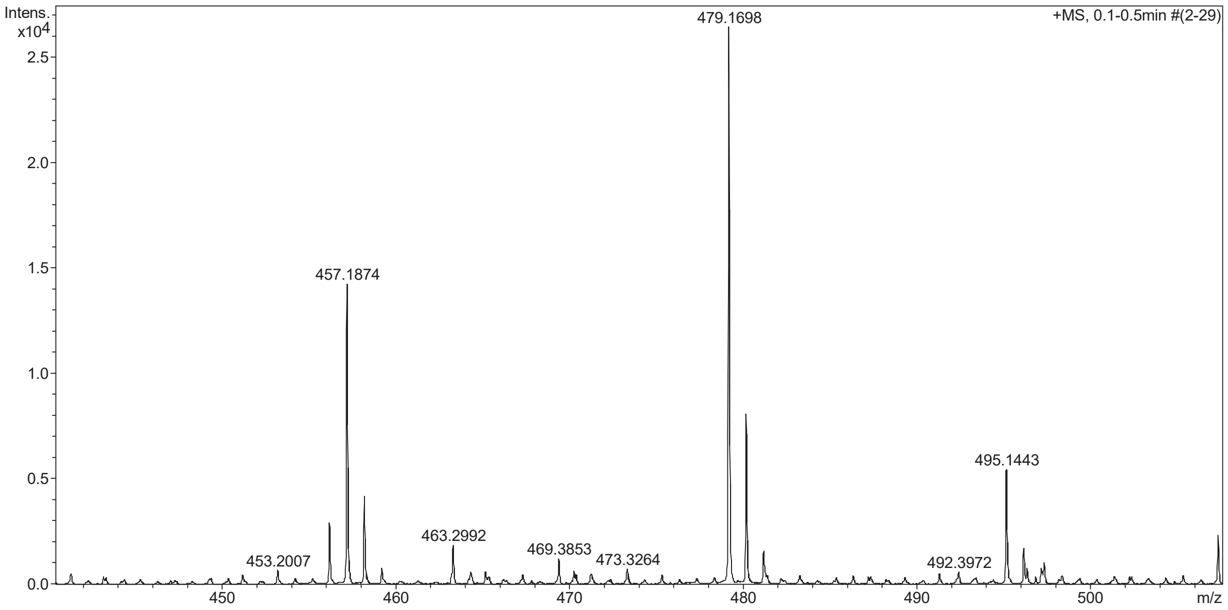

5j

Display Report

Analysis Info

Analysis Name	D:\Data\2021\june\22\TOK_141000001.d	Acquisition Date	22.06.2021 10:51:23
Method	tune_low.m	Operator	Bruker Customer
Sample Name		Instrument / Ser#	micrOTOF 10223
Comment			

Acquisition Parameter

Source Type	ESI	Ion Polarity	Positive	Set Nebulizer	0.4 Bar
Focus	Not active			Set Dry Heater	180 °C
Scan Begin	100 m/z	Set Capillary	4500 V	Set Dry Gas	4.0 l/min
Scan End	1000 m/z	Set End Plate Offset	-500 V	Set Divert Valve	Source

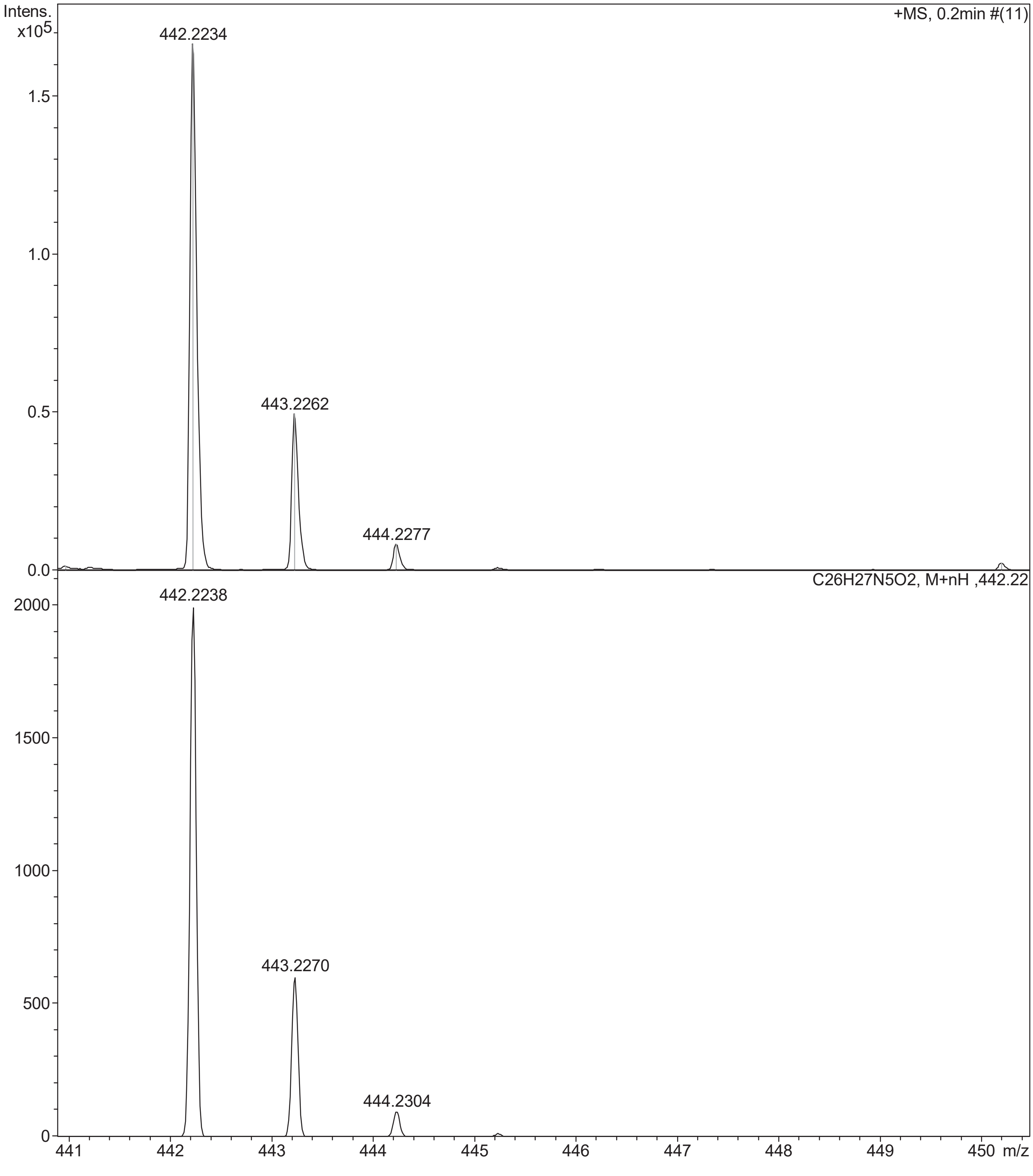

5k

Display Report

**Analysis Info**		Acquisition Date	17-Feb-22 12:13:32	
Analysis Name	D:\Data\Work\2022\02\17\GAI_18.d			
Method	20211118_tune_low_pos.m	Operator	BDAL@DE	
Sample Name		Instrument / Ser#	maXis	62
Comment				

Acquisition Parameter

Source Type	ESI	Ion Polarity	Positive	Set Nebulizer	0.3 Bar
Focus	Not active			Set Dry Heater	180 °C
Scan Begin	100 m/z	Set Capillary	3200 V	Set Dry Gas	4.0 l/min
Scan End	1000 m/z	Set End Plate Offset	-500 V	Set Divert Valve	Source

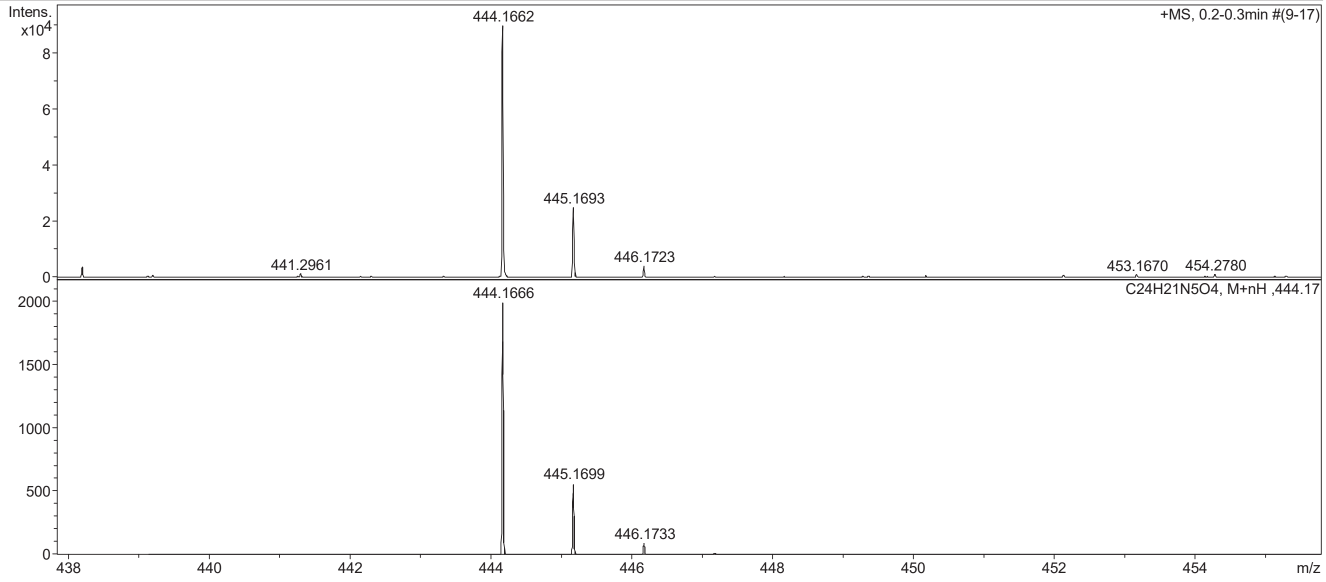

Create PDF files without this message by purchasing novaPDF printer (http://www.novapdf.com)

51

Display Report

Analysis Info

Analysis Name	D:\Data\2021\june\22\TOK_156000001.d	Acquisition Date	22.06.2021 10:57:16
Method	tune_low.m	Operator	Bruker Customer
Sample Name		Instrument / Ser#	micrOTOF 10223
Comment			

Acquisition Parameter

Source Type	ESI	Ion Polarity	Positive	Set Nebulizer	0.4 Bar
Focus	Not active			Set Dry Heater	180 °C
Scan Begin	100 m/z	Set Capillary	4500 V	Set Dry Gas	4.0 l/min
Scan End	1000 m/z	Set End Plate Offset	-500 V	Set Divert Valve	Source

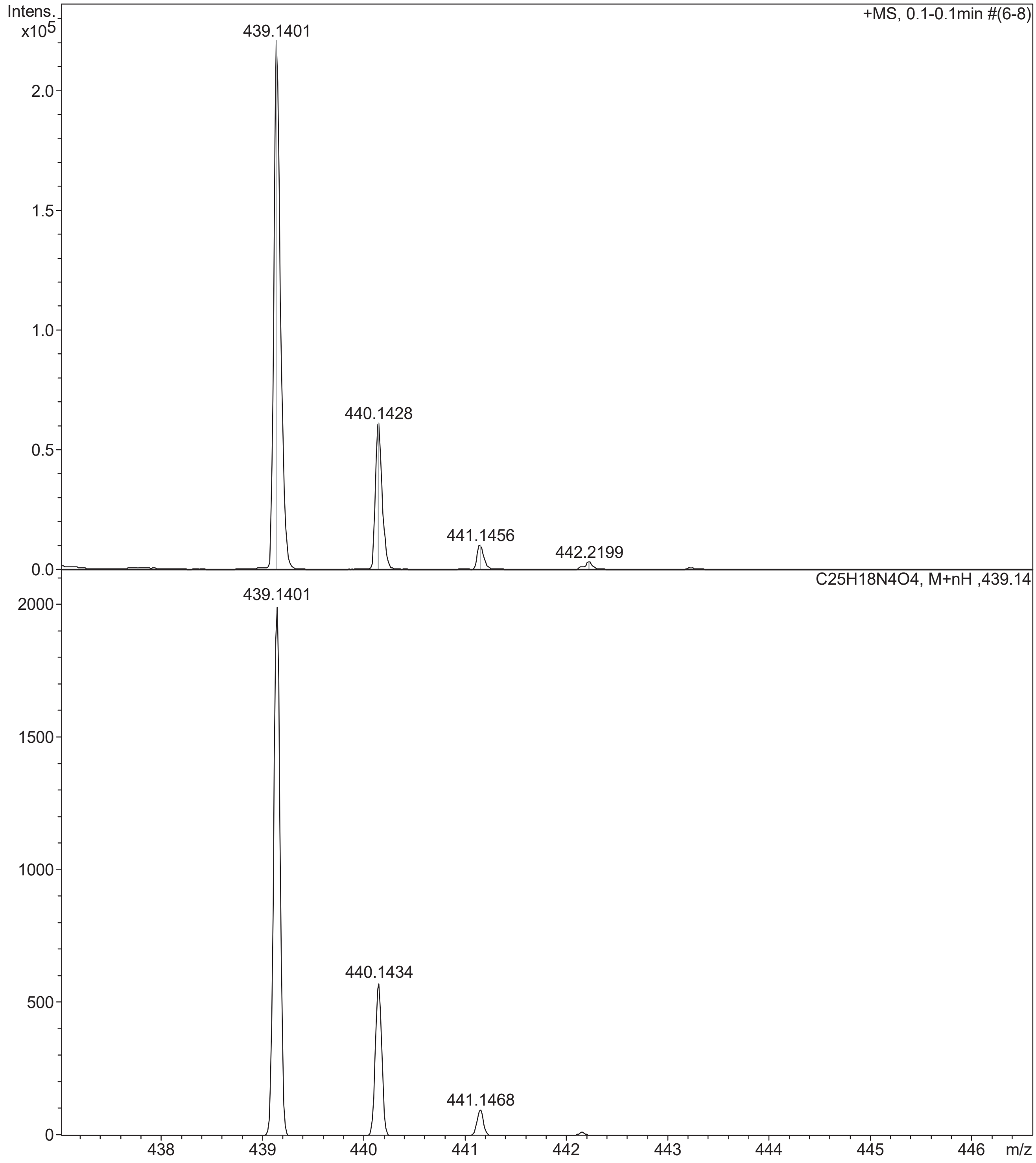

5m

Display Report

Analysis Info

Analysis Name	D:\Data\2021\june\22\TOK_157000001.d	Acquisition Date	22.06.2021 11:00:19
Method	tune_low.m	Operator	Bruker Customer
Sample Name		Instrument / Ser#	micrOTOF 10223
Comment			

Acquisition Parameter

Source Type	ESI	Ion Polarity	Positive	Set Nebulizer	0.4 Bar
Focus	Not active			Set Dry Heater	180 °C
Scan Begin	100 m/z	Set Capillary	4500 V	Set Dry Gas	4.0 l/min
Scan End	1000 m/z	Set End Plate Offset	-500 V	Set Divert Valve	Source

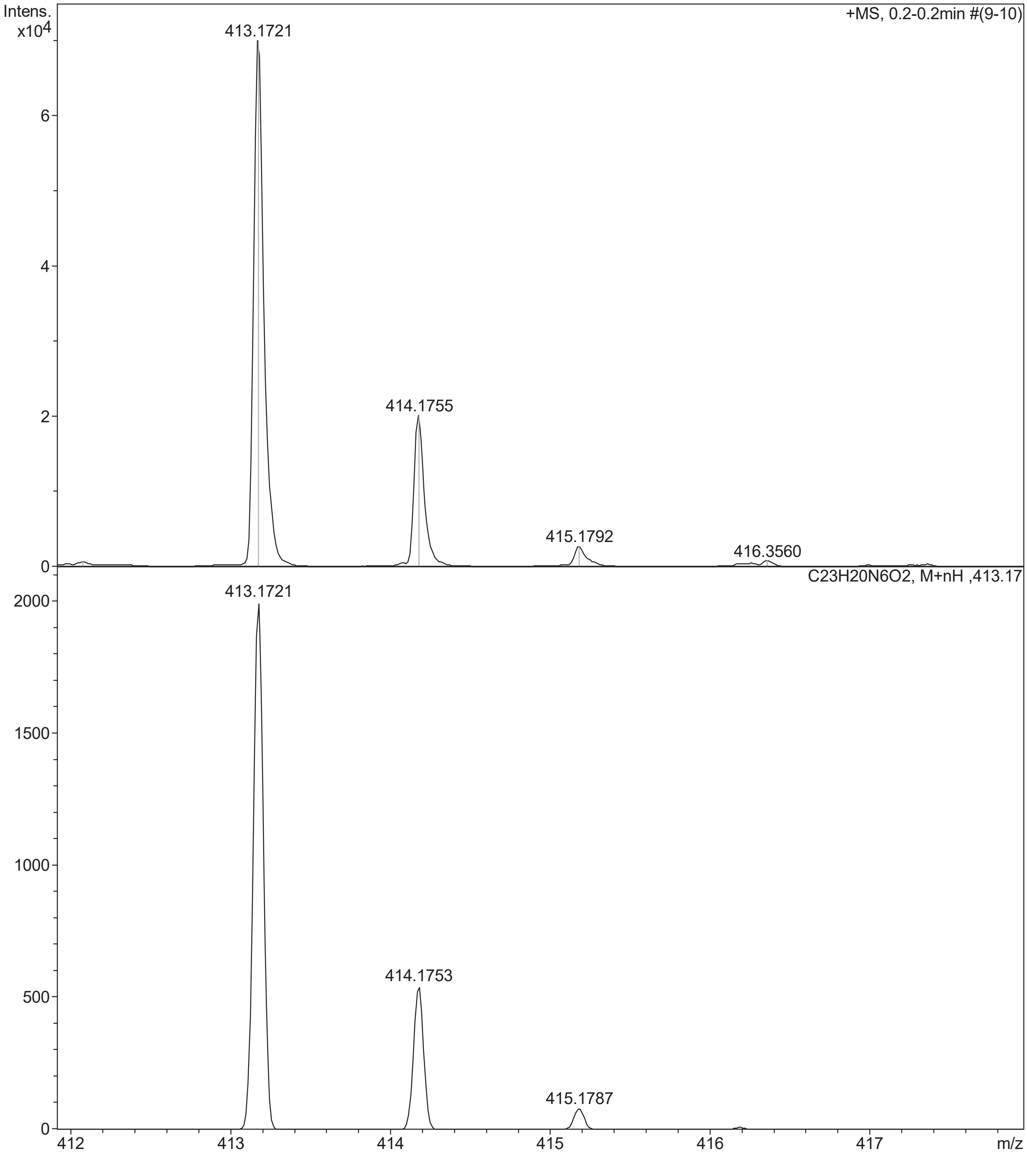

8a

Mass Spectrum Report

Analysis Info

Analysis Name	D:\Data\Data\2019\april\25\TOK134.d	Acquisition Date	25.04.2019 10:24:31
Method	tune_low.m	Operator	Bruker Customer
Sample Name	TOK134	Instrument / Ser#	micrOTOF 10223
Comment	MeOH		

Acquisition Parameter

Source Type	ESI	Ion Polarity	Positive	Set Nebulizer	1.0 Bar
Focus	Not active			Set Dry Heater	180 °C
Scan Begin	50 m/z	Set Capillary	4500 V	Set Dry Gas	8.0 l/min
Scan End	3000 m/z	Set End Plate Offset	-500 V	Set Divert Valve	Source

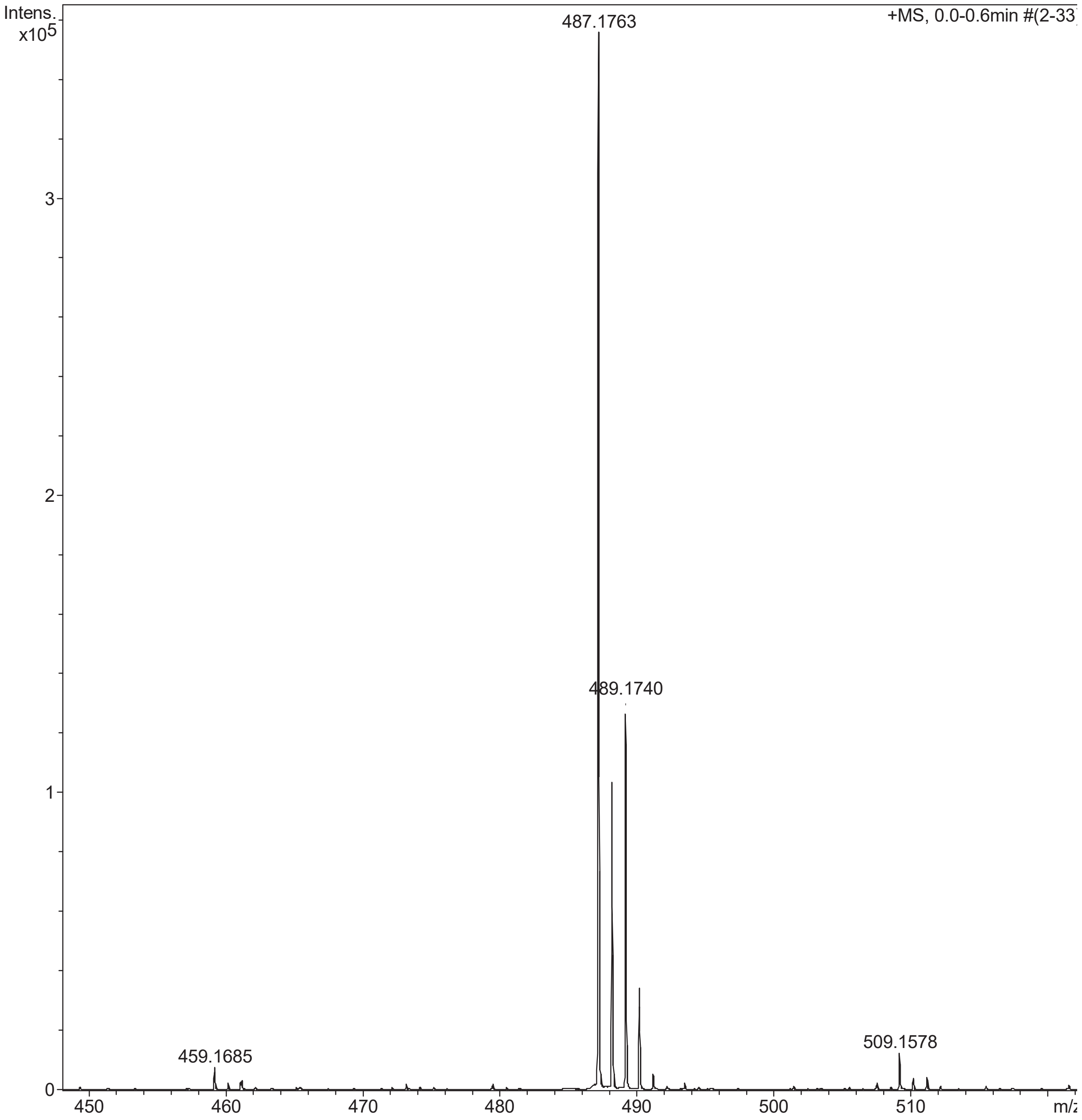

8b

Mass Spectrum Report

Analysis Info

Analysis Name	D:\Data\2019\december\20\GAI954.d	Acquisition Date	20.12.2019 18:13:57
Method	tune_low.m	Operator	Bruker Customer
Sample Name	GAI954	Instrument / Ser#	micrOTOF 10223
Comment			

Acquisition Parameter

Source Type	ESI	Ion Polarity	Positive	Set Nebulizer	0.4 Bar
Focus	Not active			Set Dry Heater	180 °C
Scan Begin	50 m/z	Set Capillary	4500 V	Set Dry Gas	4.0 l/min
Scan End	3000 m/z	Set End Plate Offset	-500 V	Set Divert Valve	Source

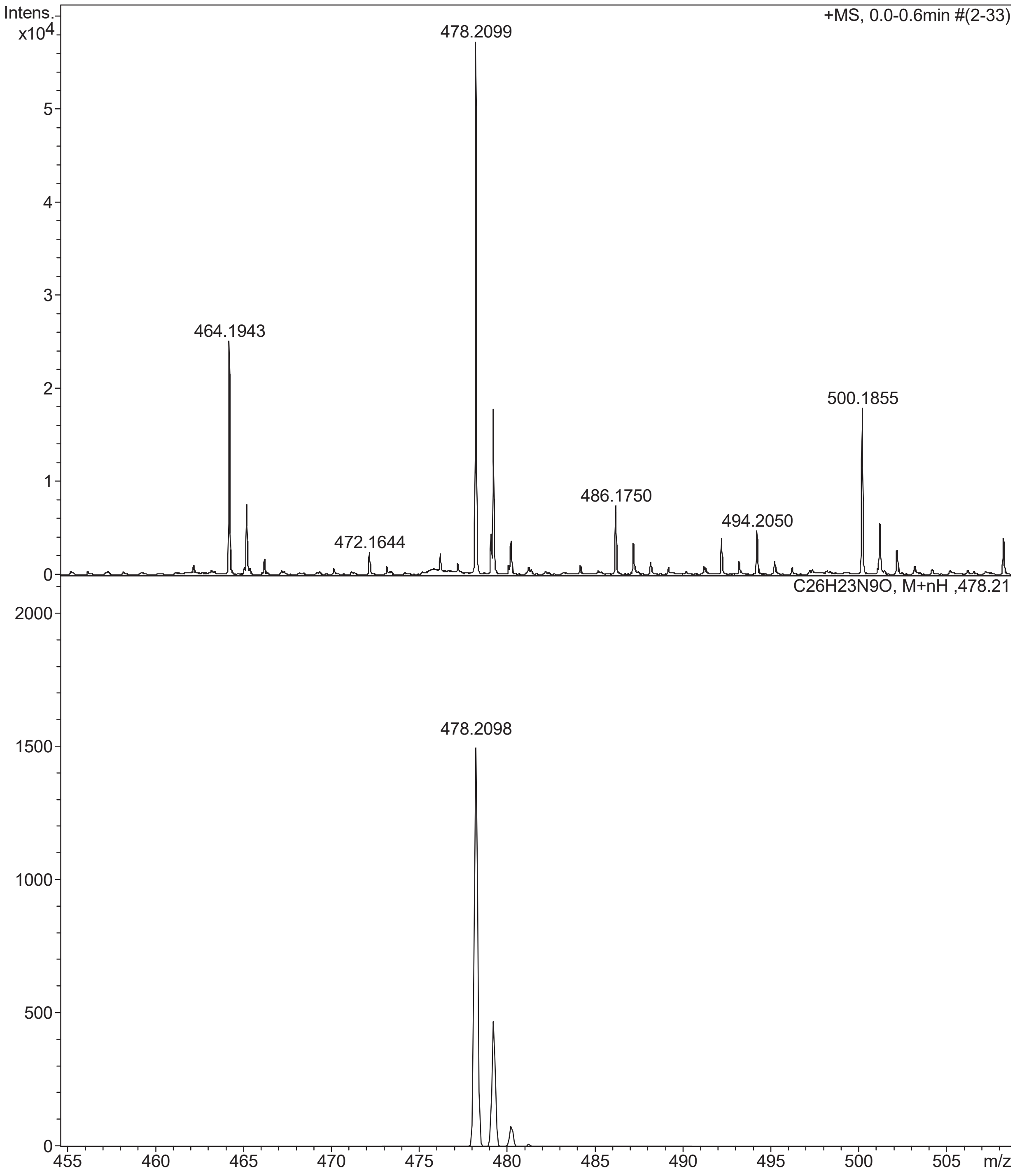

9a

Mass Spectrum Report

Analysis Info

Analysis Name	D:\Data\2019\december\23\GAI958.d	Acquisition Date	23.12.2019 9:47:53
Method	tune_low.m	Operator	Bruker Customer
Sample Name	GAI958	Instrument / Ser#	micrOTOF 10223
Comment	MeOH		

Acquisition Parameter

Source Type	ESI	Ion Polarity	Positive	Set Nebulizer	0.4 Bar
Focus	Not active			Set Dry Heater	180 °C
Scan Begin	50 m/z	Set Capillary	4500 V	Set Dry Gas	4.0 l/min
Scan End	3000 m/z	Set End Plate Offset	-500 V	Set Divert Valve	Source

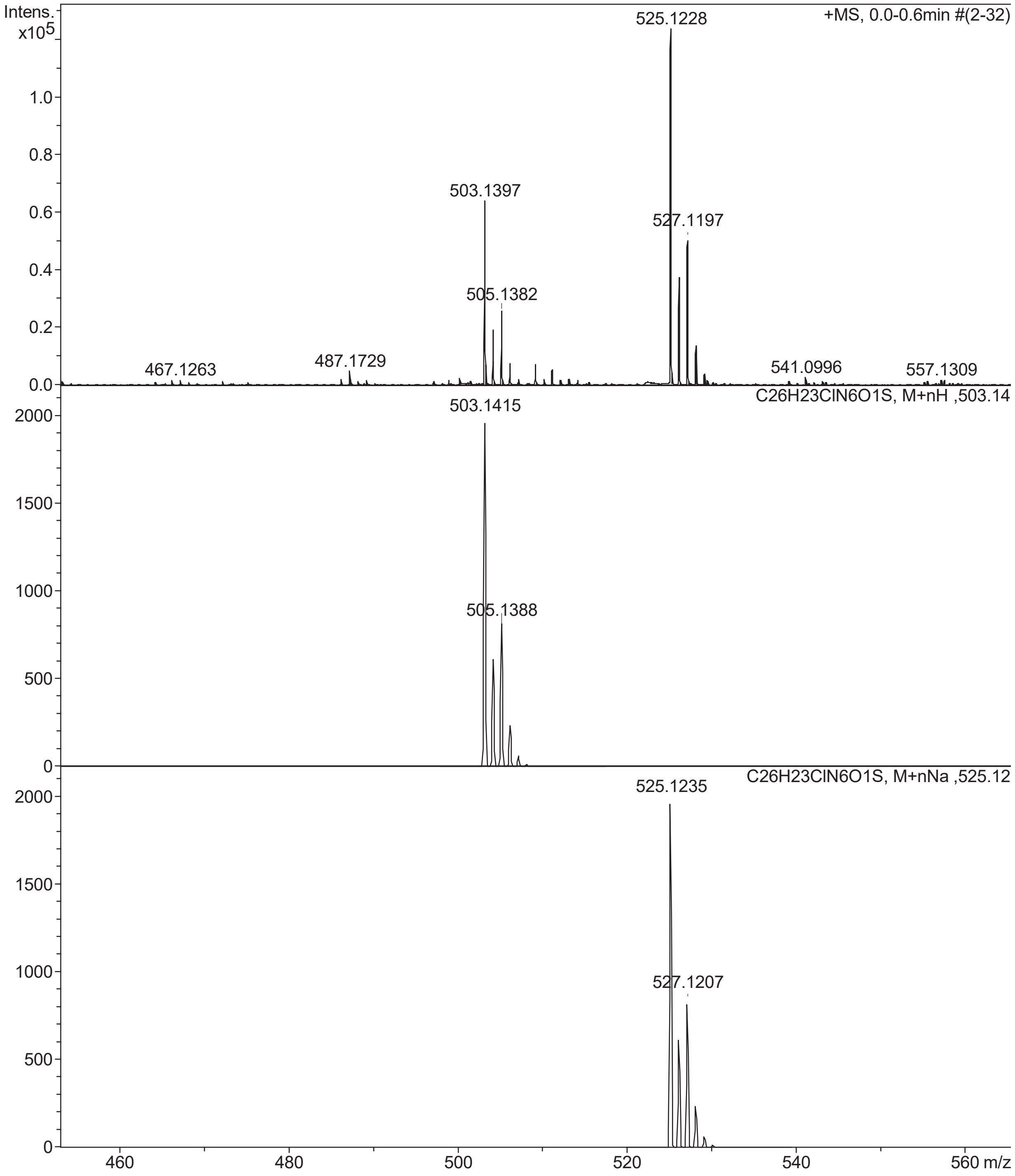

Supplement: Supplementary file 1 [file molecules-27-03191-s001.zip › molecules-1726117-supplementary.pdf]
